# Association of age at first sexual intercourse and lifetime number of sexual partners with cardiovascular diseases: a bi-directional Mendelian randomization study

**DOI:** 10.3389/fcvm.2023.1267906

**Published:** 2023-12-07

**Authors:** Chengui Zhuo, Lei Chen, Qiqi Wang, Haipeng Cai, Zujin Lin, Huili Pan, Meicui Wu, Yuxiang Jin, Hong Jin, Liangrong Zheng

**Affiliations:** ^1^ Department of Cardiology, Taizhou Central Hospital (Taizhou University Hospital), Taizhou, China; ^2^ Zhejiang Provincial Center for Drug and Medical Device Procurement, Hangzhou, China; ^3^ Department of Cardiology and Atrial Fibrillation Center, the First Affiliated Hospital, College of Medicine, Zhejiang University, Hangzhou, China

**Keywords:** age at first sexual intercourse, lifetime number of sexual partners, cardiovascular diseases, causal association, Mendelian randomization

## Abstract

**Background:**

Limited studies have explored the association between sexual factors [age at first sexual intercourse (AFS) and lifetime number of sexual partners (LNSP)] and cardiovascular diseases (CVDs), leaving the causality inconclusive.

**Methods:**

We performed a bi-directional Mendelian randomization (MR) study to investigate the causality between sexual factors and CVDs, including coronary artery disease, myocardial infarction, atrial fibrillation (AF), heart failure (HF), and ischemic stroke (IS). Single-nucleotide polymorphisms (SNPs) for sexual factors were extracted from the UK Biobank. Statistics for each CVD were derived from two different databases. MR estimates were calculated per outcome database and were combined through meta-analysis. Several complementary sensitivity analyses were also performed.

**Results:**

The primary analysis suggested that AFS was causally associated with the risk of CVDs; the odds ratios (ORs) ranged from 0.686 [95% confidence interval (CI), 0.611–0.770] for HF to 0.798 (95% CI, 0.719–0.886) for AF. However, the association between AFS and IS (OR, 0.844; 95% CI, 0.632–1.126) was not consistent in the meta-analysis after excluding SNPs related to confounders. Moreover, non-significant associations were found between LNSP and CVDs. Reverse direction MR analysis showed that CVDs were not associated with sexual factors.

**Conclusions:**

Genetic evidence suggested that AFS was causally associated with the risk of CVDs except for IS, whereas non-significant association of LNSP with CVDs was detected. Further investigation into AFS could be warranted in preventing the progression of CVDs.

## Background

Globally, cardiovascular diseases (CVDs) remain the principal contributor to mortality, responsible for nearly 32% of all deaths (1–3). The heavy burden of CVDs underscores the importance of identifying risk factors for prevention. In recent decades, reproductive behaviors have been increasingly implicated as significant factors in cancers, respiratory disease, and CVDs.

Reproductive behaviors are heritable traits, encompassing age at menarche and menopause, age at birth, age at first sexual intercourse (AFS), lifetime number of sexual partners (LNSP), and more (4). To date, several of these reproductive behaviors have been demonstrated as risk factors for cardiovascular diseases (4–8). For instance, a younger age at menarche and menopause is positively associated with the risk of cardiovascular diseases (5–7). Compared with women experiencing menarche at 13 years and menopause at 45 years or older, the adjusted relative risk of coronary artery disease (CAD) for early menarche (≤10 years) and early menopause (<45 years) is 1.27 and 1.50, respectively (5, 7). Previous studies have also shown that the age at first birth is positively associated with CVDs (8, 9). However, limited studies have considered risky sexual factors (such as younger AFS and higher LNSP) in relation to CVDs. Ngueta and Ndjaboue first explored the relationship between early sexual activity and hypertension (10), but it remains unclear whether risky sexual factors are causally associated with other CVDs such as CAD and heart failure (HF).

Sexual activity increasingly occurs at an earlier age, with one-third of contemporary British adolescents having initial sexual intercourse by the age of 16. Early sexual activity could have long-term implications for adult health (11). As a result, numerous programs on adolescent sexuality education aim to delay the onset of sexual activity to improve health-related outcomes. However, to validate that this focus is warranted, it is crucial to demonstrate that risky sexual activity does indeed cause harmful health impacts.

Mendelian randomization (MR) is a novel epidemiological approach used to infer the causal association of exposures and outcomes by using genetic variants from genome-wide association studies (GWASs) as instrumental variables (IVs) (12, 13). MR studies had been successfully applied in the strategies of CVD prevention (14–16), such as the development of proprotein convertase subtilisin/kexin type 9 (PCSK9) inhibitors, which significantly decreased the risk of CVD (16). Previous MR studies also provided considerable evidence that CVD was causally related to traditional risk factors, which had already been suggested by observational studies, including hypertension, adiposity, type 2 diabetes, and so on (17–19). However, few MR studies have evaluated the causality between risky sexual factors and CVDs. Therefore, in this study, bi-directional MR analysis is first conducted to explore the causal association between sexual factors (AFS and LNSP) and cardiovascular diseases, including CAD, myocardial infarction (MI), HF, atrial fibrillation (AF), and ischemic stroke (IS).

## Methods

### Study design

In the present study, we applied a bi-directional two-sample MR study to comprehensively explore the causality between two sexual factors and CVDs. In the first stage, we evaluated whether genetically predicted sexual factors were causally related to CVDs. In the second stage, we also assessed whether genetically predicted CVDs were causally associated with sexual factors (Supplementary Figure S1). Two-sample MR is based on three principal assumptions: First, the single-nucleotide polymorphisms (SNPs) selected as the IVs are strongly related to exposures. Second, IVs should be independent of any confounders. Third, IVs affect the outcome exclusively via the exposure.

### Data sources

All summary statistics for sexual factors and CVDs were sourced from public GWASs, as shown in Supplementary Table S1. For sexual factors, we obtained data from the GWAS meta-analysis of the UK Biobank, including 397,338 and 378,882 pooled individuals for AFS and LNSP, respectively. Summary statistics for CVDs were extracted from the Coronary Artery Disease Genome-wide Replication and Meta-analysis plus The Coronary Artery Disease Genetics (CARDIoGRAMplusC4D) consortium for CAD and MI (20), the GWAS by Nielsen et al. for AF (21), the GWAS by Shah et al. for HF (22), and the MEGASTROKE consortium for IS (23). We also applied summary statistics from the FinnGen study for replication purposes. The FinnGen study is a medical project that started in 2017, combining genomic information from Finnish biobanks and health data from Finnish health registries (24).

### IVs selection

The present study identified independent SNPs significantly associated with exposure (*p* < 5 × 10^−8^) by applying the PLINK clumping method (*r*^2 ^< 0.001 and window size = 10 Mb). If the identified SNPs for the exposures were absence in the GWAS of the outcome, proxy SNPs (linkage disequilibrium *r*^2^ > 0.8) would be searched using an online tool (https://ldlink.nci.nih.gov/). The *F*-statistic was computed to quantify the strength of selected SNPs (25). A larger *F*-statistic (>10) indicates stronger strength of SNPs. *A priori* statistical power was also conducted.

To rule out any potential confounders, we searched for each IV and its proxies in PhenoScanner to evaluate any previously demonstrated associations (*p*-value < 5 × 10^−6^) with plausible confounders or CVDs. In the present study, the confounders included tobacco use, alcohol use, body mass index, lipid profile, and diabetes. We would repeat the MR analysis after excluding the SNPs related to confounders.

### Statistical analysis

After extracting and harmonizing the data, we applied the fixed-effects inverse variance-weighted (IVW) method as the primary analysis to assess the causal association between exposures and outcomes. The IVW method could combine the SNP-specific Wald ratio estimates and provide an unbiased estimate (26). Moreover, we further used Cochran's *Q* to evaluate the presence of heterogeneity. In cases with evidence of heterogeneity (*p*-value < 0.05), we conducted the random-effects IVW method.

In addition, we also conducted a complementary sensitivity analysis to test the robustness of our findings. First, the simple and weighted median methods were performed to validate the results. For the weighted median, it tends to give valid estimates when more than half of the weight is obtained from valid SNPs (27). Second, the results of MR might be biased due to the directional pleiotropy. Therefore, the MR-Egger method was applied, and a *p*-value >0.05 for the MR-Egger intercept indicated the absence of directional pleiotropy. In addition, *I*^2^_GX_ was used to evaluate the suitability of MR-Egger, and an *I*^2^_GX_ > 95% was desired (28). Scatter plots were also conducted to show the effects estimated by each method. Third, MR pleiotropy residual sum and outlier (MR-PRESSO) were conducted to evaluate potential pleiotropic effects (outlier IVs) and provided outlier-adjusted estimates by excluding outlier IVs (29). Next, the leave-one-out analysis was conducted to guarantee that causality was not influenced by the particular IV. Finally, replication MR analyses were conducted using different outcome GWAS datasets (the FinnGen study), and then all MR estimates were meta-analyzed to generate the pooled estimates for each exposure on outcomes. A two-sided *p*-value below 0.05 indicated a statistically significant causal association. All MR analyses were performed through R Version 3.6.3 with the “TwoSampleMR,” “MR-PRESSO,” and “meta” packages.

## Results

Summary information of selected SNPs is shown in Supplementary Tables S2–S5. In total, they explained nearly 2.2% and 0.1% of the phenotypic variability of AFS and LNSP, respectively. The *F*-statistic for all SNPs was above 10, suggesting that they were strong enough to minimize bias from weak instrument bias. SNPs that were detected to be related to confounders or CVDs are shown in Supplementary Tables S6,S7. Cochran's *Q* identified the presence of heterogeneity in several MR results (Supplementary Table S8), and as a result, random-effects IVW methods were performed. Given a type I error of 0.05, the power calculation results of two sexual factors are provided in Supplementary Table S9. Scatter plots are also presented in Supplementary Figures S2–S5.

### Age at first sexual intercourse and CVDs

**Figure 1:**
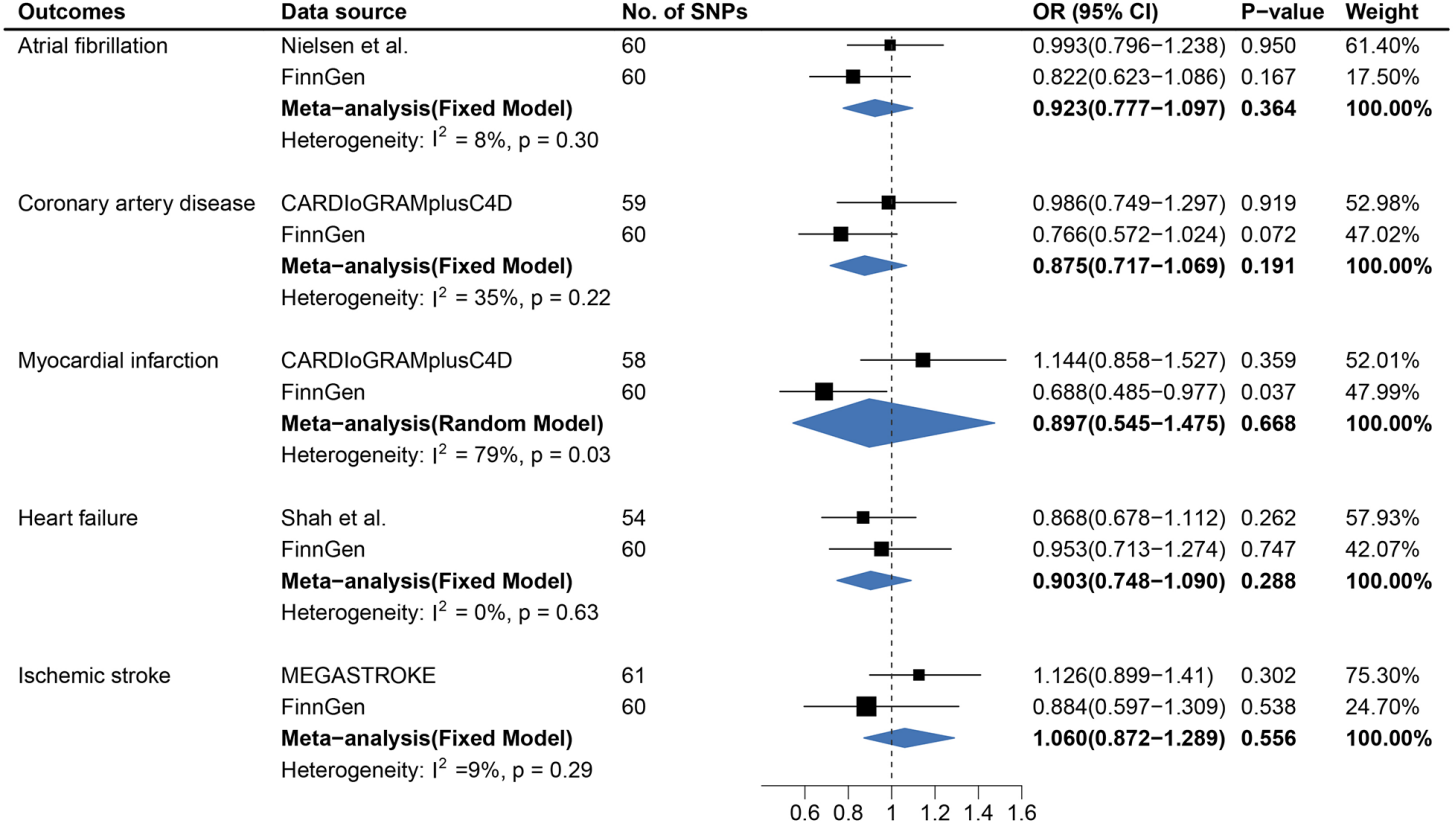
Causal association between age at first sexual intercourse and cardiovascular diseases. Estimated odds ratios of cardiovascular disease per one-unit increase in age at first sexual intercourse were determined from the primary IVW analysis. These estimations were done for each outcome data source separately, and then combined across the two data sources using a meta-analysis.

Overall, the primary IVW analysis suggested significant inverse associations between age at first sexual intercourse and the risk of coronary artery disease [odds ratio (OR), 0.704; 95% confidence interval (CI), 0.610–0.812], myocardial infarction (OR, 0.727; 95% CI, 0.627–0.843), heart failure (OR, 0.686; 95% CI, 0.611–0.770), atrial fibrillation (OR, 0.798; 95% CI, 0.719–0.886), and ischemic stroke (OR, 0.737; 95% CI, 0.657–0.827), as shown in Figure 1. In the reverse direction MR analysis, CVDs showed non-significant associations with age at first sexual intercourse (Supplementary Table S10). Most OR estimates were consistent using different sensitivity analyses but for the MR-Egger method (Figure 3). Owing to the lower precision of the MR-Egger method, we noticed that its estimates differed from the other MR analyses and the CIs were wider than in other methods (30). The intercept of MR-Egger regression (Supplementary Tables S11,S12) detected no directional pleiotropy except for the analysis of age at first sexual intercourse on AF (intercept = 0.992; 95% CI, 0.985–0.999; *p *= 0.028). Although the MR-PRESSO method identified some outlier SNPs, the estimate did not change significantly after correction (Figure 3 and Supplementary Tables S13,S14). In addition, we further excluded the SNPs associated with any confounders, and the analysis of the remaining SNPs observed broadly similar results (Supplementary Figures S6,S8). Moreover, the results of the leave-one-out analyses are displayed in Supplementary Figures S9–S13,S19–S23.

In a replication study based on the FinnGen data, there was significant evidence of causal associations between AFS and the risk of CAD, MI, AF, and HF, whereas statistically non-significant associations were detected between AFS and IS.

In the meta-analysis, the pooled OR before and after removing SNPs related to confounders is shown in Figure 1 and Supplementary Figure S6. The causal association for AFS and IS (OR, 0.844; 95% CI, 0.632–1.126) was not replicated in the meta-analysis after excluding the SNPs associated with confounders (Supplementary Figure S6).

### Lifetime number of sexual partners and CVDs

**Figure 2:**
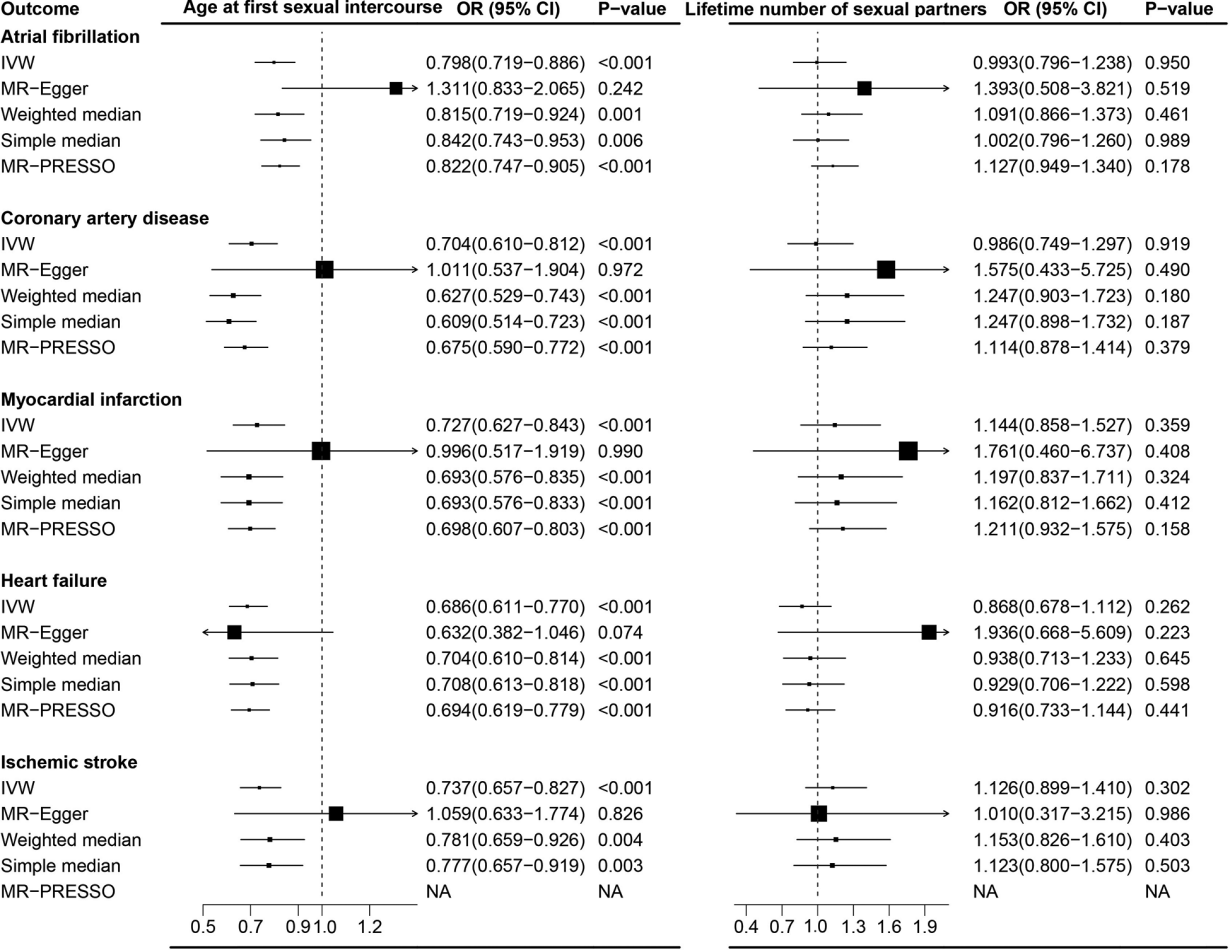
Causal association between lifetime number of sexual partners with cardiovascular diseases. Estimated odds ratios of cardiovascular disease per one-unit increase in lifetime number of sexual partners were determined from the primary IVW analysis. These estimations were done for each outcome data source separately, and then combined across the two data sources using a meta-analysis.

**Figure 3:**
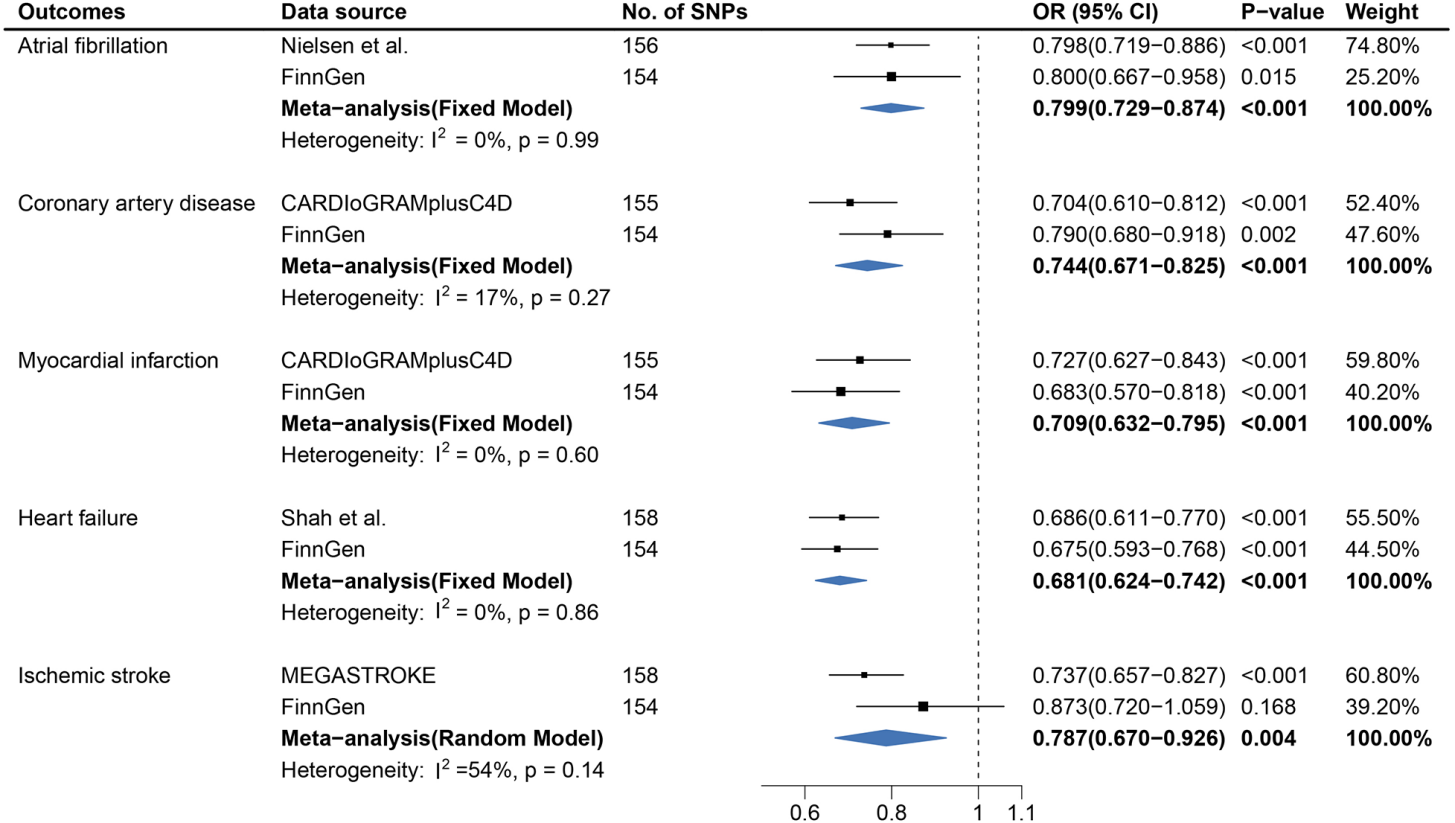
Complementary sensitivity analysis of the association between sexual factors and cardiovascular diseases.

In contrast to the findings from AFS, no causal association between genetically predicted LNSP and CVDs was observed except for MI (Figure 2 and Supplementary Figure S7). Genetically predicted LNSP was positively associated with MI (OR, 1.456; 95% CI: 1.101–1.926; *p *= 0.008) after excluding the SNPs related to confounders. However, the association was not consistent in sensitivity analyses and the pooled OR for MI was 1.147 (95% CI: 0.687–1.917). The details of sensitivity analysis results are shown in Figure 3 and Supplementary Figure S8. Reverse direction MR analysis identified that CVDs were not associated with LNSP (Supplementary Tables S10,S14). The results of the leave-one-out analyses are shown in Supplementary Figures S14–S18,S24–S28.

## Discussion

To the best of our knowledge, the present MR study is the first to systematically explore the bi-directional association between sexual factors (including AFS and LNSP) and the risk of CVDs. Our results identified that AFS could be causally associated with CVDs, with the exception of IS, while LNSP showed no association. The results from complementary sensitivity analyses were generally consistent. Moreover, reverse direction MR analysis suggested that genetically predicted CVDs were unlikely to be causal determinants of sexual factors.

Our study suggested that genetic predisposition to AFS was causally associated with the risk of CAD, MI, AF, and HF, with the findings remaining robust in replication and meta-analysis. Few epidemiological studies have considered AFS in relation to CVDs (10, 31). Nikpay et al. conducted a phenome-wide search and suggested that AFS was negatively associated with CAD in both males and females (31). In addition, another cohort study involving 39,788 individuals initially confirmed that a younger age at first sexual intercourse was significantly linked to hypertension (10). The exact mechanisms underpinning this association are unclear but may involve both environmental and genetic factors. Previous research has shown that early sexual debut correlates with adverse environmental conditions, including lower levels of education, as well as greater exposure to smoking, drinking, and illicit drug use (32). Furthermore, early sexual debut typically contributes to poorer quality partner relationships (33). People dissatisfied with their relationships tend to have an increased risk of CVDs (34, 35). In addition, studies have already explored the influence of exercise on adverse cardiac remodeling, and intense exercise may even cause exercise-induced cardiomyopathy (36, 37). Early sexual activity (<16-year-old boys) is associated with more physical activity, which may potentially induce the development of cardiomyopathy and ultimately lead to cardiovascular disease (38). Beyond environmental factors, the potential role of genetics should also be emphasized. The dopamine D4 receptor gene has been linked to younger age at first sexual intercourse (39, 40) and elevated blood pressure, which directly contributes to the development of CVDs (41).

The current results on the causal association of AFS with IS are still controversial and incomplete. A previous MR study (42) reported that genetically predicted AFS was significantly associated with IS. However, another MR study showed no causality (43). This divergence might be attributed to pleiotropic bias, statistical analysis, and different datasets. In our study, we found that age at first sexual intercourse was negatively associated with IS in the primary analysis. However, after excluding SNPs associated with relevant confounders, the results were inconsistent. Several MR studies have already demonstrated the causal association between poor environmental conditions (such as tobacco, drug, and alcohol use) and IS (44, 45). Early initiation of sexual intercourse typically occurs in adolescence and is often linked to poor environmental conditions, which may directly impact IS rather than through age at first sexual intercourse, thereby generating bias in MR analysis (32).

No previous study has directly investigated the causal association between LNSP and CVDs using bi-directional MR, making it difficult to compare our present results with those of former MR studies. Lifetime number of sexual partners has previously been applied as an indicator of reproductive behaviors, as the number of sexual partners reflects mating success and thus potential reproductive success (46–48). Prior epidemiological and MR studies have provided evidence and confirmed that reproductive behaviors could play a crucial role in the development of CVDs (4–8). However, our study did not find a clear causal association between LNSP and CVDs. It is worth considering that contraception, commonly used in developed countries for population control, may influence reproductive success and affect the results (49). Notably, contraception allows for the decoupling of sexual and reproductive partners (47). Therefore, the causal association between the lifetime number of reproductive partners and CVDs needs further exploration.

Our study has several remarkable strengths. First, the MR analysis is less susceptible to being influenced by confounders and other biases than standard regression analysis, thereby strengthening the reliability of causal inference (50, 51). Furthermore, we conducted a complementary sensitivity analysis to confirm the robustness of our results. Second, to avoid potential confounders that could impact the causal association, we repeated the analysis after excluding potentially pleiotropic SNPs related to body mass index, lipid profile, diabetes, and tobacco, drug, and alcohol use. Third, we meta-analyzed the potential causal effects in the current study using different databases, and the results were generally consistent.

Nonetheless, a number of limitations need consideration. First, in our study, MR analysis was conducted under the linear assumption to evaluate the causality between sexual factors and CVDs. We were unable, however, to test the non-linear effect with the present summary-level data. Future work should focus on non-linear MR analysis using individual-level data. Second, the partial overlap between sexual factors and CVD samples may create a weak instrument bias in our results. We applied the *F*-statistics to quantify weak IVs, and *F*-statistics for all IVs were above 10, thus avoiding weak instrument bias. Third, IVs for lifetime number of sexual partners explained only a small fraction of the phenotypic variance, with an *R*^2^ of 0.6%. Larger GWAS of lifetime number of sexual partners will facilitate MR studies with higher statistical power to examine CVD risks. Fourth, our study subjects were largely restricted to European ancestry, which restricts the generalizability of our results to other ancestry. It is necessary to validate our results in different ancestries. Finally, age at first sexual intercourse was causally associated with CVDs except for IS, but we could not absolutely exclude the possibility that the causal association between AFS and IS was not big enough to be detected even within the large sample. However, such a potential association would be exceedingly small and is unlikely to lead to a clinically relevant reduction of IS risk, as achieved by other strategies, such as smoking cessation, blood glucose, and lipid lowering.

## Conclusion

According to the present study, age at first sexual intercourse could be causally associated with the risk of CVDs, except for IS, while no significant causal effect of CVDs on AFS was detected. Furthermore, LNSP showed no causal association with CVDs in bi-directional MR analysis. These conclusions can serve as a reference for adolescent sexuality education to aid sexually active adolescents in preventing the progression of CVDs.

## Data Availability

The original contributions presented in the study are included in the article/**Supplementary Material**, further inquiries can be directed to the corresponding author.
