# Supplementary material for: Association of age at first sexual intercourse and lifetime number of sexual partners with cardiovascular diseases: a bi-directional Mendelian randomization study
Source: Front Cardiovasc Med. 2023 Dec 7;10:1267906. doi: 10.3389/fcvm.2023.1267906 (PMC10749299; doi:10.3389/fcvm.2023.1267906)

Supplementary Material

[Supplementary Table 1. Details of the studies and datasets included in the analyses.-------------------------------------------------------------------------------------------------1](#_Toc17807)

[Supplementary Table 2. Genome-wide significant SNPs for age at first sexual intercourse.-----------------------------------------------------------------------------------------3](#_Toc25721)

[Supplementary Table 3. Association of genome-wide significant SNPs for age at first sexual intercourse with cardiovascular diseases.------------------------------------1](#_Toc7251)1

[Supplementary Table 4. Genome-wide significant SNPs for lifetime number of sexual partners.----------------------------------------------------------------------------------20](#_Toc8970)

Supplementary Table 5. Association of genome-wide significant SNPs for lifetime number of sexual partners with cardiovascular diseases.------------------------------23

Supplementary Table 6. Evidence of association (p<5*10-6) of the SNPs used as genetic variants for Mendelian randomization analyses of age at first sexual intercourse with confounders or CVDs in the PhenoScanner.----------------------------------------------------------------------------------------------------------------------------26

Supplementary Table 7. Evidence of association (p<5*10-6) of the SNPs used as genetic variants for Mendelian randomization analyses of lifetime number of sexual partners with confounders or CVDs in the PhenoScanner.-------------------------------------------------------------------------------------------------------------------------------35

[Supplementary Table 8. Heterogeneity for the Mendelian randomization analysis.--------------------------------------------------------------------------------------------------3](#_Toc21892)8

Supplementary Table 9. Sample size and priori power calculations in Mendelian randomization study of sexual factors and risk of cardiovascular disease.------------39

Supplementary Table 10. Association of CVDs with age at first sexual intercourse and lifetime number of sexual partners using Mendelian randomisation.-----------40

Supplementary Table 11. MR-Egger pleiotropy test for all SNPS.----------------------------------------------------------------------------------------------------------------------41

Supplementary Table 12. MR-Egger pleiotropy test after removing SNPs associated with confounders or CVDs.--------------------------------------------------------------42

Supplementary Table 13. SNPs excluded from the outlier corrected MR-PRESSO analyses between age at first sexual intercourse and lifetime number of sexual partners with CVD.----------------------------------------------------------------------------------------------------------------------------------------------------------------------------43

Supplementary Table 14. SNPs excluded from the outlier corrected MR-PRESSO analyses between CVD with age at first sexual intercourse and lifetime number of sexual partners.---------------------------------------------------------------------------------------------------------------------------------------------------------------------------------45

Supplementary Figure 1. The diagram of bi-directional MR analysis.-----------------------------------------------------------------------------------------------------------------46

Supplementary Figure 2. Scatter plots for the causal association of age at first sexual intercoursewith outcomes. -------------------------------------------------------------47

Supplementary Figure 3. Scatter plots for the causal association of lifetime number of sexual partners with outcomes. ------------------------------------------------------48

Supplementary Figure 4. Scatter plots for the causal association of age at first sexual intercourse with outcomes after removing the SNPs associated with confounders or CVDs.-----------------------------------------------------------------------------------------------------------------------------------------------------------------------------------------49

Supplementary Figure 5. Scatter plots for the causal association of lifetime number of sexual partners with outcomes after removing the SNPs associated with confounders or CVDs.-------------------------------------------------------------------------------------------------------------------------------------------------------------------------50

Supplementary Figure 6. Causal association between age at first sexual intercourse with cardiovascular diseases after removing the SNPs associated with confounders or CVDs.-----------------------------------------------------------------------------------------------------------------------------------------------------------------------------------------51

Supplementary Figure 7. Causal association between lifetime number of sexual partners with cardiovascular diseases after removing the SNPs associated with confounders or CVDs.-------------------------------------------------------------------------------------------------------------------------------------------------------------------------52

Supplementary Figure 8. Complementary sensitivity analyses of the association between sexual factors and cardiovascular diseases after removing the SNPs associated with confounders or CVDs.------------------------------------------------------------------------------------------------------------------------------------------------------53

[Supplementary Figure 9. MR Leave one out analyses for age at first sexual intercourse on coronary artery disease. 5](#_Toc26192)4

[Supplementary Figure 10. MR Leave one out analyses for age at first sexual intercourse on myocardial infarction. 5](#_Toc21153)5

Supplementary Figure 11. MR Leave one out analyses for age at first sexual intercourse on atrial fibrillation.------------------------------------------------------------------56

Supplementary Figure 12. MR Leave one out analyses for age at first sexual intercourse on heart failure.-----------------------------------------------------------------------57

Supplementary Figure 13. MR Leave one out analyses for age at first sexual intercourse on ischemic stroke.-------------------------------------------------------------------58

[Supplementary Figure 14. MR Leave one out analyses for lifetime number of sexual partners on coronary artery disease. 5](#_Toc16019)9

[Supplementary Figure 15. MR Leave one out analyses for lifetime number of sexual partners on myocardial infarction. 6](#_Toc12731)0

[Supplementary Figure 16. MR Leave one out analyses for lifetime number of sexual partners on atrial fibrillation. 6](#_Toc5694)1

[Supplementary Figure 17. MR Leave one out analyses for lifetime number of sexual partners on heart failure. 6](#_Toc17905)2

[Supplementary Figure 18. MR Leave one out analyses for lifetime number of sexual partners on ischemic stroke. 6](#_Toc6549)3

[Supplementary Figure 19. MR Leave one out analyses for age at first sexual intercourse on coronary artery disease after removing SNPs associated with confounders or CVDs. 6](#_Toc18211)4

[Supplementary Figure 20. MR Leave one out analyses for age at first sexual intercourse on myocardial infarction after removing SNPs associated with confounders or CVDs. 6](#_Toc4855)5

[Supplementary Figure 21. MR Leave one out analyses for age at first sexual intercourse on atrial fibrillation after removing SNPs associated with confounders or CVDs. 6](#_Toc2092)6

[Supplementary Figure 22. MR Leave one out analyses for age at first sexual intercourse on heart failure after removing SNPs associated with confounders or CVDs. 6](#_Toc23531)7

[Supplementary Figure 23. MR Leave one out analyses for age at first sexual intercourse on ischemic stroke after removing SNPs associated with confounders or CVDs. 6](#_Toc20036)8

[Supplementary Figure 24. MR Leave one out analyses for lifetime number of sexual partners on coronary artery disease after removing SNPs associated with confounders or CVDs. 6](#_Toc22378)9

[Supplementary Figure 25. MR Leave one out analyses for lifetime number of sexual partners on myocardial infarction after removing SNPs associated with confounders or CVDs. 7](#_Toc18331)0

[Supplementary Figure 26. MR Leave one out analyses for lifetime number of sexual partners on atrial fibrillation after removing SNPs associated with confounders or CVDs. 7](#_Toc8803)1

[Supplementary Figure 27. MR Leave one out analyses for lifetime number of sexual partners on heart failure after removing SNPs associated with confounders or CVDs. 7](#_Toc20447)2

[Supplementary Figure 28. MR Leave one out analyses for lifetime number of sexual partners on ischemic stroke after removing SNPs associated with confounders or CVDs. 7](#_Toc25457)3

#

#

# Supplementary Table 1. Details of the studies and datasets included in the analyses.

| **Outcome traits** | **Data sources** | **Participants** | **Use in this MR study** | **Ancestry** | **Download link** |
| --- | --- | --- | --- | --- | --- |
| Age at first sexual intercourse | UKbiobank^1,2^ | 397,338 individuals | Exposure | European | https://gwas.mrcieu.ac.uk/datasets/ebi-a-GCST90000047/ |
| Lifetime number of sexual | UKbiobank^2,3^ | 378,882 individuals | Exposure | European | https://gwas.mrcieu.ac.uk/datasets/ukb-b-6591/ |
| Coronary artery disease | CARDIoGRAMplusC4D^4^ | 60,801 cases  123,504 controls | Outcome | Multi-ancestry  (77% European) | https://cvd.hugeamp.org/dinspector.html?dataset=GWAS_UKBiobankCARDIoGRAM_CARDIoGRAM |
| Coronary artery disease | FinnGen^5^ | 21,012 cases  197,780 controls | Outcome | European | https://www.finngen.fi/en |
| Myocardial infarction | CARDIoGRAMplusC4D^4^ | 43,676 cases  128,199 controls | Outcome | Multi-ancestry  (77% European) | https://cvd.hugeamp.org/dinspector.html?dataset=GWAS_UKBiobankCARDIoGRAM_CARDIoGRAM |
| Myocardial infarction | FinnGen^5^ | 12,801 cases  187,840 controls | Outcome | European | https://www.finngen.fi/en |
| Atrial fibrillation | Nielsen et al^6^ | 60,620 cases  970,216 controls | Outcome | European | http://csg.sph.umich.edu/willer/public/afib2018 |
| Atrial fibrillation | FinnGen^5^ | 22,068 cases  116,926 controls | Outcome | European | https://www.finngen.fi/en |
| Heart failure | Shah et al7 | 47,309 cases  930,014 controls | Outcome | European | https://cvd.hugeamp.org/dinspector.html?dataset=GWAS_HERMES_eu |
| Heart failure | FinnGen^5^ | 23,397 cases  194,811 controls | Outcome | European | https://www.finngen.fi/en |
| Ischemic stroke | MEGASTROKE^8^ | 34,217 cases  406,111 controls | Outcome | European | http://www.megastroke.org |
| Ischemic stroke | FinnGen^5^ | 10,551 cases  202,223 controls | Outcome | European | https://www.finngen.fi/en |

CARDIoGRAMplusC4D, Coronary ARtery DIsease Genome-wide Replication and Meta-analysis (CARDIoGRAM) plus The Coronary Artery Disease (C4D) Genetics.

**References**

1.Mills MC, Tropf FC, Brazel DM, van Zuydam N, Vaez A; eQTLGen Consortium; BIOS Consortium; Human Reproductive Behaviour Consortium; Pers TH, Snieder H, Perry JRB, Ong KK, den Hoed M, Barban N, Day FR. Identification of 371 genetic variants for age at first sex and birth linked to externalising behaviour. Nat Hum Behav. 2021 Dec;5(12):1717-1730.

2.Sudlow C, Gallacher J, Allen N et al (2015) UK Biobank: an open access resource for identifying the causes of a wide range of complex diseases of middle and old age. PLoS Med 12(3):e1001779.https://doi.org/10.1371/journal.pmed.1001779

3.The Neale Lab (2018) GWAS results. Available from http://www.nealelab.is/uk-biobank/.[ Accessed May 8,2023].

4.Nikpay M, Goel A, Won HH, Hall LM, Willenborg C, Kanoni S, et al. A comprehensive 1,000 Genomes-based genome-wide association meta-analysis of coronary artery disease. Nat Genet. 2015;47(10):1121-30.

5.FinnGen Consortium. FinnGen Data Freeze 5 (2021). Available at: https://www.finngen.fi/. [Accessed May 10, 2023].

6.Nielsen JB, Thorolfsdottir RB, Fritsche LG, Zhou W, Skov MW, Graham SE, et al. Biobank-driven genomic discovery yields new insight into atrial fibrillation biology. Nat Genet. 2018;50(9):1234-9.

7.Shah S, Henry A, Roselli C, Lin H, Sveinbjörnsson G, Fatemifar G, et al. Genome-wide association and Mendelian randomisation analysis provide insights into the pathogenesis of heart failure. Nat Commun. 2020;11(1):163.

8.Malik R, Chauhan G, Traylor M, Sargurupremraj M, Okada Y, Mishra A, et al. Multiancestry genome-wide association study of 520,000 subjects identifies 32 loci associated with stroke and stroke subtypes. Nat Genet. 2018;50(4):524-37.

# Supplementary Table 2. Genome-wide significant SNPs for age at first sexual intercourse.

| **SNP** | **CHR** | **Position** | **EA** | **OA** | **EAF** | **BETA** | **SE** | **P-value** | **N** | **F statistic** | **R^2^** |
| --- | --- | --- | --- | --- | --- | --- | --- | --- | --- | --- | --- |
| rs10104523 | 8 | 73889570 | C | T | 0.522885 | -0.0123 | 0.0021 | 6.70E-10 | 397338 | 34.30594977 | 8.63324E-05 |
| rs10144067 | 14 | 93885198 | T | C | 0.591843 | -0.0131 | 0.0021 | 1.60E-09 | 397338 | 38.91363633 | 9.79268E-05 |
| rs10469020 | 18 | 50811573 | T | A | 0.0822453 | 0.0217 | 0.0038 | 1.50E-08 | 397338 | 32.60994666 | 8.20647E-05 |
| rs10516875 | 4 | 91590266 | G | T | 0.234262 | -0.0148 | 0.0024 | 6.60E-10 | 397338 | 38.02758637 | 9.56972E-05 |
| rs10746578 | 9 | 81512820 | G | A | 0.339971 | 0.0129 | 0.0022 | 5.40E-09 | 397338 | 34.38205834 | 8.6524E-05 |
| rs10749233 | 10 | 118777998 | C | G | 0.759561 | 0.0175 | 0.0024 | 9.20E-13 | 397338 | 53.16813515 | 0.000133794 |
| rs10853981 | 19 | 4965064 | A | G | 0.331114 | -0.0123 | 0.0022 | 1.00E-08 | 397338 | 31.25810712 | 7.8663E-05 |
| rs10858054 | 1 | 115338190 | T | G | 0.184557 | 0.0149 | 0.0027 | 2.30E-08 | 397338 | 30.45389335 | 7.66393E-05 |
| rs10871582 | 18 | 53276589 | T | G | 0.340797 | -0.0153 | 0.0022 | 1.30E-12 | 397338 | 48.36545903 | 0.00012171 |
| rs10880086 | 12 | 41905017 | A | G | 0.217782 | 0.016 | 0.0025 | 1.30E-10 | 397338 | 40.95979383 | 0.000103075 |
| rs10922907 | 1 | 91193049 | T | A | 0.549617 | 0.0211 | 0.0021 | 2.10E-24 | 397338 | 100.9541404 | 0.000254013 |
| rs10955084 | 8 | 97825208 | T | C | 0.579108 | -0.0131 | 0.0021 | 2.50E-10 | 397338 | 38.91363633 | 9.79268E-05 |
| rs10978435 | 9 | 108949159 | C | T | 0.315156 | -0.0151 | 0.0022 | 1.20E-11 | 397338 | 47.10926701 | 0.000118549 |
| rs10992812 | 9 | 96392182 | A | G | 0.371303 | 0.0121 | 0.0022 | 1.70E-08 | 397338 | 30.24984774 | 7.61259E-05 |
| rs11240331 | 1 | 204968339 | T | C | 0.262918 | 0.014 | 0.0024 | 2.20E-09 | 397338 | 34.0276065 | 8.5632E-05 |
| rs112523595 | 3 | 85650341 | C | T | 0.630022 | 0.024 | 0.0022 | 1.20E-28 | 397338 | 119.0076654 | 0.000299424 |
| rs112633616 | 12 | 23238326 | C | A | 0.0335803 | 0.0386 | 0.0058 | 5.20E-11 | 397338 | 44.29109692 | 0.000111458 |
| rs112880127 | 10 | 120007757 | G | A | 0.166801 | 0.0151 | 0.0028 | 2.70E-08 | 397338 | 29.08276177 | 7.3189E-05 |
| rs113247159 | 7 | 140182514 | C | T | 0.289521 | 0.0156 | 0.0024 | 1.10E-10 | 397338 | 42.24978733 | 0.000106321 |
| rs113338260 | 5 | 46004640 | C | T | 0.216003 | -0.0149 | 0.0025 | 2.20E-09 | 397338 | 35.5214212 | 8.9391E-05 |
| rs1156981 | 1 | 88829969 | G | A | 0.9012788 | -0.0207 | 0.0035 | 1.60E-09 | 397338 | 34.97859944 | 8.8025E-05 |
| rs11678980 | 2 | 162101261 | A | G | 0.464157 | -0.012 | 0.0021 | 1.20E-08 | 397338 | 32.65289687 | 8.21728E-05 |
| rs11688027 | 2 | 78018164 | G | A | 0.0739063 | 0.0224 | 0.004 | 1.40E-08 | 397338 | 31.35984215 | 7.8919E-05 |
| rs11729080 | 4 | 112503872 | A | G | 0.173686 | 0.0222 | 0.0027 | 4.10E-16 | 397338 | 67.60459798 | 0.000170116 |
| rs117831144 | 20 | 14731057 | T | C | 0.0292987 | -0.0356 | 0.0064 | 3.40E-08 | 397338 | 30.94125051 | 7.78657E-05 |
| rs11866420 | 16 | 90054704 | G | C | 0.572075 | -0.0168 | 0.0021 | 7.60E-16 | 397338 | 63.99967786 | 0.000161046 |
| rs12049116 | 1 | 151050283 | T | C | 0.780985 | 0.0151 | 0.0025 | 1.90E-09 | 397338 | 36.48141637 | 9.18066E-05 |
| rs12147463 | 14 | 41059928 | A | G | 0.196741 | -0.0202 | 0.0026 | 3.60E-14 | 397338 | 60.36064292 | 0.00015189 |
| rs12203592 | 6 | 396321 | T | C | 0.116 | 0.014 | 0.0026 | 3.60E-08 | 397338 | 28.9939369 | 7.29655E-05 |
| rs12204714 | 6 | 152235339 | T | C | 0.631766 | 0.0275 | 0.0021 | 8.30E-38 | 397338 | 171.4843976 | 0.000431399 |
| rs12244388 | 10 | 104640052 | A | G | 0.336281 | -0.0136 | 0.0022 | 5.80E-10 | 397338 | 38.21468368 | 9.6168E-05 |
| rs1226414 | 2 | 157109930 | T | A | 0.507366 | 0.0144 | 0.0021 | 2.30E-12 | 397338 | 47.02017149 | 0.000118325 |
| rs12292980 | 11 | 27734349 | A | G | 0.319211 | -0.0127 | 0.0022 | 1.10E-08 | 397338 | 33.32421243 | 8.38621E-05 |
| rs12376530 | 9 | 14529836 | C | A | 0.260881 | -0.0145 | 0.0024 | 7.90E-10 | 397338 | 36.50155238 | 9.18573E-05 |
| rs12448731 | 16 | 49622284 | T | C | 0.140784 | -0.0179 | 0.003 | 2.10E-09 | 397338 | 35.60093191 | 8.9591E-05 |
| rs12463727 | 2 | 26948413 | A | G | 0.526876 | 0.0141 | 0.0021 | 5.90E-12 | 397338 | 45.08140573 | 0.000113446 |
| rs12511982 | 4 | 60736871 | A | G | 0.552942 | 0.0115 | 0.0021 | 1.60E-08 | 397338 | 29.98851118 | 7.54682E-05 |
| rs12523097 | 5 | 167411400 | C | T | 0.296492 | -0.0138 | 0.0023 | 1.20E-09 | 397338 | 35.99981879 | 9.05948E-05 |
| rs12523398 | 5 | 45119647 | A | T | 0.17254 | 0.0219 | 0.0027 | 1.10E-15 | 397338 | 65.7897923 | 0.00016555 |
| rs12554512 | 9 | 23352293 | C | T | 0.41749 | 0.0126 | 0.0021 | 1.20E-09 | 397338 | 35.99981879 | 9.05948E-05 |
| rs12653396 | 5 | 87847273 | A | T | 0.568347 | -0.0199 | 0.0021 | 2.00E-21 | 397338 | 89.79773394 | 0.000225948 |
| rs12714592 | 3 | 84387950 | C | A | 0.273887 | -0.0217 | 0.0023 | 1.10E-20 | 397338 | 89.01467482 | 0.000223979 |
| rs12714702 | 3 | 88249922 | G | A | 0.841317 | -0.0242 | 0.0028 | 1.40E-17 | 397338 | 74.69860359 | 0.000187963 |
| rs12757779 | 1 | 232581436 | A | G | 0.227918 | 0.0134 | 0.0025 | 3.70E-08 | 397338 | 28.72945539 | 7.23E-05 |
| rs12878359 | 14 | 58816212 | C | A | 0.544224 | 0.0134 | 0.0021 | 5.30E-10 | 397338 | 40.71634834 | 0.000102463 |
| rs12907546 | 15 | 47684280 | A | G | 0.211577 | -0.0227 | 0.0025 | 2.10E-19 | 397338 | 82.44598501 | 0.000207454 |
| rs13175535 | 5 | 31078814 | A | G | 0.293425 | 0.0125 | 0.0023 | 4.40E-08 | 397338 | 29.53671333 | 7.43313E-05 |
| rs13178956 | 5 | 154786457 | T | A | 0.256688 | -0.0164 | 0.0024 | 4.20E-12 | 397338 | 46.69420941 | 0.000117504 |
| rs1320138 | 2 | 144158287 | C | T | 0.577227 | 0.0148 | 0.0021 | 1.90E-12 | 397338 | 49.66868423 | 0.000124989 |
| rs1320330 | 2 | 622225 | G | T | 0.827746 | -0.0178 | 0.0027 | 1.10E-10 | 397338 | 43.46205832 | 0.000109372 |
| rs13280592 | 8 | 116686752 | G | C | 0.726064 | -0.0134 | 0.0023 | 9.30E-09 | 397338 | 33.94311837 | 8.54194E-05 |
| rs13307225 | 7 | 105084671 | A | G | 0.892437 | 0.0218 | 0.0034 | 4.70E-11 | 397338 | 41.11051971 | 0.000103455 |
| rs13394795 | 2 | 202867731 | C | T | 0.241066 | 0.0134 | 0.0024 | 2.90E-08 | 397338 | 31.1734542 | 7.845E-05 |
| rs1368546 | 2 | 104057364 | C | T | 0.556758 | 0.0165 | 0.0021 | 2.50E-15 | 397338 | 61.73438314 | 0.000155347 |
| rs1392816 | 1 | 66481188 | T | C | 0.387156 | 0.017 | 0.0021 | 6.20E-16 | 397338 | 65.53254996 | 0.000164903 |
| rs141547796 | 6 | 50615935 | A | G | 0.0827233 | 0.0324 | 0.0038 | 1.40E-17 | 397338 | 72.69769502 | 0.000182929 |
| rs147633738 | 1 | 156320524 | T | C | 0.0664712 | -0.0242 | 0.0042 | 8.10E-09 | 397338 | 33.19937938 | 8.35479E-05 |
| rs147725178 | 2 | 185529344 | T | C | 0.0403177 | -0.0307 | 0.0054 | 5.70E-09 | 397338 | 32.3211679 | 8.13381E-05 |
| rs1547351 | 8 | 36842153 | A | T | 0.413023 | 0.0123 | 0.0021 | 2.00E-08 | 397338 | 34.30594977 | 8.63324E-05 |
| rs1585634 | 8 | 54396376 | C | G | 0.800065 | -0.0146 | 0.0026 | 1.30E-08 | 397338 | 31.53238566 | 7.93532E-05 |
| rs1609598 | 20 | 51510926 | T | C | 0.349428 | -0.0122 | 0.0022 | 3.30E-08 | 397338 | 30.75191133 | 7.73892E-05 |
| rs1866710 | 11 | 12875312 | G | A | 0.707985 | 0.0148 | 0.0023 | 2.30E-10 | 397338 | 41.4062188 | 0.000104199 |
| rs186723454 | 3 | 54156598 | G | A | 0.151927 | -0.0183 | 0.0029 | 5.10E-11 | 397338 | 39.82025141 | 0.000100208 |
| rs191242857 | 9 | 127870796 | A | G | 0.313628 | 0.0125 | 0.0022 | 1.40E-08 | 397338 | 32.28289535 | 8.12418E-05 |
| rs1925686 | 6 | 87858691 | A | G | 0.389821 | 0.0123 | 0.0021 | 7.30E-09 | 397338 | 34.30594977 | 8.63324E-05 |
| rs1931263 | 1 | 96175101 | T | G | 0.489975 | -0.0117 | 0.0021 | 1.70E-08 | 397338 | 31.04066008 | 7.81158E-05 |
| rs1962545 | 1 | 7522336 | C | T | 0.519546 | -0.0124 | 0.0021 | 4.20E-10 | 397338 | 34.86603765 | 8.77418E-05 |
| rs1976423 | 5 | 104042643 | C | A | 0.495785 | -0.0114 | 0.0021 | 4.00E-08 | 397338 | 29.46923942 | 7.41616E-05 |
| rs1991651 | 8 | 10706411 | G | C | 0.618315 | 0.0182 | 0.0021 | 1.70E-17 | 397338 | 75.11073304 | 0.000189 |
| rs1995181 | 12 | 24195048 | A | T | 0.468302 | -0.0121 | 0.0021 | 4.50E-09 | 397338 | 33.19937938 | 8.35479E-05 |
| rs2084572 | 3 | 17315758 | G | A | 0.450968 | 0.014 | 0.0021 | 3.50E-11 | 397338 | 44.44422073 | 0.000111843 |
| rs2091377 | 2 | 6145158 | T | C | 0.37198 | 0.0118 | 0.0021 | 3.10E-08 | 397338 | 31.57353722 | 7.94568E-05 |
| rs2093623 | 10 | 10922977 | A | G | 0.497871 | 0.0139 | 0.0021 | 4.50E-11 | 397338 | 43.81157086 | 0.000110251 |
| rs2130894 | 10 | 134039242 | T | C | 0.283965 | 0.0131 | 0.0024 | 1.70E-08 | 397338 | 29.79325281 | 7.49769E-05 |
| rs215639 | 7 | 32373639 | T | C | 0.654179 | 0.0131 | 0.0022 | 2.30E-09 | 397338 | 35.4564331 | 8.92274E-05 |
| rs2174752 | 13 | 69332015 | T | G | 0.452931 | -0.0128 | 0.0021 | 5.90E-10 | 397338 | 37.15174043 | 9.34933E-05 |
| rs2176337 | 9 | 108959330 | T | A | 0.314601 | -0.0151 | 0.0022 | 1.10E-11 | 397338 | 47.10926701 | 0.000118549 |
| rs2188151 | 3 | 50201924 | T | G | 0.425033 | -0.021 | 0.0021 | 2.10E-24 | 397338 | 99.99949665 | 0.000251612 |
| rs222440 | 6 | 52946320 | C | T | 0.819363 | 0.017 | 0.0027 | 4.90E-10 | 397338 | 39.64314751 | 9.97624E-05 |
| rs2274568 | 1 | 110612925 | A | G | 0.58113 | -0.0129 | 0.0021 | 4.60E-10 | 397338 | 37.73450394 | 9.49597E-05 |
| rs2279574 | 12 | 89745477 | A | C | 0.542992 | 0.0119 | 0.0021 | 2.60E-08 | 397338 | 32.11094948 | 8.08091E-05 |
| rs2406374 | 5 | 106936435 | T | C | 0.314036 | 0.0139 | 0.0022 | 4.30E-10 | 397338 | 39.91922055 | 0.000100457 |
| rs2612030 | 3 | 53773437 | C | T | 0.838336 | 0.024 | 0.0028 | 1.20E-17 | 397338 | 73.46901795 | 0.00018487 |
| rs2650705 | 10 | 63239803 | G | A | 0.838109 | -0.0166 | 0.0028 | 3.70E-09 | 397338 | 35.14778227 | 8.84508E-05 |
| rs28406364 | 17 | 47454507 | T | C | 0.376997 | -0.0165 | 0.0021 | 8.10E-15 | 397338 | 61.73438314 | 0.000155347 |
| rs28929474 | 14 | 94844947 | T | C | 0.020291 | 0.0416 | 0.0074 | 1.20E-08 | 397338 | 31.60247058 | 7.95296E-05 |
| rs2974311 | 8 | 42455166 | A | G | 0.498484 | 0.0131 | 0.0021 | 2.70E-10 | 397338 | 38.91363633 | 9.79268E-05 |
| rs3007104 | 14 | 47367434 | A | G | 0.42241 | -0.0148 | 0.0021 | 1.50E-12 | 397338 | 49.66868423 | 0.000124989 |
| rs341521 | 13 | 60399045 | A | G | 0.701785 | -0.0159 | 0.0023 | 9.00E-13 | 397338 | 47.78992958 | 0.000120261 |
| rs34155040 | 18 | 44797697 | T | C | 0.423567 | 0.0142 | 0.0021 | 1.80E-11 | 397338 | 45.72312586 | 0.000115061 |
| rs3447 | 9 | 86327243 | G | C | 0.150353 | 0.0177 | 0.0029 | 3.70E-09 | 397338 | 37.25189335 | 9.37453E-05 |
| rs34517439 | 1 | 78450517 | A | C | 0.12559 | -0.0195 | 0.0032 | 8.20E-10 | 397338 | 37.13360215 | 9.34477E-05 |
| rs34606772 | 3 | 24908376 | T | C | 0.419586 | -0.0118 | 0.0021 | 1.60E-08 | 397338 | 31.57353722 | 7.94568E-05 |
| rs34804222 | 11 | 43771084 | G | A | 0.4061 | 0.0151 | 0.0021 | 1.90E-13 | 397338 | 51.7026876 | 0.000130106 |
| rs34811474 | 4 | 25408838 | A | G | 0.232008 | 0.0155 | 0.0024 | 1.30E-10 | 397338 | 41.7098595 | 0.000104963 |
| rs35077383 | 1 | 151754112 | C | T | 0.520771 | -0.011 | 0.0021 | 3.90E-08 | 397338 | 27.43750362 | 6.90489E-05 |
| rs35851551 | 7 | 31330785 | G | A | 0.101865 | -0.0247 | 0.0034 | 2.50E-13 | 397338 | 52.77568591 | 0.000132806 |
| rs359271 | 2 | 60463149 | C | T | 0.579878 | -0.0181 | 0.0021 | 4.10E-18 | 397338 | 74.28760793 | 0.000186929 |
| rs3866460 | 6 | 152139943 | C | T | 0.105261 | 0.019 | 0.0034 | 1.70E-08 | 397338 | 31.22821651 | 7.85878E-05 |
| rs3896224 | 10 | 106467853 | G | A | 0.411417 | 0.0198 | 0.0021 | 2.70E-21 | 397338 | 88.89751172 | 0.000223684 |
| rs410520 | 17 | 4938924 | T | C | 0.554281 | 0.0113 | 0.0021 | 4.60E-08 | 397338 | 28.95450278 | 7.28663E-05 |
| rs4246175 | 9 | 134930808 | A | T | 0.679399 | 0.0144 | 0.0022 | 4.20E-11 | 397338 | 42.84275956 | 0.000107813 |
| rs435538 | 5 | 24921398 | G | C | 0.231178 | -0.017 | 0.0024 | 2.50E-12 | 397338 | 50.17335856 | 0.000126258 |
| rs4439537 | 11 | 79887549 | C | T | 0.523977 | 0.0132 | 0.0021 | 1.10E-10 | 397338 | 39.51000521 | 9.94274E-05 |
| rs4557006 | 2 | 22443840 | A | G | 0.450612 | -0.0118 | 0.0021 | 8.30E-09 | 397338 | 31.57353722 | 7.94568E-05 |
| rs4569188 | 14 | 93915929 | A | G | 0.665948 | -0.0135 | 0.0022 | 1.30E-09 | 397338 | 37.65476914 | 9.47591E-05 |
| rs4602427 | 3 | 117474457 | G | C | 0.802398 | -0.0172 | 0.0026 | 2.30E-11 | 397338 | 43.76309333 | 0.000110129 |
| rs4702 | 15 | 91426560 | A | G | 0.556681 | 0.0172 | 0.0021 | 1.10E-16 | 397338 | 67.08356256 | 0.000168805 |
| rs4709807 | 6 | 164432799 | C | T | 0.758339 | -0.0138 | 0.0024 | 1.00E-08 | 397338 | 33.06233358 | 8.32031E-05 |
| rs4728298 | 7 | 133436768 | A | T | 0.184609 | -0.0219 | 0.0029 | 2.10E-14 | 397338 | 57.0282504 | 0.000143506 |
| rs4800204 | 18 | 22647270 | T | C | 0.567723 | -0.0125 | 0.0021 | 4.60E-09 | 397338 | 35.43066066 | 8.91626E-05 |
| rs4809230 | 20 | 62439274 | A | G | 0.0765392 | 0.0231 | 0.0039 | 2.50E-09 | 397338 | 35.08266365 | 8.82869E-05 |
| rs4868800 | 5 | 166985224 | T | G | 0.581603 | 0.0128 | 0.0021 | 8.20E-10 | 397338 | 37.15174043 | 9.34933E-05 |
| rs4952343 | 2 | 32858637 | G | A | 0.447987 | 0.0126 | 0.0021 | 2.20E-09 | 397338 | 35.99981879 | 9.05948E-05 |
| rs4961705 | 9 | 16347927 | C | G | 0.346584 | 0.013 | 0.0022 | 4.20E-09 | 397338 | 34.91717962 | 8.78705E-05 |
| rs56306056 | 2 | 184416610 | A | G | 0.214994 | 0.0157 | 0.0025 | 4.10E-10 | 397338 | 39.43820149 | 9.92467E-05 |
| rs56392241 | 3 | 131968209 | C | A | 0.392686 | -0.014 | 0.0021 | 8.90E-12 | 397338 | 44.44422073 | 0.000111843 |
| rs58938116 | 7 | 121946082 | T | G | 0.170073 | -0.0175 | 0.0028 | 1.60E-10 | 397338 | 39.06230338 | 9.83008E-05 |
| rs590414 | 11 | 105746052 | T | A | 0.502589 | 0.0135 | 0.0021 | 1.70E-10 | 397338 | 41.3263226 | 0.000103998 |
| rs592278 | 18 | 40231833 | A | G | 0.401211 | -0.0125 | 0.0021 | 3.40E-09 | 397338 | 35.43066066 | 8.91626E-05 |
| rs6058613 | 20 | 30864279 | G | C | 0.837431 | -0.0171 | 0.0028 | 1.80E-09 | 397338 | 37.29700614 | 9.38589E-05 |
| rs60775983 | 5 | 124204071 | G | A | 0.157411 | 0.0178 | 0.0029 | 4.50E-10 | 397338 | 37.67400775 | 9.48075E-05 |
| rs61856978 | 10 | 97941022 | C | T | 0.339736 | -0.0154 | 0.0022 | 5.70E-13 | 397338 | 48.99975336 | 0.000123305 |
| rs62134195 | 2 | 45062249 | T | C | 0.0417297 | 0.0318 | 0.0052 | 5.30E-10 | 397338 | 37.39774075 | 9.41123E-05 |
| rs62177795 | 2 | 63475640 | A | G | 0.219347 | 0.019 | 0.0025 | 9.00E-15 | 397338 | 57.75970927 | 0.000145346 |
| rs62439690 | 7 | 21417556 | A | G | 0.262838 | -0.0155 | 0.0024 | 7.20E-11 | 397338 | 41.7098595 | 0.000104963 |
| rs6504551 | 17 | 65903326 | G | T | 0.263558 | -0.0149 | 0.0023 | 1.10E-10 | 397338 | 41.96765265 | 0.000105611 |
| rs6517512 | 21 | 40512129 | G | A | 0.9550851 | -0.0275 | 0.005 | 3.20E-08 | 397338 | 30.24984774 | 7.61259E-05 |
| rs6564268 | 16 | 75606878 | G | C | 0.0544569 | 0.0266 | 0.0045 | 5.50E-09 | 397338 | 34.94105869 | 8.79306E-05 |
| rs6586405 | 1 | 234739101 | A | C | 0.328821 | -0.0122 | 0.0022 | 3.00E-08 | 397338 | 30.75191133 | 7.73892E-05 |
| rs6719762 | 2 | 60166832 | C | T | 0.473891 | -0.0221 | 0.0021 | 7.20E-27 | 397338 | 110.7500094 | 0.000278654 |
| rs6744794 | 2 | 44842145 | G | C | 0.621453 | -0.0191 | 0.0021 | 1.30E-19 | 397338 | 82.72293962 | 0.000208151 |
| rs6747099 | 2 | 60777498 | C | G | 0.473611 | 0.0123 | 0.0021 | 4.90E-09 | 397338 | 34.30594977 | 8.63324E-05 |
| rs6748341 | 2 | 225377574 | G | C | 0.314943 | 0.0149 | 0.0022 | 1.10E-11 | 397338 | 45.86960383 | 0.00011543 |
| rs67723420 | 3 | 35775115 | A | T | 0.375993 | 0.0146 | 0.0021 | 1.30E-11 | 397338 | 48.33535761 | 0.000121634 |
| rs6776937 | 3 | 60870307 | A | C | 0.21437 | -0.0138 | 0.0025 | 1.20E-08 | 397338 | 30.47024663 | 7.66805E-05 |
| rs6966769 | 7 | 1299334 | G | A | 0.113613 | 0.0182 | 0.0033 | 1.90E-08 | 397338 | 30.41674313 | 7.65458E-05 |
| rs6978112 | 7 | 1966841 | T | C | 0.411394 | -0.0158 | 0.0021 | 2.70E-14 | 397338 | 56.60742482 | 0.000142447 |
| rs7008955 | 8 | 26334103 | G | T | 0.527116 | 0.0116 | 0.0021 | 3.40E-08 | 397338 | 30.51231807 | 7.67863E-05 |
| rs702 | 4 | 28710551 | T | A | 0.837663 | 0.0181 | 0.0028 | 2.80E-10 | 397338 | 41.78677946 | 0.000105156 |
| rs705240 | 3 | 118457615 | T | C | 0.18545 | -0.015 | 0.0027 | 7.00E-09 | 397338 | 30.86404218 | 7.76714E-05 |
| rs7079070 | 10 | 134182921 | A | G | 0.453972 | -0.0165 | 0.0021 | 1.20E-15 | 397338 | 61.73438314 | 0.000155347 |
| rs7091634 | 10 | 9973015 | G | A | 0.386449 | 0.0125 | 0.0021 | 3.80E-09 | 397338 | 35.43066066 | 8.91626E-05 |
| rs7110863 | 11 | 112843138 | G | A | 0.385723 | -0.0154 | 0.0021 | 5.00E-13 | 397338 | 53.77750709 | 0.000135327 |
| rs714393 | 2 | 212698718 | T | C | 0.456707 | 0.0126 | 0.0021 | 1.50E-09 | 397338 | 35.99981879 | 9.05948E-05 |
| rs7151954 | 14 | 103323448 | G | A | 0.200334 | -0.0208 | 0.0026 | 4.00E-16 | 397338 | 63.99967786 | 0.000161046 |
| rs7152323 | 14 | 98553482 | G | A | 0.41531 | -0.0156 | 0.0021 | 3.10E-13 | 397338 | 55.1833957 | 0.000138864 |
| rs7167444 | 15 | 97495941 | T | G | 0.255667 | -0.0138 | 0.0024 | 8.10E-09 | 397338 | 33.06233358 | 8.32031E-05 |
| rs7188873 | 16 | 24727064 | G | A | 0.623896 | -0.0146 | 0.0021 | 5.80E-12 | 397338 | 48.33535761 | 0.000121634 |
| rs7201310 | 16 | 12513797 | A | C | 0.687501 | 0.0121 | 0.0023 | 2.70E-08 | 397338 | 27.67660927 | 6.96506E-05 |
| rs7236339 | 18 | 77579773 | A | G | 0.229726 | -0.0201 | 0.0025 | 3.90E-16 | 397338 | 64.64127463 | 0.00016266 |
| rs72674824 | 8 | 95489281 | C | T | 0.240772 | 0.0137 | 0.0024 | 3.40E-08 | 397338 | 32.58490543 | 8.20017E-05 |
| rs72887338 | 6 | 67536056 | C | T | 0.387313 | -0.0163 | 0.0021 | 1.60E-14 | 397338 | 60.24686228 | 0.000151604 |
| rs7381195 | 5 | 60030791 | A | T | 0.610705 | -0.0136 | 0.0021 | 1.60E-10 | 397338 | 41.94083197 | 0.000105544 |
| rs7452074 | 6 | 125050733 | C | A | 0.526613 | -0.0128 | 0.0021 | 4.90E-10 | 397338 | 37.15174043 | 9.34933E-05 |
| rs74737734 | 14 | 30726670 | T | A | 0.0271973 | 0.0353 | 0.0064 | 2.70E-08 | 397338 | 30.42196601 | 7.6559E-05 |
| rs7476 | 11 | 46342834 | C | A | 0.31008 | -0.014 | 0.0022 | 5.60E-10 | 397338 | 40.49566393 | 0.000101908 |
| rs7503604 | 17 | 79095629 | A | C | 0.512765 | 0.0129 | 0.0021 | 6.10E-10 | 397338 | 37.73450394 | 9.49597E-05 |
| rs7525548 | 1 | 75001474 | T | A | 0.563002 | 0.0145 | 0.0021 | 2.90E-12 | 397338 | 47.67549699 | 0.000119973 |
| rs7566527 | 2 | 100811970 | T | C | 0.401302 | 0.0137 | 0.0021 | 3.80E-11 | 397338 | 42.55987648 | 0.000107102 |
| rs7575189 | 2 | 174015168 | A | G | 0.590723 | 0.0164 | 0.0021 | 1.90E-15 | 397338 | 60.98835515 | 0.00015347 |
| rs7618715 | 3 | 70873278 | A | G | 0.419237 | 0.0126 | 0.0021 | 1.90E-09 | 397338 | 35.99981879 | 9.05948E-05 |
| rs763053 | 16 | 735921 | C | T | 0.224209 | 0.0163 | 0.0025 | 4.90E-11 | 397338 | 42.51018602 | 0.000106977 |
| rs76513770 | 16 | 72505534 | C | T | 0.129461 | 0.024 | 0.0031 | 4.90E-15 | 397338 | 59.93726334 | 0.000150825 |
| rs766406 | 6 | 26319588 | T | G | 0.633222 | 0.0135 | 0.0021 | 5.20E-10 | 397338 | 41.3263226 | 0.000103998 |
| rs7671317 | 4 | 62972597 | T | G | 0.680847 | 0.0122 | 0.0022 | 3.50E-08 | 397338 | 30.75191133 | 7.73892E-05 |
| rs767943 | 6 | 23446691 | A | C | 0.261794 | -0.0171 | 0.0024 | 5.70E-13 | 397338 | 50.76536947 | 0.000127748 |
| rs7704530 | 5 | 30845465 | A | G | 0.731967 | -0.0128 | 0.0023 | 2.90E-08 | 397338 | 30.97148872 | 7.79418E-05 |
| rs7783012 | 7 | 114116881 | A | G | 0.593585 | -0.0196 | 0.0021 | 1.40E-20 | 397338 | 87.11067264 | 0.000219189 |
| rs7785195 | 7 | 3424686 | A | G | 0.659316 | 0.0142 | 0.0022 | 9.50E-11 | 397338 | 41.66094732 | 0.00010484 |
| rs7804551 | 7 | 99119110 | G | A | 0.153971 | 0.0226 | 0.0029 | 2.90E-15 | 397338 | 60.73215566 | 0.000152825 |
| rs7815125 | 8 | 87680112 | A | T | 0.825198 | 0.0179 | 0.0027 | 3.30E-11 | 397338 | 43.95176779 | 0.000110604 |
| rs7824756 | 8 | 51118559 | C | T | 0.292347 | -0.0155 | 0.0023 | 2.80E-11 | 397338 | 45.41565042 | 0.000114287 |
| rs783544 | 15 | 83240293 | C | A | 0.749059 | 0.0148 | 0.0024 | 3.50E-10 | 397338 | 38.02758637 | 9.56972E-05 |
| rs7909331 | 10 | 11205224 | G | A | 0.163948 | -0.0156 | 0.0028 | 2.90E-08 | 397338 | 31.04066008 | 7.81158E-05 |
| rs79269403 | 3 | 108036819 | A | G | 0.231055 | 0.0206 | 0.0025 | 3.10E-17 | 397338 | 67.89725824 | 0.000170852 |
| rs7927195 | 11 | 127478073 | G | A | 0.612168 | -0.0152 | 0.0021 | 4.90E-13 | 397338 | 52.38975897 | 0.000131835 |
| rs7942078 | 11 | 28656064 | T | A | 0.343914 | 0.0161 | 0.0022 | 6.60E-14 | 397338 | 53.55551555 | 0.000134768 |
| rs794375 | 7 | 75147801 | C | T | 0.426216 | 0.0142 | 0.0021 | 1.40E-11 | 397338 | 45.72312586 | 0.000115061 |
| rs7955865 | 12 | 56468706 | T | A | 0.64771 | -0.0134 | 0.0022 | 8.40E-10 | 397338 | 37.09898682 | 9.33606E-05 |
| rs7972441 | 12 | 84043146 | A | C | 0.642439 | -0.0149 | 0.0022 | 1.10E-11 | 397338 | 45.86960383 | 0.00011543 |
| rs803679 | 1 | 44349405 | A | G | 0.795577 | 0.0175 | 0.0025 | 6.80E-12 | 397338 | 48.99975336 | 0.000123305 |
| rs807478 | 19 | 36252494 | G | A | 0.4959 | 0.0122 | 0.0021 | 1.90E-09 | 397338 | 33.75039701 | 8.49345E-05 |
| rs809955 | 4 | 140874760 | A | G | 0.36658 | 0.0157 | 0.0021 | 1.00E-13 | 397338 | 55.8931427 | 0.00014065 |
| rs8180995 | 8 | 143326237 | G | A | 0.464496 | 0.0122 | 0.0021 | 8.90E-10 | 397338 | 33.75039701 | 8.49345E-05 |
| rs838042 | 2 | 140320186 | A | G | 0.68737 | 0.0162 | 0.0022 | 3.90E-13 | 397338 | 54.22286756 | 0.000136447 |
| rs9403187 | 6 | 100324813 | T | A | 0.352619 | 0.0149 | 0.0022 | 1.70E-11 | 397338 | 45.86960383 | 0.00011543 |
| rs9514600 | 13 | 107644422 | G | C | 0.505103 | -0.0114 | 0.0021 | 2.40E-08 | 397338 | 29.46923942 | 7.41616E-05 |
| rs9536994 | 13 | 55757388 | C | A | 0.352549 | 0.0125 | 0.0022 | 4.20E-09 | 397338 | 32.28289535 | 8.12418E-05 |
| rs9538248 | 13 | 59492828 | A | C | 0.321712 | -0.0159 | 0.0022 | 3.00E-13 | 397338 | 52.23320816 | 0.000131441 |
| rs9581878 | 13 | 28104552 | A | T | 0.140178 | 0.0199 | 0.003 | 1.50E-10 | 397338 | 44.00088963 | 0.000110727 |
| rs9643087 | 8 | 115382484 | T | C | 0.525442 | -0.0135 | 0.0021 | 9.40E-11 | 397338 | 41.3263226 | 0.000103998 |
| rs9809849 | 3 | 3726156 | A | G | 0.425291 | -0.014 | 0.0021 | 1.80E-11 | 397338 | 44.44422073 | 0.000111843 |
| rs9835772 | 3 | 85766025 | T | A | 0.242586 | -0.0138 | 0.0024 | 9.90E-09 | 397338 | 33.06233358 | 8.32031E-05 |
| rs9923553 | 16 | 5825579 | G | A | 0.291349 | -0.0139 | 0.0023 | 3.80E-10 | 397338 | 36.52344565 | 9.19124E-05 |
| rs993700 | 4 | 67825894 | C | T | 0.777798 | 0.0157 | 0.0025 | 2.90E-10 | 397338 | 39.43820149 | 9.92467E-05 |

SNPs, Single-nucleotide polymorphiss. CHR, chromosome. EA, effect allele.EAF, effect allele frequency. SE, standard error. R², explained variation by SNPs.

# Supplementary Table 3. Association of genome-wide significant SNPs for age at first sexual intercourse with cardiovascular diseases.

| **Age at first sexual intercourse** | | | | **Coronary artery disease** | | | | **Myocardial infarction** | | | | **Atrial fibrillation** | | | | **Heart failure** | | | | **Ischemic stroke** | | | |
| --- | --- | --- | --- | --- | --- | --- | --- | --- | --- | --- | --- | --- | --- | --- | --- | --- | --- | --- | --- | --- | --- | --- | --- |
| **Data sources: UKbiobank** | | | | **CARDIoGRAMplusC4D** | | **FinnGen** | | **CARDIoGRAMplusC4D** | | **FinnGen** | | **Nielsen et al** | | **FinnGen** | | **HERMES** | | **FinnGen** | | **MEGASTROKE** | | **FinnGen** | |
| SNP | EA | BETA | SE | BETA | SE | BETA | SE | BETA | SE | BETA | SE | BETA | SE | BETA | SE | BETA | SE | BETA | SE | BETA | SE | BETA | SE |
| rs10104523 | C | -0.012 | 0.002 | 0 | 0.009 | -0.003 | 0.013 | 0.006 | 0.01 | 0.004 | 0.016 | 0.014 | 0.007 | 0.02 | 0.013 | 0.007 | 0.008 | 0.023 | 0.011 | 0.008 | 0.010 | 0.034 | 0.015 |
| rs10144067 | T | -0.013 | 0.002 |  |  | 0.007 | 0.013 |  |  | 0.019 | 0.016 | -0.002 | 0.007 | 0.002 | 0.013 | 0.003 | 0.009 | 0.004 | 0.011 | -0.002 | 0.011 | -0.012 | 0.015 |
| rs10469020 | T | 0.022 | 0.004 |  |  |  |  |  |  |  |  |  |  |  |  |  |  |  |  |  |  |  |  |
| rs10516875 | G | -0.015 | 0.002 | 0.018 | 0.011 | 0.001 | 0.016 | 0.010 | 0.012 | -0.003 | 0.019 | -0.004 | 0.008 | -0.014 | 0.015 | -0.007 | 0.009 | -0.012 | 0.014 | 0.019 | 0.01 | 0.007 | 0.018 |
| rs10746578 | G | 0.013 | 0.002 | -0.014 | 0.01 | -0.023 | 0.014 | -0.012 | 0.011 | -0.017 | 0.017 | 0.005 | 0.007 | -0.012 | 0.013 | 0.007 | 0.008 | -0.02 | 0.012 | 0.002 | 0.01 | -0.002 | 0.016 |
| rs10749233 | C | 0.018 | 0.002 |  |  |  |  |  |  |  |  |  |  |  |  |  |  |  |  |  |  |  |  |
| rs10853981 | A | -0.012 | 0.002 | 0.028 | 0.01 | -0.003 | 0.015 | 0.023 | 0.011 | -0.005 | 0.018 | 0.02 | 0.007 | -0.003 | 0.014 | 0.007 | 0.008 | -0.008 | 0.013 | 0.016 | 0.013 | -0.015 | 0.017 |
| rs10858054 | T | 0.015 | 0.003 | -0.005 | 0.012 | 0.02 | 0.017 | 0.002 | 0.013 | 0.014 | 0.021 | 0.002 | 0.009 | 0.025 | 0.017 | 0.003 | 0.01 | -0.016 | 0.015 | -0.016 | 0.012 | 0.029 | 0.02 |
| rs10871582 | T | -0.015 | 0.002 | 0.008 | 0.01 | -0.003 | 0.014 | 0.006 | 0.011 | -0.01 | 0.016 | 0.001 | 0.007 | -0.013 | 0.013 | 0.006 | 0.008 | -0.001 | 0.012 | 0.009 | 0.01 | 0.008 | 0.016 |
| rs10880086 | A | 0.016 | 0.003 | 0.022 | 0.012 | -0.028 | 0.016 | 0.028 | 0.013 | -0.047 | 0.019 | 0.001 | 0.008 | 0.034 | 0.015 | 0.01 | 0.009 | 0.005 | 0.014 | 0.069 | 0.038 | 0.006 | 0.019 |
| rs10922907 | T | 0.021 | 0.002 |  |  |  |  |  |  |  |  |  |  |  |  |  |  |  |  |  |  |  |  |
| rs10955084 | T | -0.013 | 0.002 | -0.004 | 0.01 | 0.025 | 0.013 | -0.01 | 0.011 | 0.022 | 0.016 | -0.013 | 0.007 | -0.016 | 0.013 | -0.001 | 0.008 | -0.006 | 0.011 | 0.012 | 0.01 | 0.004 | 0.015 |
| rs10978435 | C | -0.015 | 0.002 |  |  | 0.024 | 0.014 |  |  | 0.003 | 0.017 | 0.006 | 0.01 | -0.038 | 0.013 | -0.001 | 0.012 | -0.007 | 0.012 | 0.008 | 0.015 | 0.013 | 0.016 |
| rs10992812 | A | 0.012 | 0.002 | 0.006 | 0.01 | 0.015 | 0.014 | 0.009 | 0.011 | 0.001 | 0.016 | -0.003 | 0.007 | 0.022 | 0.013 | 0.001 | 0.008 | 0.004 | 0.012 | 0.001 | 0.01 | -0.024 | 0.016 |
| rs11240331 | T | 0.014 | 0.002 | -0.012 | 0.011 | 0.011 | 0.016 | 0.003 | 0.012 | 0.016 | 0.019 | 0 | 0.008 | 0.018 | 0.015 | 0.004 | 0.009 | 0.025 | 0.014 | 0.001 | 0.01 | 0.023 | 0.018 |
| rs112523595 | C | 0.024 | 0.002 | 0.006 | 0.01 | 0.014 | 0.014 | 0 | 0.011 | 0.023 | 0.017 | -0.006 | 0.011 | -0.018 | 0.014 | -0.021 | 0.012 | -0.02 | 0.012 | -0.002 | 0.01 | 0.002 | 0.017 |
| rs112633616 | C | 0.039 | 0.006 | -0.012 | 0.036 | 0.003 | 0.063 | -0.011 | 0.039 | 0.022 | 0.075 | -0.012 | 0.02 | 0.009 | 0.06 | -0.021 | 0.024 | 0.058 | 0.054 | -0.002 | 0.01 | 0.123 | 0.074 |
| rs112880127 | G | 0.015 | 0.003 | 0.003 | 0.014 | -0.006 | 0.018 | 0.018 | 0.015 | 0.019 | 0.022 | 0.02 | 0.013 | 0.001 | 0.017 | -0.015 | 0.016 | -0.014 | 0.016 | 0.001 | 0.01 | 0.029 | 0.021 |
| rs113247159 | C | 0.016 | 0.002 | -0.031 | 0.014 | -0.009 | 0.015 | -0.047 | 0.015 | -0.015 | 0.017 | -0.011 | 0.008 | -0.021 | 0.014 | 0.006 | 0.009 | -0.018 | 0.013 | 0.005 | 0.011 | -0.006 | 0.017 |
| rs113338260 | C | -0.015 | 0.003 | 0.025 | 0.012 | -0.005 | 0.016 | 0.017 | 0.014 | 0.008 | 0.019 | 0.004 | 0.009 | 0.002 | 0.015 | 0.007 | 0.01 | -0.015 | 0.014 | -0.007 | 0.015 | 0.023 | 0.019 |
| rs1156981 | G | -0.021 | 0.004 | -0.005 | 0.015 | -0.013 | 0.023 | -0.001 | 0.016 | -0.016 | 0.028 | -0.007 | 0.011 | 0.014 | 0.022 | 0.007 | 0.013 | 0.012 | 0.02 | 0.007 | 0.011 | 0.061 | 0.027 |
| rs11678980 | A | -0.012 | 0.002 | 0.009 | 0.011 | -0.013 | 0.013 | 0.017 | 0.011 | -0.001 | 0.016 | 0.005 | 0.007 | 0.001 | 0.013 | -0.009 | 0.008 | 0.005 | 0.012 | 0.01 | 0.018 | 0.001 | 0.016 |
| rs11688027 | G | 0.022 | 0.004 | 0.009 | 0.02 | -0.054 | 0.043 | 0.01 | 0.021 | -0.099 | 0.051 | 0.02 | 0.013 | -0.045 | 0.04 | 0.002 | 0.016 | -0.01 | 0.037 | -0.006 | 0.01 | -0.085 | 0.049 |
| rs11729080 | A | 0.022 | 0.003 | -0.01 | 0.014 | -0.008 | 0.019 | -0.01 | 0.016 | -0.028 | 0.023 | -0.019 | 0.009 | -0.076 | 0.018 | -0.028 | 0.011 | -0.025 | 0.016 | 0.001 | 0.011 | -0.011 | 0.022 |
| rs117831144 | T | -0.036 | 0.006 | 0.044 | 0.038 | 0.051 | 0.033 | 0.074 | 0.042 | 0.032 | 0.039 | -0.018 | 0.022 | -0.058 | 0.031 | -0.019 | 0.025 | -0.034 | 0.028 | 0.018 | 0.011 | -0.067 | 0.038 |
| rs11866420 | G | -0.017 | 0.002 |  |  |  |  |  |  |  |  |  |  |  |  |  |  |  |  |  |  |  |  |
| rs12049116 | T | 0.015 | 0.003 | -0.013 | 0.011 | -0.018 | 0.015 | -0.006 | 0.012 | -0.013 | 0.018 | 0.005 | 0.008 | 0.006 | 0.014 | 0.005 | 0.009 | -0.007 | 0.013 | 0.021 | 0.01 | 0.026 | 0.017 |
| rs12147463 | A | -0.020 | 0.003 | -0.014 | 0.011 | 0.017 | 0.018 | -0.009 | 0.012 | 0.012 | 0.022 | -0.013 | 0.008 | 0.015 | 0.017 | -0.01 | 0.01 | 0.008 | 0.016 | -0.029 | 0.012 | 0 | 0.021 |
| rs12203592 | T | 0.014 | 0.003 | 0.007 | 0.022 | 0.052 | 0.039 | -0.011 | 0.024 | 0.047 | 0.047 | 0.009 | 0.01 | 0.03 | 0.037 | 0.002 | 0.012 | 0.031 | 0.034 | -0.002 | 0.011 | 0.006 | 0.045 |
| rs12204714 | T | 0.028 | 0.002 | -0.001 | 0.01 | 0.003 | 0.013 | -0.004 | 0.011 | -0.006 | 0.016 | -0.007 | 0.007 | 0.007 | 0.013 | -0.005 | 0.008 | 0.022 | 0.012 | 0.01 | 0.012 | -0.013 | 0.016 |
| rs12244388 | A | -0.014 | 0.002 | -0.036 | 0.009 | -0.03 | 0.013 | -0.03 | 0.01 | -0.027 | 0.016 | 0.013 | 0.007 | 0.031 | 0.013 | -0.02 | 0.008 | -0.005 | 0.012 | -0.025 | 0.011 | 0.021 | 0.016 |
| rs1226414 | T | 0.014 | 0.002 |  |  |  |  |  |  |  |  |  |  |  |  |  |  |  |  |  |  |  |  |
| rs12292980 | A | -0.013 | 0.002 | 0.024 | 0.01 | 0.002 | 0.014 | 0.025 | 0.011 | 0.005 | 0.016 | 0.006 | 0.007 | 0.003 | 0.013 | 0.004 | 0.008 | 0.016 | 0.012 | 0.011 | 0.01 | 0.015 | 0.016 |
| rs12376530 | C | -0.015 | 0.002 | 0 | 0.01 | 0.018 | 0.015 | -0.003 | 0.011 | 0.029 | 0.018 | 0.007 | 0.008 | -0.018 | 0.014 | -0.004 | 0.009 | 0.003 | 0.013 | 0.016 | 0.01 | -0.033 | 0.017 |
| rs12448731 | T | -0.018 | 0.003 | -0.007 | 0.013 | 0.011 | 0.018 | 0.001 | 0.015 | 0.001 | 0.021 | 0.009 | 0.009 | 0.01 | 0.017 | 0.005 | 0.011 | 0.03 | 0.015 | -0.02 | 0.012 | 0.014 | 0.021 |
| rs12463727 | A | 0.014 | 0.002 | -0.024 | 0.009 | -0.01 | 0.013 | -0.024 | 0.01 | -0.018 | 0.016 | 0.009 | 0.007 | 0.002 | 0.012 | -0.028 | 0.008 | -0.008 | 0.011 | -0.006 | 0.012 | -0.027 | 0.015 |
| rs12511982 | A | 0.012 | 0.002 | -0.004 | 0.009 | 0.004 | 0.013 | 0.007 | 0.01 | -0.008 | 0.016 | -0.01 | 0.007 | -0.011 | 0.012 | -0.015 | 0.008 | -0.021 | 0.011 | -0.003 | 0.01 | 0.007 | 0.015 |
| rs12523097 | C | -0.014 | 0.002 | -0.006 | 0.01 | -0.031 | 0.016 | -0.011 | 0.011 | -0.021 | 0.019 | 0.009 | 0.007 | -0.01 | 0.015 | 0.01 | 0.009 | 0.002 | 0.014 | -0.011 | 0.01 | 0.014 | 0.019 |
| rs12523398 | A | 0.022 | 0.003 |  |  |  |  |  |  |  |  |  |  |  |  |  |  |  |  |  |  |  |  |
| rs12554512 | C | 0.013 | 0.002 | -0.004 | 0.009 | -0.006 | 0.013 | -0.002 | 0.011 | -0.001 | 0.016 | 0 | 0.007 | 0.002 | 0.013 | 0.017 | 0.008 | -0.007 | 0.011 | 0.032 | 0.014 | 0.003 | 0.015 |
| rs12653396 | A | -0.020 | 0.002 |  |  |  |  |  |  |  |  |  |  |  |  |  |  |  |  |  |  |  |  |
| rs12714592 | C | -0.022 | 0.002 | 0 | 0.01 |  |  | -0.005 | 0.011 |  |  | 0.013 | 0.007 |  |  | 0.013 | 0.009 |  |  | 0.021 | 0.01 |  |  |
| rs12714702 | G | -0.024 | 0.003 | 0.008 | 0.013 | 0.005 | 0.017 | 0.008 | 0.015 | 0.023 | 0.021 | 0 | 0.009 | 0.011 | 0.016 | 0.009 | 0.011 | 0.008 | 0.015 | -0.014 | 0.011 | 0.048 | 0.02 |
| rs12757779 | A | 0.013 | 0.003 | 0.004 | 0.012 | 0.018 | 0.016 | 0.008 | 0.014 | 0.025 | 0.019 | -0.006 | 0.008 | 0.009 | 0.015 | -0.004 | 0.01 | 0.028 | 0.013 | -0.018 | 0.017 | -0.015 | 0.018 |
| rs12878359 | C | 0.013 | 0.002 | -0.018 | 0.01 |  |  | -0.025 | 0.011 |  |  | 0.004 | 0.009 |  |  | 0.021 | 0.012 |  |  | 0.013 | 0.011 |  |  |
| rs12907546 | A | -0.023 | 0.003 | -0.012 | 0.012 | 0.009 | 0.016 | -0.008 | 0.013 | -0.007 | 0.019 | 0.011 | 0.008 | 0.021 | 0.015 | -0.005 | 0.01 | 0.017 | 0.013 | 0.019 | 0.011 | 0.018 | 0.018 |
| rs13175535 | A | 0.013 | 0.002 | -0.012 | 0.011 | -0.005 | 0.014 | -0.009 | 0.012 | -0.015 | 0.016 | -0.004 | 0.008 | -0.01 | 0.013 | 0.004 | 0.009 | 0.01 | 0.012 | -0.003 | 0.022 | 0.034 | 0.016 |
| rs13178956 | T | -0.016 | 0.002 |  |  |  |  |  |  |  |  |  |  |  |  |  |  |  |  |  |  |  |  |
| rs1320138 | C | 0.015 | 0.002 | -0.026 | 0.01 | -0.008 | 0.013 | -0.022 | 0.01 | -0.024 | 0.016 | -0.011 | 0.007 | -0.012 | 0.012 | 0.002 | 0.008 | -0.025 | 0.011 | -0.002 | 0.014 | -0.004 | 0.015 |
| rs1320330 | G | -0.018 | 0.003 | 0.037 | 0.012 | 0.021 | 0.018 | 0.015 | 0.013 | 0.018 | 0.021 | 0.038 | 0.009 | 0.031 | 0.017 | 0.031 | 0.01 | 0.019 | 0.015 | 0.01 | 0.023 | -0.011 | 0.02 |
| rs13280592 | G | -0.013 | 0.002 |  |  |  |  |  |  |  |  |  |  |  |  |  |  |  |  |  |  |  |  |
| rs13307225 | A | 0.022 | 0.003 | 0.012 | 0.016 | 0 | 0.029 | 0.017 | 0.017 | -0.018 | 0.036 | -0.006 | 0.011 | -0.008 | 0.028 | -0.012 | 0.014 | -0.005 | 0.025 | -0.004 | 0.012 | 0.041 | 0.034 |
| rs13394795 | C | 0.013 | 0.002 | 0.006 | 0.012 | 0.022 | 0.016 | 0.01 | 0.012 | 0.018 | 0.019 | -0.02 | 0.008 | -0.034 | 0.015 | -0.016 | 0.009 | 0.01 | 0.014 | 0.028 | 0.012 | -0.015 | 0.018 |
| rs1368546 | C | 0.017 | 0.002 | -0.011 | 0.01 | -0.002 | 0.013 | -0.01 | 0.011 | -0.009 | 0.016 | -0.014 | 0.007 | -0.017 | 0.013 | -0.016 | 0.008 | -0.017 | 0.011 | 0.008 | 0.011 | -0.01 | 0.015 |
| rs1392816 | T | 0.017 | 0.002 | -0.01 | 0.01 | -0.012 | 0.014 | -0.016 | 0.011 | -0.027 | 0.016 | -0.013 | 0.007 | -0.049 | 0.013 | -0.002 | 0.008 | -0.011 | 0.012 | 0.031 | 0.025 | -0.005 | 0.016 |
| rs141547796 | A | 0.032 | 0.004 | -0.01 | 0.017 | -0.003 | 0.018 | -0.009 | 0.019 | -0.003 | 0.021 | -0.016 | 0.013 | -0.009 | 0.017 | 0.003 | 0.015 | -0.021 | 0.015 | -0.021 | 0.032 | -0.032 | 0.021 |
| rs147633738 | T | -0.024 | 0.004 | 0.024 | 0.023 | 0.025 | 0.034 | 0.006 | 0.025 | 0.003 | 0.041 | 0.017 | 0.014 | 0.06 | 0.032 | 0.026 | 0.016 | 0.057 | 0.029 | 0.001 | 0.021 | 0.043 | 0.039 |
| rs147725178 | T | -0.031 | 0.005 | 0.042 | 0.03 | 0.001 | 0.039 | 0.04 | 0.033 | 0.003 | 0.046 | 0.018 | 0.019 | -0.067 | 0.037 | 0.047 | 0.021 | -0.01 | 0.033 | 0.006 | 0.01 | -0.023 | 0.045 |
| rs1547351 | A | 0.012 | 0.002 |  |  |  |  |  |  |  |  |  |  |  |  |  |  |  |  |  |  |  |  |
| rs1585634 | C | -0.015 | 0.003 |  |  |  |  |  |  |  |  |  |  |  |  |  |  |  |  |  |  |  |  |
| rs1609598 | T | -0.012 | 0.002 | 0.009 | 0.01 | 0.013 | 0.013 | 0.01 | 0.011 | 0.005 | 0.016 | -0.011 | 0.007 | 0.016 | 0.013 | 0.006 | 0.008 | 0.021 | 0.011 | 0.015 | 0.01 | 0.017 | 0.015 |
| rs1866710 | G | 0.015 | 0.002 | -0.013 | 0.01 | 0.002 | 0.016 | -0.018 | 0.011 | -0.002 | 0.019 | -0.015 | 0.008 | -0.01 | 0.015 | -0.006 | 0.009 | -0.015 | 0.014 | 0.019 | 0.014 | 0.026 | 0.019 |
| rs186723454 | G | -0.018 | 0.003 | 0.011 | 0.012 | 0.021 | 0.021 | 0.003 | 0.014 | -0.013 | 0.026 | -0.016 | 0.017 | 0.013 | 0.02 | 0.005 | 0.016 | 0.004 | 0.018 | -0.011 | 0.012 | 0.012 | 0.025 |
| rs191242857 | A | 0.013 | 0.002 | -0.01 | 0.012 | 0.021 | 0.017 | -0.004 | 0.013 | 0.001 | 0.02 | 0.019 | 0.011 | -0.007 | 0.016 | -0.02 | 0.014 | 0.009 | 0.015 | 0.003 | 0.01 | -0.041 | 0.02 |
| rs1925686 | A | 0.012 | 0.002 | -0.008 | 0.009 | 0 | 0.013 | -0.007 | 0.01 | -0.003 | 0.016 | -0.042 | 0.007 | -0.033 | 0.013 | -0.031 | 0.008 | -0.001 | 0.012 | 0.007 | 0.01 | 0.028 | 0.016 |
| rs1931263 | T | -0.012 | 0.002 | 0.014 | 0.01 | -0.016 | 0.013 | 0.012 | 0.011 | -0.027 | 0.016 | -0.002 | 0.007 | 0.001 | 0.013 | -0.001 | 0.008 | 0.012 | 0.011 | 0.013 | 0.018 | -0.014 | 0.015 |
| rs1962545 | C | -0.012 | 0.002 | 0.008 | 0.01 | 0.009 | 0.013 | 0.004 | 0.011 | 0.015 | 0.016 | 0.001 | 0.007 | -0.007 | 0.012 | 0.002 | 0.008 | 0.011 | 0.011 | 0.011 | 0.012 | -0.006 | 0.015 |
| rs1976423 | C | -0.011 | 0.002 |  |  |  |  |  |  |  |  | 0.01 | 0.007 |  |  | 0 | 0.008 |  |  | 0.002 | 0.011 |  |  |
| rs1991651 | G | 0.018 | 0.002 |  |  |  |  |  |  |  |  |  |  |  |  |  |  |  |  |  |  |  |  |
| rs1995181 | A | -0.012 | 0.002 |  |  |  |  |  |  |  |  |  |  |  |  |  |  |  |  |  |  |  |  |
| rs2084572 | G | 0.014 | 0.002 | -0.002 | 0.01 | -0.005 | 0.013 | -0.01 | 0.011 | -0.025 | 0.016 | -0.007 | 0.007 | 0.005 | 0.013 | -0.008 | 0.008 | -0.007 | 0.011 | -0.007 | 0.013 | 0.006 | 0.015 |
| rs2091377 | T | 0.012 | 0.002 | -0.018 | 0.009 | -0.006 | 0.013 | -0.011 | 0.01 | -0.012 | 0.016 | -0.01 | 0.007 | -0.008 | 0.012 | -0.012 | 0.008 | -0.008 | 0.011 | -0.02 | 0.011 | -0.023 | 0.015 |
| rs2093623 | A | 0.014 | 0.002 | -0.014 | 0.009 | -0.001 | 0.013 | -0.007 | 0.01 | 0.016 | 0.016 | -0.012 | 0.007 | -0.01 | 0.012 | 0.006 | 0.008 | -0.003 | 0.011 | 0.009 | 0.012 | 0.023 | 0.015 |
| rs2130894 | T | 0.013 | 0.002 | 0.019 | 0.013 | -0.013 | 0.014 | 0.013 | 0.014 | -0.017 | 0.017 | 0.006 | 0.008 | 0.013 | 0.013 | -0.001 | 0.009 | -0.013 | 0.012 | -0.015 | 0.01 | -0.001 | 0.016 |
| rs215639 | T | 0.013 | 0.002 | -0.014 | 0.01 | -0.027 | 0.015 | -0.016 | 0.011 | -0.014 | 0.018 | -0.008 | 0.007 | -0.018 | 0.014 | -0.009 | 0.008 | -0.016 | 0.013 | 0.012 | 0.01 | 0.01 | 0.017 |
| rs2174752 | T | -0.013 | 0.002 | 0.011 | 0.009 | 0.014 | 0.013 | 0.014 | 0.01 | 0.002 | 0.016 | -0.007 | 0.007 | -0.025 | 0.012 | -0.011 | 0.008 | 0.005 | 0.011 | 0.006 | 0.011 | -0.004 | 0.015 |
| rs2176337 | T | -0.015 | 0.002 |  |  |  |  |  |  |  |  |  |  |  |  |  |  |  |  |  |  |  |  |
| rs2188151 | T | -0.021 | 0.002 | 0.018 | 0.01 | -0.022 | 0.014 | 0.021 | 0.011 | -0.019 | 0.017 | -0.005 | 0.007 | -0.005 | 0.013 | 0.022 | 0.008 | -0.005 | 0.012 | -0.007 | 0.012 | -0.017 | 0.016 |
| rs222440 | C | 0.017 | 0.003 | -0.006 | 0.013 | -0.014 | 0.018 | -0.014 | 0.014 | -0.045 | 0.021 | -0.004 | 0.009 | 0.008 | 0.017 | 0.018 | 0.01 | -0.026 | 0.015 | -0.009 | 0.011 | 0.012 | 0.021 |
| rs2274568 | A | -0.013 | 0.002 | 0.014 | 0.01 | 0.014 | 0.013 | 0.016 | 0.011 | 0.02 | 0.016 | 0.002 | 0.007 | 0.013 | 0.013 | 0.008 | 0.008 | 0.016 | 0.012 | -0.023 | 0.032 | 0.017 | 0.016 |
| rs2279574 | A | 0.012 | 0.002 | -0.016 | 0.009 | -0.01 | 0.013 | -0.014 | 0.01 | -0.02 | 0.016 | -0.011 | 0.007 | 0.002 | 0.013 | -0.012 | 0.008 | -0.009 | 0.012 | 0.003 | 0.015 | 0.003 | 0.016 |
| rs2406374 | T | 0.014 | 0.002 | -0.003 | 0.011 | -0.006 | 0.015 | -0.012 | 0.012 | -0.002 | 0.018 | -0.002 | 0.007 | 0.004 | 0.014 | -0.004 | 0.009 | -0.005 | 0.013 | -0.016 | 0.013 | -0.001 | 0.017 |
| rs2612030 | C | 0.024 | 0.003 | 0.024 | 0.014 | 0.001 | 0.021 | 0.028 | 0.015 | 0.012 | 0.026 | 0.011 | 0.009 | -0.043 | 0.02 | -0.009 | 0.011 | -0.019 | 0.018 | -0.005 | 0.013 | -0.018 | 0.025 |
| rs2650705 | G | -0.017 | 0.003 | 0.012 | 0.011 | 0.024 | 0.02 | 0.009 | 0.013 | 0.032 | 0.024 | -0.015 | 0.009 | -0.008 | 0.019 | 0 | 0.011 | 0.009 | 0.018 | 0.013 | 0.017 | -0.058 | 0.024 |
| rs28406364 | T | -0.017 | 0.002 | 0.037 | 0.01 | 0.028 | 0.013 | 0.032 | 0.011 | 0.034 | 0.016 | 0.016 | 0.007 | 0.031 | 0.013 | 0.006 | 0.008 | 0.016 | 0.011 | 0.004 | 0.011 | 0.034 | 0.015 |
| rs28929474 | T | 0.042 | 0.007 | -0.147 | 0.045 | -0.044 | 0.047 | -0.148 | 0.052 | -0.073 | 0.057 | 0.022 | 0.028 | 0.021 | 0.045 | 0.026 | 0.034 | -0.022 | 0.041 | 0.017 | 0.02 | -0.027 | 0.055 |
| rs2974311 | A | 0.013 | 0.002 | -0.009 | 0.009 | -0.019 | 0.013 | -0.008 | 0.01 | -0.019 | 0.016 | 0.012 | 0.007 | 0.004 | 0.012 | 0 | 0.008 | 0.001 | 0.011 | 0.008 | 0.014 | 0.043 | 0.015 |
| rs3007104 | A | -0.015 | 0.002 | 0.006 | 0.009 | -0.001 | 0.014 | 0.002 | 0.01 | 0.005 | 0.016 | 0.01 | 0.007 | -0.004 | 0.013 | 0.023 | 0.008 | 0.012 | 0.012 | -0.042 | 0.035 | 0.024 | 0.016 |
| rs341521 | A | -0.016 | 0.002 | 0.013 | 0.011 | -0.019 | 0.015 | 0.018 | 0.012 | -0.028 | 0.018 | 0.002 | 0.007 | -0.003 | 0.014 | 0.001 | 0.009 | -0.002 | 0.013 | -0.015 | 0.011 | -0.006 | 0.017 |
| rs34155040 | T | 0.014 | 0.002 | -0.027 | 0.009 | 0.002 | 0.013 | -0.017 | 0.01 | -0.015 | 0.016 | 0.003 | 0.009 | -0.001 | 0.012 | -0.023 | 0.012 | -0.03 | 0.011 | 0.011 | 0.012 | 0.006 | 0.015 |
| rs3447 | G | 0.018 | 0.003 |  |  |  |  |  |  |  |  |  |  |  |  |  |  |  |  |  |  |  |  |
| rs34517439 | A | -0.02 | 0.003 | 0.038 | 0.019 | -0.003 | 0.02 | 0.045 | 0.021 | 0.033 | 0.024 | 0.023 | 0.011 | -0.007 | 0.019 | 0.018 | 0.012 | 0.031 | 0.017 | 0.004 | 0.016 | 0.045 | 0.023 |
| rs34606772 | T | -0.012 | 0.002 | 0.012 | 0.01 | 0.022 | 0.013 | 0.012 | 0.011 | 0.016 | 0.016 | 0.019 | 0.007 | 0.001 | 0.012 | 0.009 | 0.008 | -0.016 | 0.011 | 0.005 | 0.01 | -0.024 | 0.015 |
| rs34804222 | G | 0.015 | 0.002 | -0.011 | 0.01 | -0.041 | 0.013 | -0.011 | 0.011 | -0.035 | 0.016 | -0.012 | 0.007 | -0.009 | 0.013 | -0.03 | 0.008 | -0.023 | 0.011 | 0.012 | 0.01 | -0.021 | 0.015 |
| rs34811474 | A | 0.016 | 0.002 | -0.011 | 0.015 | -0.029 | 0.016 | 0.007 | 0.016 | -0.035 | 0.019 | -0.025 | 0.008 | -0.044 | 0.015 | -0.021 | 0.01 | -0.043 | 0.013 | 0.008 | 0.01 | -0.027 | 0.018 |
| rs35077383 | C | -0.011 | 0.002 | 0.007 | 0.009 | -0.002 | 0.013 | 0.014 | 0.01 | 0.005 | 0.016 | 0.014 | 0.007 | 0.012 | 0.012 | 0.001 | 0.008 | 0.003 | 0.011 | 0.015 | 0.011 | 0.015 | 0.015 |
| rs35851551 | G | -0.025 | 0.003 | -0.034 | 0.02 | -0.036 | 0.028 | -0.033 | 0.022 | -0.047 | 0.033 | -0.017 | 0.012 | 0.034 | 0.027 | 0.01 | 0.014 | -0.063 | 0.024 | 0.011 | 0.012 | -0.036 | 0.032 |
| rs359271 | C | -0.018 | 0.002 | 0.006 | 0.01 | -0.012 | 0.013 | 0.003 | 0.01 | -0.014 | 0.016 | 0.006 | 0.009 | 0.002 | 0.013 | 0.011 | 0.012 | 0 | 0.011 | -0.001 | 0.013 | 0.019 | 0.015 |
| rs3866460 | C | 0.019 | 0.003 | 0.023 | 0.013 |  |  | 0.036 | 0.015 |  |  | 0.022 | 0.018 |  |  | 0.01 | 0.02 |  |  | -0.011 | 0.01 |  |  |
| rs3896224 | G | 0.02 | 0.002 | -0.012 | 0.009 | 0.006 | 0.013 | -0.018 | 0.01 | 0.002 | 0.016 | -0.001 | 0.007 | 0.008 | 0.012 | -0.013 | 0.009 | 0.013 | 0.011 | -0.014 | 0.01 | -0.007 | 0.015 |
| rs410520 | T | 0.011 | 0.002 | 0.005 | 0.01 | 0.006 | 0.013 | -0.006 | 0.011 | -0.018 | 0.016 | -0.017 | 0.007 | -0.018 | 0.013 | -0.011 | 0.008 | -0.014 | 0.011 | -0.004 | 0.011 | -0.004 | 0.015 |
| rs4246175 | A | 0.014 | 0.002 |  |  |  |  |  |  |  |  |  |  |  |  |  |  |  |  |  |  |  |  |
| rs435538 | G | -0.017 | 0.002 |  |  |  |  |  |  |  |  |  |  |  |  |  |  |  |  |  |  |  |  |
| rs4439537 | C | 0.013 | 0.002 | 0.006 | 0.009 | -0.004 | 0.013 | -0.001 | 0.011 | -0.004 | 0.016 | -0.002 | 0.007 | -0.014 | 0.012 | -0.013 | 0.008 | -0.016 | 0.011 | 0.019 | 0.01 | -0.029 | 0.015 |
| rs4557006 | A | -0.012 | 0.002 | 0.007 | 0.01 | -0.007 | 0.013 | 0.009 | 0.011 | -0.017 | 0.016 | 0.008 | 0.007 | 0.016 | 0.013 | 0.018 | 0.008 | -0.009 | 0.011 | 0 | 0.011 | -0.009 | 0.015 |
| rs4569188 | A | -0.014 | 0.002 | 0.015 | 0.01 | 0.008 | 0.013 | 0.023 | 0.011 | 0.014 | 0.016 | 0.001 | 0.007 | -0.004 | 0.013 | -0.001 | 0.01 | 0.007 | 0.012 | -0.009 | 0.014 | -0.024 | 0.016 |
| rs4602427 | G | -0.017 | 0.003 |  |  |  |  |  |  |  |  |  |  |  |  |  |  |  |  |  |  |  |  |
| rs4702 | A | 0.017 | 0.002 | 0.038 | 0.01 | 0.053 | 0.013 | 0.043 | 0.012 | 0.037 | 0.016 | 0.005 | 0.007 | 0.024 | 0.012 | 0.012 | 0.008 | 0.008 | 0.011 | 0.004 | 0.01 | 0.039 | 0.015 |
| rs4709807 | C | -0.014 | 0.002 | 0.011 | 0.011 | -0.005 | 0.017 | 0.009 | 0.012 | -0.003 | 0.02 | 0.006 | 0.008 | 0.004 | 0.016 | 0.002 | 0.009 | -0.007 | 0.014 | -0.007 | 0.012 | -0.018 | 0.019 |
| rs4728298 | A | -0.022 | 0.003 |  |  |  |  |  |  |  |  |  |  |  |  |  |  |  |  |  |  |  |  |
| rs4800204 | T | -0.013 | 0.002 | 0.008 | 0.009 | 0.001 | 0.013 | 0.01 | 0.01 | 0.002 | 0.016 | 0.006 | 0.007 | 0.005 | 0.012 | -0.007 | 0.008 | 0.014 | 0.011 | 0.011 | 0.011 | 0.015 | 0.015 |
| rs4809230 | A | 0.023 | 0.004 | 0.002 | 0.016 | -0.01 | 0.052 | 0.002 | 0.019 | -0.001 | 0.063 | -0.026 | 0.013 | -0.013 | 0.05 | -0.031 | 0.015 | -0.059 | 0.045 | 0 | 0.011 | 0.025 | 0.06 |
| rs4868800 | T | 0.013 | 0.002 | -0.007 | 0.01 | 0.008 | 0.013 | -0.015 | 0.011 | 0.013 | 0.016 | -0.004 | 0.007 | 0.027 | 0.013 | 0.003 | 0.008 | 0.013 | 0.012 | 0.004 | 0.013 | 0.012 | 0.016 |
| rs4952343 | G | 0.013 | 0.002 | 0.004 | 0.01 | -0.018 | 0.013 | 0.008 | 0.011 | -0.027 | 0.016 | -0.005 | 0.007 | 0.019 | 0.013 | -0.007 | 0.008 | 0.004 | 0.011 | -0.013 | 0.012 | 0.002 | 0.015 |
| rs4961705 | C | 0.013 | 0.002 |  |  |  |  |  |  |  |  |  |  |  |  |  |  |  |  |  |  |  |  |
| rs56306056 | A | 0.016 | 0.003 | -0.008 | 0.013 | -0.025 | 0.016 | 0.004 | 0.014 | -0.035 | 0.02 | 0.012 | 0.008 | 0.01 | 0.016 | -0.004 | 0.01 | 0.019 | 0.014 | -0.004 | 0.012 | 0.005 | 0.019 |
| rs56392241 | C | -0.014 | 0.002 | 0.011 | 0.01 | 0.004 | 0.013 | 0.006 | 0.011 | -0.007 | 0.016 | 0.004 | 0.007 | 0.037 | 0.013 | 0.006 | 0.008 | 0.015 | 0.012 | -0.006 | 0.01 | -0.009 | 0.016 |
| rs58938116 | T | -0.018 | 0.003 | -0.019 | 0.014 | 0.024 | 0.017 | -0.006 | 0.016 | 0.039 | 0.02 | 0.017 | 0.009 | 0.022 | 0.016 | 0.022 | 0.011 | -0.023 | 0.014 | -0.013 | 0.011 | 0.011 | 0.019 |
| rs590414 | T | 0.014 | 0.002 |  |  |  |  |  |  |  |  |  |  |  |  |  |  |  |  |  |  |  |  |
| rs592278 | A | -0.013 | 0.002 | 0.019 | 0.01 | -0.018 | 0.013 | 0.024 | 0.011 | -0.006 | 0.016 | 0.002 | 0.01 | 0.009 | 0.013 | 0.011 | 0.012 | 0.007 | 0.012 | 0.03 | 0.022 | 0.033 | 0.016 |
| rs6058613 | G | -0.017 | 0.003 |  |  |  |  |  |  |  |  |  |  |  |  |  |  |  |  |  |  |  |  |
| rs60775983 | G | 0.018 | 0.003 | 0.012 | 0.013 | -0.004 | 0.018 | 0.019 | 0.014 | 0.015 | 0.022 | 0.014 | 0.01 | -0.041 | 0.017 | 0 | 0.011 | -0.011 | 0.016 | -0.001 | 0.01 | -0.006 | 0.021 |
| rs61856978 | C | -0.015 | 0.002 | -0.002 | 0.01 | 0.037 | 0.014 | 0 | 0.011 | 0.039 | 0.017 | 0.001 | 0.01 | 0.006 | 0.013 | 0.017 | 0.012 | 0.016 | 0.012 | 0.027 | 0.013 | 0.014 | 0.016 |
| rs62134195 | T | 0.032 | 0.005 | -0.012 | 0.025 | 0.015 | 0.031 | -0.003 | 0.027 | 0.035 | 0.037 | 0.036 | 0.018 | 0.042 | 0.029 | -0.01 | 0.021 | 0.008 | 0.027 | -0.006 | 0.011 | 0.007 | 0.036 |
| rs62177795 | A | 0.019 | 0.003 | 0.012 | 0.012 | -0.018 | 0.018 | 0.004 | 0.013 | -0.002 | 0.022 | 0.001 | 0.009 | -0.018 | 0.017 | 0.027 | 0.012 | -0.023 | 0.016 | 0.008 | 0.012 | 0.003 | 0.021 |
| rs62439690 | A | -0.016 | 0.002 | 0.028 | 0.011 | 0.012 | 0.016 | 0.022 | 0.013 | 0.012 | 0.019 | 0.001 | 0.008 | -0.016 | 0.015 | 0.014 | 0.009 | 0.003 | 0.014 | 0.015 | 0.014 | 0.012 | 0.019 |
| rs6504551 | G | -0.015 | 0.002 | 0.022 | 0.01 | 0 | 0.014 | 0.039 | 0.011 | -0.01 | 0.017 | 0.002 | 0.01 | -0.009 | 0.013 | -0.002 | 0.013 | 0.015 | 0.012 | -0.001 | 0.012 | 0.016 | 0.016 |
| rs6517512 | G | -0.028 | 0.005 | 0.008 | 0.03 | 0 | 0.038 | -0.006 | 0.033 | 0.011 | 0.045 | -0.005 | 0.018 | 0.001 | 0.036 | 0 | 0.02 | 0.022 | 0.033 | -0.011 | 0.01 | 0.022 | 0.044 |
| rs6564268 | G | 0.027 | 0.005 |  |  |  |  |  |  |  |  |  |  |  |  |  |  |  |  |  |  |  |  |
| rs6586405 | A | -0.012 | 0.002 | 0.019 | 0.011 | 0.009 | 0.014 | 0.012 | 0.012 | 0.011 | 0.017 | 0.007 | 0.007 | 0.004 | 0.013 | 0.008 | 0.008 | 0.006 | 0.012 | 0.008 | 0.01 | 0.025 | 0.016 |
| rs6719762 | C | -0.022 | 0.002 | 0.011 | 0.01 | 0.006 | 0.013 | 0.015 | 0.01 | 0.01 | 0.016 | 0.002 | 0.007 | -0.014 | 0.013 | -0.003 | 0.008 | 0.003 | 0.012 | 0.017 | 0.013 | 0.02 | 0.016 |
| rs6744794 | G | -0.019 | 0.002 |  |  |  |  |  |  |  |  |  |  |  |  |  |  |  |  |  |  |  |  |
| rs6747099 | C | 0.012 | 0.002 |  |  |  |  |  |  |  |  |  |  |  |  |  |  |  |  |  |  |  |  |
| rs6748341 | G | 0.015 | 0.002 |  |  |  |  |  |  |  |  |  |  |  |  |  |  |  |  |  |  |  |  |
| rs67723420 | A | 0.015 | 0.002 |  |  |  |  |  |  |  |  |  |  |  |  |  |  |  |  |  |  |  |  |
| rs6776937 | A | -0.014 | 0.003 | 0.006 | 0.013 | -0.028 | 0.017 | -0.003 | 0.014 | -0.006 | 0.02 | -0.014 | 0.008 | 0.017 | 0.016 | 0 | 0.01 | 0.009 | 0.014 | 0.003 | 0.01 | -0.017 | 0.019 |
| rs6966769 | G | 0.018 | 0.003 | 0.014 | 0.018 | 0.007 | 0.018 | 0.029 | 0.02 | 0.022 | 0.022 | 0.002 | 0.011 | 0 | 0.017 | 0.004 | 0.013 | -0.002 | 0.016 | 0.003 | 0.012 | -0.002 | 0.021 |
| rs6978112 | T | -0.016 | 0.002 | 0.015 | 0.01 | 0.01 | 0.013 | 0.014 | 0.011 | 0.01 | 0.016 | 0.006 | 0.007 | 0.009 | 0.013 | -0.006 | 0.008 | 0.018 | 0.011 | -0.006 | 0.011 | -0.004 | 0.015 |
| rs7008955 | G | 0.012 | 0.002 | 0.021 | 0.009 | 0.019 | 0.013 | 0.003 | 0.01 | 0.03 | 0.016 | -0.006 | 0.007 | 0.005 | 0.012 | -0.004 | 0.008 | -0.01 | 0.011 | 0.014 | 0.01 | 0.026 | 0.015 |
| rs702 | T | 0.018 | 0.003 |  |  |  |  |  |  |  |  |  |  |  |  |  |  |  |  |  |  |  |  |
| rs705240 | T | -0.015 | 0.003 | 0.008 | 0.012 | -0.012 | 0.015 | -0.008 | 0.014 | 0.004 | 0.018 | 0.005 | 0.009 | 0.013 | 0.014 | 0.015 | 0.01 | 0.014 | 0.013 | 0.009 | 0.01 | 0.006 | 0.017 |
| rs7079070 | A | -0.017 | 0.002 | -0.005 | 0.01 | -0.005 | 0.014 | -0.005 | 0.011 | -0.007 | 0.017 | -0.001 | 0.007 | 0.004 | 0.013 | 0.005 | 0.008 | -0.014 | 0.012 | -0.009 | 0.013 | 0.013 | 0.016 |
| rs7091634 | G | 0.013 | 0.002 | -0.002 | 0.01 | -0.023 | 0.014 | 0.009 | 0.011 | -0.026 | 0.017 | 0.009 | 0.011 | -0.019 | 0.014 | 0.009 | 0.012 | -0.017 | 0.012 | 0.004 | 0.01 | -0.022 | 0.017 |
| rs7110863 | G | -0.015 | 0.002 | -0.007 | 0.009 | 0.02 | 0.014 | -0.003 | 0.01 | 0.012 | 0.016 | 0.002 | 0.007 | 0.013 | 0.013 | 0.01 | 0.008 | 0.027 | 0.012 | -0.012 | 0.01 | 0.003 | 0.016 |
| rs714393 | T | 0.013 | 0.002 | -0.005 | 0.01 | -0.001 | 0.013 | -0.005 | 0.01 | -0.009 | 0.016 | -0.002 | 0.007 | -0.014 | 0.013 | -0.008 | 0.008 | -0.006 | 0.011 | -0.014 | 0.011 | 0.009 | 0.015 |
| rs7151954 | G | -0.021 | 0.003 | 0.019 | 0.011 | 0.015 | 0.015 | 0.017 | 0.013 | 0.027 | 0.018 | -0.003 | 0.008 | 0.011 | 0.014 | 0.02 | 0.01 | 0.026 | 0.013 | 0.011 | 0.014 | 0.022 | 0.018 |
| rs7152323 | G | -0.016 | 0.002 | 0.026 | 0.009 | -0.013 | 0.013 | 0.022 | 0.01 | -0.014 | 0.016 | 0.009 | 0.007 | -0.012 | 0.013 | 0.014 | 0.008 | -0.001 | 0.011 | -0.002 | 0.014 | -0.002 | 0.015 |
| rs7167444 | T | -0.014 | 0.002 | -0.012 | 0.011 | 0.009 | 0.014 | -0.009 | 0.013 | 0.002 | 0.017 | 0.007 | 0.008 | -0.018 | 0.013 | 0 | 0.009 | -0.012 | 0.012 | 0.004 | 0.01 | -0.022 | 0.016 |
| rs7188873 | G | -0.015 | 0.002 | 0.007 | 0.01 | -0.007 | 0.013 | 0.014 | 0.011 | -0.01 | 0.016 | 0.009 | 0.007 | 0.003 | 0.013 | 0.021 | 0.008 | 0.009 | 0.012 | 0.036 | 0.044 | -0.006 | 0.016 |
| rs7201310 | A | 0.012 | 0.002 | 0.008 | 0.01 | 0.005 | 0.014 | 0.011 | 0.011 | 0.003 | 0.017 | -0.003 | 0.007 | 0.004 | 0.013 | -0.005 | 0.009 | -0.011 | 0.012 | 0.004 | 0.01 | -0.002 | 0.016 |
| rs7236339 | A | -0.020 | 0.003 | 0 | 0.012 | 0.013 | 0.015 | 0.016 | 0.013 | 0.011 | 0.018 | -0.004 | 0.009 | 0.012 | 0.014 | -0.009 | 0.009 | 0.003 | 0.013 | 0.011 | 0.01 | -0.004 | 0.018 |
| rs72674824 | C | 0.014 | 0.002 | 0.028 | 0.011 | -0.009 | 0.014 | 0.032 | 0.012 | -0.012 | 0.016 | -0.003 | 0.008 | 0.003 | 0.013 | 0.006 | 0.009 | -0.002 | 0.012 | 0.012 | 0.011 | -0.005 | 0.016 |
| rs72887338 | C | -0.016 | 0.002 | 0.015 | 0.01 | 0.006 | 0.014 | 0.008 | 0.011 | 0.018 | 0.016 | 0.01 | 0.007 | 0.003 | 0.013 | 0.007 | 0.008 | 0.01 | 0.012 | -0.023 | 0.01 | 0.004 | 0.016 |
| rs7381195 | A | -0.014 | 0.002 |  |  |  |  |  |  |  |  |  |  |  |  |  |  |  |  |  |  |  |  |
| rs7452074 | C | -0.013 | 0.002 | 0.018 | 0.011 | 0.002 | 0.014 | 0.005 | 0.012 | -0.002 | 0.017 | -0.012 | 0.01 | -0.025 | 0.013 | 0.018 | 0.013 | 0.005 | 0.012 | 0.029 | 0.016 | -0.03 | 0.016 |
| rs74737734 | T | 0.035 | 0.006 |  |  |  |  |  |  |  |  |  |  |  |  |  |  |  |  |  |  |  |  |
| rs7476 | C | -0.014 | 0.002 | 0.021 | 0.011 | 0.011 | 0.016 | 0.028 | 0.012 | 0.001 | 0.02 | 0.001 | 0.007 | -0.004 | 0.016 | 0.015 | 0.009 | 0.004 | 0.014 | -0.027 | 0.01 | -0.041 | 0.019 |
| rs7503604 | A | 0.013 | 0.002 | -0.007 | 0.012 | -0.002 | 0.013 | -0.002 | 0.013 | -0.001 | 0.016 | -0.02 | 0.008 | -0.02 | 0.013 | -0.022 | 0.01 | -0.008 | 0.011 | -0.013 | 0.014 | -0.007 | 0.015 |
| rs7525548 | T | 0.015 | 0.002 |  |  |  |  |  |  |  |  |  |  |  |  |  |  |  |  |  |  |  |  |
| rs7566527 | T | 0.014 | 0.002 | -0.014 | 0.01 | -0.011 | 0.013 | -0.024 | 0.011 | -0.022 | 0.016 | 0.006 | 0.007 | -0.004 | 0.013 | 0 | 0.008 | -0.012 | 0.011 | 0.005 | 0.018 | -0.011 | 0.015 |
| rs7575189 | A | 0.016 | 0.002 | -0.009 | 0.01 | 0.008 | 0.013 | -0.003 | 0.01 | 0.009 | 0.016 | 0.007 | 0.007 | -0.017 | 0.013 | -0.011 | 0.008 | -0.002 | 0.011 | 0.004 | 0.011 | -0.01 | 0.015 |
| rs7618715 | A | 0.013 | 0.002 | -0.011 | 0.01 | 0.019 | 0.014 | -0.019 | 0.011 | 0.024 | 0.017 | 0.004 | 0.007 | -0.001 | 0.013 | 0.001 | 0.008 | 0 | 0.012 | -0.029 | 0.018 | 0.005 | 0.016 |
| rs763053 | C | 0.016 | 0.003 | 0.006 | 0.011 | -0.004 | 0.014 | 0.008 | 0.012 | -0.002 | 0.016 | 0.025 | 0.008 | 0.041 | 0.013 | 0.02 | 0.009 | 0.025 | 0.012 | 0.002 | 0.01 | 0.017 | 0.016 |
| rs76513770 | C | 0.024 | 0.003 | -0.021 | 0.014 | -0.002 | 0.017 | -0.024 | 0.016 | -0.01 | 0.021 |  |  | -0.033 | 0.017 | -0.021 | 0.012 | -0.019 | 0.015 | -0.015 | 0.01 | 0.023 | 0.02 |
| rs766406 | T | 0.014 | 0.002 | 0.004 | 0.01 | -0.005 | 0.014 | 0.011 | 0.011 | 0.002 | 0.017 | 0.001 | 0.007 | -0.009 | 0.013 | 0.001 | 0.008 | -0.022 | 0.012 | -0.025 | 0.011 | -0.027 | 0.016 |
| rs7671317 | T | 0.012 | 0.002 | 0.008 | 0.01 | 0.005 | 0.014 | 0.006 | 0.011 | 0.004 | 0.016 | -0.015 | 0.007 | -0.033 | 0.013 | -0.008 | 0.008 | -0.011 | 0.012 | 0.006 | 0.012 | 0.004 | 0.016 |
| rs767943 | A | -0.017 | 0.002 | 0.022 | 0.01 | 0.027 | 0.015 | 0.02 | 0.011 | 0.035 | 0.018 | 0.004 | 0.008 | 0.012 | 0.014 | 0.014 | 0.009 | 0.012 | 0.013 | 0.016 | 0.01 | 0.024 | 0.018 |
| rs7704530 | A | -0.013 | 0.002 | -0.014 | 0.01 | 0.022 | 0.014 | -0.013 | 0.011 | 0.017 | 0.017 |  |  | -0.002 | 0.013 | 0.005 | 0.009 | -0.002 | 0.012 | -0.006 | 0.01 | 0.008 | 0.016 |
| rs7783012 | A | -0.020 | 0.002 | 0.02 | 0.009 | 0.031 | 0.013 | 0.009 | 0.01 | 0.031 | 0.016 | 0.002 | 0.007 | 0.007 | 0.013 | -0.001 | 0.008 | 0.015 | 0.011 | 0.016 | 0.011 | 0.007 | 0.015 |
| rs7785195 | A | 0.014 | 0.002 | 0.014 | 0.01 | -0.008 | 0.014 | 0.017 | 0.011 | -0.01 | 0.017 | -0.011 | 0.007 | -0.009 | 0.013 | 0.003 | 0.008 | -0.003 | 0.012 | 0.013 | 0.012 | -0.014 | 0.016 |
| rs7804551 | G | 0.023 | 0.003 | -0.011 | 0.011 | -0.024 | 0.016 | -0.01 | 0.013 | -0.014 | 0.019 | 0.012 | 0.009 | 0.012 | 0.015 | -0.042 | 0.011 | -0.019 | 0.014 | 0.021 | 0.01 | 0.002 | 0.018 |
| rs7815125 | A | 0.018 | 0.003 |  |  |  |  |  |  |  |  |  |  |  |  |  |  |  |  |  |  |  |  |
| rs7824756 | C | -0.016 | 0.002 | -0.01 | 0.011 | 0.034 | 0.015 | -0.009 | 0.012 | 0.057 | 0.018 | -0.011 | 0.01 | -0.008 | 0.014 | 0.009 | 0.013 | -0.003 | 0.013 | -0.001 | 0.013 | 0.012 | 0.017 |
| rs783544 | C | 0.015 | 0.002 | -0.016 | 0.011 | 0.008 | 0.015 | -0.02 | 0.012 | -0.007 | 0.019 | -0.003 | 0.008 | -0.001 | 0.015 | -0.016 | 0.009 | -0.009 | 0.013 | 0.004 | 0.01 | 0.001 | 0.018 |
| rs7909331 | G | -0.016 | 0.003 | -0.015 | 0.013 | 0.008 | 0.018 | -0.004 | 0.014 | -0.006 | 0.022 | 0.001 | 0.009 | 0.008 | 0.018 | -0.002 | 0.012 | 0.016 | 0.016 | -0.04 | 0.022 | -0.012 | 0.021 |
| rs79269403 | A | 0.021 | 0.003 | 0.006 | 0.011 | -0.013 | 0.015 | 0.007 | 0.013 | -0.022 | 0.018 | -0.02 | 0.008 | -0.029 | 0.015 | -0.028 | 0.01 | -0.008 | 0.013 | 0.006 | 0.01 | -0.021 | 0.018 |
| rs7927195 | G | -0.015 | 0.002 | -0.017 | 0.01 | -0.029 | 0.014 | -0.023 | 0.011 | -0.026 | 0.017 | 0.001 | 0.007 | -0.019 | 0.013 | -0.001 | 0.008 | -0.008 | 0.012 | -0.002 | 0.011 | -0.021 | 0.016 |
| rs7942078 | T | 0.016 | 0.002 |  |  |  |  |  |  |  |  |  |  |  |  |  |  |  |  |  |  |  |  |
| rs794375 | C | 0.014 | 0.002 | -0.003 | 0.01 | 0.014 | 0.013 | -0.003 | 0.011 | 0.004 | 0.016 | -0.022 | 0.007 | -0.043 | 0.012 | -0.031 | 0.008 | -0.02 | 0.011 | 0.01 | 0.012 | -0.01 | 0.015 |
| rs7955865 | T | -0.013 | 0.002 |  |  |  |  |  |  |  |  |  |  |  |  |  |  |  |  |  |  |  |  |
| rs7972441 | A | -0.015 | 0.002 | 0.023 | 0.01 | -0.004 | 0.013 | 0.011 | 0.011 | 0.011 | 0.016 | -0.001 | 0.01 | 0.007 | 0.012 | 0.006 | 0.012 | 0.013 | 0.011 | 0.01 | 0.014 | 0.001 | 0.015 |
| rs803679 | A | 0.018 | 0.003 | -0.011 | 0.01 | -0.009 | 0.016 | -0.019 | 0.012 | -0.018 | 0.019 | -0.002 | 0.008 | 0.012 | 0.015 | 0.001 | 0.01 | -0.005 | 0.014 | -0.021 | 0.01 | -0.021 | 0.018 |
| rs807478 | G | 0.012 | 0.002 | 0.007 | 0.01 | 0.015 | 0.013 | 0.004 | 0.011 | 0.01 | 0.016 | 0.008 | 0.007 | 0.01 | 0.012 | 0.013 | 0.008 | 0.022 | 0.011 | 0.021 | 0.012 | 0.01 | 0.015 |
| rs809955 | A | 0.016 | 0.002 | 0.006 | 0.01 | -0.009 | 0.014 | 0.002 | 0.011 | -0.036 | 0.016 | 0 | 0.007 | 0.014 | 0.013 | -0.022 | 0.008 | -0.02 | 0.012 | -0.019 | 0.013 | -0.017 | 0.016 |
| rs8180995 | G | 0.012 | 0.002 | 0.003 | 0.009 | 0.001 | 0.013 | 0.003 | 0.01 | 0.001 | 0.016 | -0.001 | 0.007 | -0.018 | 0.012 | 0.002 | 0.008 | 0.005 | 0.011 | 0.016 | 0.014 | -0.002 | 0.015 |
| rs838042 | A | 0.016 | 0.002 | -0.005 | 0.01 | -0.011 | 0.013 | -0.009 | 0.011 | -0.013 | 0.016 | -0.005 | 0.007 | 0.004 | 0.013 | -0.01 | 0.009 | -0.013 | 0.011 | 0.01 | 0.011 | 0.009 | 0.015 |
| rs9403187 | T | 0.015 | 0.002 |  |  |  |  |  |  |  |  |  |  |  |  |  |  |  |  |  |  |  |  |
| rs9514600 | G | -0.011 | 0.002 |  |  |  |  |  |  |  |  |  |  |  |  |  |  |  |  |  |  |  |  |
| rs9536994 | C | 0.013 | 0.002 | 0.003 | 0.009 | 0.001 | 0.014 | 0.006 | 0.01 | 0.021 | 0.016 | 0.007 | 0.01 | -0.001 | 0.013 | 0.007 | 0.012 | 0.014 | 0.012 | 0.012 | 0.012 | 0.029 | 0.016 |
| rs9538248 | A | -0.016 | 0.002 | 0.006 | 0.011 | 0.013 | 0.014 | 0 | 0.012 | 0.026 | 0.017 | -0.01 | 0.007 | -0.017 | 0.014 | 0.01 | 0.008 | -0.009 | 0.012 | 0.016 | 0.012 | 0.012 | 0.017 |
| rs9581878 | A | 0.020 | 0.003 |  |  |  |  |  |  |  |  |  |  |  |  |  |  |  |  |  |  |  |  |
| rs9643087 | T | -0.014 | 0.002 | 0.007 | 0.009 | 0.011 | 0.013 | -0.005 | 0.01 | 0.016 | 0.016 | 0.009 | 0.007 | -0.014 | 0.012 | 0.016 | 0.009 | 0.002 | 0.011 | 0.02 | 0.022 | -0.008 | 0.015 |
| rs9809849 | A | -0.014 | 0.002 | 0.026 | 0.01 | -0.008 | 0.014 | 0.026 | 0.011 | -0.012 | 0.016 | 0.014 | 0.007 | -0.01 | 0.013 | -0.003 | 0.008 | 0.006 | 0.012 | 0 | 0.011 | -0.01 | 0.016 |
| rs9835772 | T | -0.014 | 0.002 |  |  |  |  |  |  |  |  |  |  |  |  |  |  |  |  |  |  |  |  |
| rs9923553 | G | -0.014 | 0.002 | -0.005 | 0.01 | -0.004 | 0.015 | -0.002 | 0.011 | 0.004 | 0.018 | -0.002 | 0.007 | 0.002 | 0.014 | 0.011 | 0.009 | 0.015 | 0.013 | 0.005 | 0.011 | -0.008 | 0.017 |
| rs993700 | C | 0.016 | 0.003 | -0.007 | 0.012 | -0.02 | 0.015 | -0.006 | 0.013 | -0.017 | 0.019 | -0.016 | 0.008 | -0.02 | 0.015 | -0.032 | 0.009 | -0.043 | 0.013 | -0.008 | 0.01 | -0.018 | 0.018 |

SNPs, Single-nucleotide polymorphiss. EA, effect allele. SE, standard error.

# Supplementary Table 4. Genome-wide significant SNPs for lifetime number of sexual partners.

| **SNP** | **CHR** | **Position** | **EA** | **OA** | **EAF** | **BETA** | **SE** | **P-value** | **N** | **F statistic** | **R^2^** |
| --- | --- | --- | --- | --- | --- | --- | --- | --- | --- | --- | --- |
| rs10763152 | 10 | 56646290 | A | G | 0.556 | -0.011 | 0.002 | 1.30E-08 | 378882 | 32.4 | 0.0001 |
| rs10774020 | 12 | 348876 | C | T | 0.666 | -0.012 | 0.002 | 1.30E-09 | 378882 | 36.8 | 0.0001 |
| rs10782302 | 6 | 100993125 | C | T | 0.495 | 0.012 | 0.002 | 1.40E-11 | 378882 | 45.7 | 0.0001 |
| rs10782490 | 14 | 98549383 | C | T | 0.469 | 0.015 | 0.002 | 7.70E-16 | 378882 | 64.9 | 0.0002 |
| rs10788954 | 1 | 53743677 | C | T | 0.502 | -0.012 | 0.002 | 1.40E-10 | 378882 | 41.1 | 0.0001 |
| rs11111212 | 12 | 102610368 | G | A | 0.217 | 0.013 | 0.002 | 1.50E-08 | 378882 | 32.0 | 0.0001 |
| rs11165696 | 1 | 97136327 | G | A | 0.177 | 0.015 | 0.002 | 4.30E-10 | 378882 | 39.0 | 0.0001 |
| rs11627454 | 14 | 48559491 | G | A | 0.223 | -0.013 | 0.002 | 2.40E-09 | 378882 | 35.6 | 0.0001 |
| rs11642841 | 16 | 53845487 | A | C | 0.397 | -0.011 | 0.002 | 1.70E-08 | 378882 | 31.8 | 0.0001 |
| rs117236391 | 18 | 40505274 | A | G | 0.023 | -0.036 | 0.006 | 1.80E-08 | 378882 | 31.7 | 0.0001 |
| rs11847782 | 14 | 41052541 | A | G | 0.377 | 0.010 | 0.002 | 3.40E-08 | 378882 | 30.5 | 0.0001 |
| rs12244388 | 10 | 104640052 | A | G | 0.336 | 0.015 | 0.002 | 5.50E-14 | 378882 | 56.5 | 0.0001 |
| rs12655983 | 5 | 45307926 | G | T | 0.133 | 0.015 | 0.003 | 1.70E-08 | 378882 | 31.8 | 0.0001 |
| rs12667600 | 7 | 1895755 | A | C | 0.394 | -0.011 | 0.002 | 1.40E-08 | 378882 | 32.2 | 0.0001 |
| rs13156646 | 5 | 164619355 | C | G | 0.372 | 0.011 | 0.002 | 3.30E-08 | 378882 | 30.5 | 0.0001 |
| rs1372626 | 18 | 49997807 | A | G | 0.594 | -0.011 | 0.002 | 1.10E-09 | 378882 | 37.2 | 0.0001 |
| rs1375601 | 1 | 204965657 | G | A | 0.584 | -0.011 | 0.002 | 9.40E-09 | 378882 | 33.0 | 0.0001 |
| rs1512224 | 2 | 60504939 | C | G | 0.587 | 0.014 | 0.002 | 4.80E-14 | 378882 | 56.8 | 0.0001 |
| rs1560660 | 5 | 166951962 | A | G | 0.647 | -0.012 | 0.002 | 1.00E-09 | 378882 | 37.2 | 0.0001 |
| rs162057 | 5 | 103965358 | G | T | 0.662 | 0.012 | 0.002 | 1.40E-09 | 378882 | 36.6 | 0.0001 |
| rs16884402 | 4 | 31141947 | C | T | 0.286 | -0.012 | 0.002 | 1.50E-08 | 378882 | 32.1 | 0.0001 |
| rs17690703 | 17 | 43925297 | T | C | 0.264 | -0.014 | 0.002 | 8.90E-12 | 378882 | 46.6 | 0.0001 |
| rs1986692 | 7 | 133743393 | G | A | 0.382 | -0.011 | 0.002 | 7.20E-09 | 378882 | 33.5 | 0.0001 |
| rs2035166 | 8 | 76749722 | G | A | 0.679 | 0.011 | 0.002 | 4.00E-08 | 378882 | 30.2 | 0.0001 |
| rs2119453 | 7 | 127286435 | A | T | 0.410 | 0.011 | 0.002 | 3.30E-09 | 378882 | 35.0 | 0.0001 |
| rs2186707 | 11 | 112850643 | A | T | 0.386 | 0.012 | 0.002 | 4.20E-10 | 378882 | 39.0 | 0.0001 |
| rs2194027 | 5 | 87822672 | A | T | 0.489 | 0.014 | 0.002 | 2.50E-14 | 378882 | 58.1 | 0.0002 |
| rs2279829 | 3 | 147106319 | T | C | 0.214 | -0.015 | 0.002 | 9.80E-11 | 378882 | 41.9 | 0.0001 |
| rs2281824 | 14 | 103800071 | T | C | 0.277 | -0.011 | 0.002 | 4.80E-08 | 378882 | 29.8 | 0.0001 |
| rs2341580 | 7 | 3588408 | C | T | 0.258 | -0.012 | 0.002 | 6.10E-09 | 378882 | 33.8 | 0.0001 |
| rs2422136 | 1 | 72618544 | A | C | 0.494 | 0.011 | 0.002 | 2.00E-09 | 378882 | 36.0 | 0.0001 |
| rs2726461 | 4 | 106185456 | T | C | 0.812 | -0.016 | 0.002 | 2.50E-11 | 378882 | 44.5 | 0.0001 |
| rs273512 | 19 | 18224729 | T | C | 0.404 | -0.012 | 0.002 | 5.70E-10 | 378882 | 38.4 | 0.0001 |
| rs28457046 | 3 | 127166397 | C | G | 0.432 | 0.011 | 0.002 | 3.60E-09 | 378882 | 34.8 | 0.0001 |
| rs28548534 | 3 | 174225252 | T | C | 0.251 | 0.012 | 0.002 | 2.40E-08 | 378882 | 31.1 | 0.0001 |
| rs2929289 | 8 | 9071845 | T | C | 0.766 | -0.013 | 0.002 | 4.20E-09 | 378882 | 34.5 | 0.0001 |
| rs2936831 | 12 | 120164264 | C | T | 0.531 | 0.011 | 0.002 | 3.00E-09 | 378882 | 35.2 | 0.0001 |
| rs34495106 | 3 | 85651136 | G | A | 0.628 | -0.014 | 0.002 | 2.40E-13 | 378882 | 53.7 | 0.0001 |
| rs35488407 | 1 | 243619130 | G | C | 0.160 | -0.014 | 0.003 | 4.30E-08 | 378882 | 30.0 | 0.0001 |
| rs36183315 | 16 | 17610970 | A | G | 0.212 | -0.013 | 0.002 | 4.10E-08 | 378882 | 30.1 | 0.0001 |
| rs3740484 | 10 | 102747363 | T | G | 0.331 | -0.011 | 0.002 | 2.40E-08 | 378882 | 31.1 | 0.0001 |
| rs4702 | 15 | 91426560 | A | G | 0.557 | -0.013 | 0.002 | 1.80E-12 | 378882 | 49.7 | 0.0001 |
| rs4869737 | 6 | 151892135 | T | C | 0.701 | 0.012 | 0.002 | 9.60E-09 | 378882 | 32.9 | 0.0001 |
| rs542391 | 4 | 130045700 | T | C | 0.902 | -0.019 | 0.003 | 9.10E-10 | 378882 | 37.5 | 0.0001 |
| rs55731932 | 10 | 134139482 | T | C | 0.326 | 0.011 | 0.002 | 9.40E-09 | 378882 | 33.0 | 0.0001 |
| rs55918828 | 5 | 152187782 | T | C | 0.285 | -0.013 | 0.002 | 6.90E-11 | 378882 | 42.6 | 0.0001 |
| rs56016607 | 18 | 53164762 | G | A | 0.054 | -0.030 | 0.004 | 3.10E-13 | 378882 | 53.1 | 0.0001 |
| rs58184964 | 2 | 22558876 | A | G | 0.387 | 0.012 | 0.002 | 2.10E-10 | 378882 | 40.4 | 0.0001 |
| rs62084701 | 17 | 66042238 | T | C | 0.748 | -0.013 | 0.002 | 2.80E-10 | 378882 | 39.8 | 0.0001 |
| rs62432515 | 6 | 100126102 | T | C | 0.054 | -0.024 | 0.004 | 7.60E-09 | 378882 | 33.4 | 0.0001 |
| rs62521080 | 8 | 65529350 | A | G | 0.102 | -0.022 | 0.003 | 2.90E-13 | 378882 | 53.3 | 0.0001 |
| rs631248 | 1 | 44071221 | A | G | 0.782 | -0.013 | 0.002 | 2.20E-09 | 378882 | 35.8 | 0.0001 |
| rs6721925 | 2 | 77333989 | T | A | 0.255 | 0.012 | 0.002 | 7.40E-09 | 378882 | 33.4 | 0.0001 |
| rs6723108 | 2 | 135479980 | T | G | 0.481 | 0.010 | 0.002 | 3.50E-08 | 378882 | 30.4 | 0.0001 |
| rs6908726 | 6 | 28671343 | C | G | 0.874 | 0.017 | 0.003 | 1.50E-09 | 378882 | 36.6 | 0.0001 |
| rs75120545 | 2 | 44271496 | T | C | 0.031 | -0.034 | 0.006 | 3.00E-09 | 378882 | 35.2 | 0.0001 |
| rs75974927 | 2 | 76486071 | C | T | 0.069 | 0.021 | 0.004 | 2.50E-08 | 378882 | 31.1 | 0.0001 |
| rs7783012 | 7 | 114116881 | A | G | 0.594 | 0.014 | 0.002 | 1.70E-13 | 378882 | 54.3 | 0.0001 |
| rs8063842 | 16 | 49561498 | A | G | 0.080 | 0.022 | 0.003 | 1.40E-10 | 378882 | 41.1 | 0.0001 |
| rs9641538 | 7 | 115031575 | A | G | 0.556 | -0.010 | 0.002 | 2.30E-08 | 378882 | 31.2 | 0.0001 |
| rs966971 | 2 | 60138262 | T | C | 0.585 | 0.012 | 0.002 | 4.60E-11 | 378882 | 43.3 | 0.0001 |
| rs9846870 | 3 | 117347142 | A | C | 0.838 | 0.015 | 0.003 | 9.90E-09 | 378882 | 32.9 | 0.0001 |
| rs9922596 | 16 | 72006370 | T | C | 0.221 | -0.014 | 0.002 | 6.00E-10 | 378882 | 38.3 | 0.0001 |

SNPs, Single-nucleotide polymorphiss. CHR, chromosome. EA, effect allele.EAF, effect allele frequency. SE, standard error. R², explained variation by SNPs.

**Supplementary Table 5**. Association of genome-wide significant SNPs for lifetime number of sexual partners with cardiovascular diseases.

| **Lifetime number of sexual partners** | | | | **Coronary artery disease** | | | | **Myocardial infarction** | | | | **Atrial fibrillation** | | | | **Heart failure** | | | | **Ischemic stroke** | | | |
| --- | --- | --- | --- | --- | --- | --- | --- | --- | --- | --- | --- | --- | --- | --- | --- | --- | --- | --- | --- | --- | --- | --- | --- |
| **Data sources: UKbiobank** | | | | **CARDIoGRAMplusC4D** | | **FinnGen** | | **CARDIoGRAMplusC4D** | | **FinnGen** | | **Nielsen et al** | | **FinnGen** | | **HERMES** | | **FinnGen** | | **MEGASTROKE** | | **FinnGen** | |
| SNP | EA | BETA | SE | BETA | SE | BETA | SE | BETA | SE | BETA | SE | BETA | SE | BETA | SE | BETA | SE | BETA | SE | BETA | SE | BETA | SE |
| rs10763152 | A | -0.011 | 0.002 | -0.002 | 0.009 | 0.000 | 0.013 | -0.002 | 0.010 | -0.008 | 0.016 | -0.008 | 0.007 | 0.005 | 0.013 | -0.011 | 0.008 | -0.001 | 0.011 | -0.008 | 0.010 | 0.001 | 0.015 |
| rs10774020 | C | -0.012 | 0.002 | 0.009 | 0.010 | -0.006 | 0.014 | 0.005 | 0.011 | 0.004 | 0.017 | 0.008 | 0.007 | 0.020 | 0.014 | 0.000 | 0.008 | -0.007 | 0.012 | -0.015 | 0.010 | 0.023 | 0.016 |
| rs10782302 | C | 0.012 | 0.002 | -0.012 | 0.009 | -0.007 | 0.013 | -0.001 | 0.010 | -0.001 | 0.016 | 0.003 | 0.007 | -0.014 | 0.012 | 0.016 | 0.008 | 0.008 | 0.011 | 0.008 | 0.013 | -0.008 | 0.015 |
| rs10782490 | C | 0.015 | 0.002 | 0.020 | 0.009 | 0.000 | 0.013 | 0.013 | 0.010 | -0.011 | 0.016 | 0.003 | 0.007 | -0.006 | 0.012 | 0.007 | 0.008 | -0.008 | 0.011 | -0.004 | 0.019 | 0.004 | 0.015 |
| rs10788954 | C | -0.012 | 0.002 | 0.001 | 0.009 | 0.024 | 0.013 | 0.000 | 0.010 | 0.019 | 0.016 | -0.008 | 0.007 | -0.009 | 0.012 | -0.009 | 0.008 | 0.018 | 0.011 | 0.011 | 0.010 | 0.014 | 0.015 |
| rs11111212 | G | 0.013 | 0.002 | 0.006 | 0.011 | 0.010 | 0.014 | 0.004 | 0.012 | 0.000 | 0.017 | 0.000 | 0.008 | -0.011 | 0.013 | -0.033 | 0.009 | -0.011 | 0.012 | -0.003 | 0.010 | -0.026 | 0.016 |
| rs11165696 | G | 0.015 | 0.002 | 0.003 | 0.012 | 0.044 | 0.016 | 0.014 | 0.013 | 0.055 | 0.020 | 0.000 | 0.009 | 0.002 | 0.016 | -0.001 | 0.010 | 0.000 | 0.014 | 0.021 | 0.014 | 0.022 | 0.019 |
| rs11627454 | G | -0.013 | 0.002 | 0.006 | 0.012 | -0.005 | 0.017 | -0.010 | 0.013 | -0.006 | 0.020 | 0.002 | 0.008 | 0.008 | 0.016 | -0.013 | 0.010 | -0.012 | 0.015 | 0.001 | 0.013 | -0.003 | 0.020 |
| rs11642841 | A | -0.011 | 0.002 | 0.021 | 0.010 | 0.015 | 0.013 | 0.015 | 0.011 | 0.035 | 0.016 | 0.030 | 0.007 | 0.026 | 0.013 | 0.038 | 0.008 | 0.032 | 0.011 | 0.011 | 0.010 | 0.018 | 0.015 |
| rs117236391 | A | -0.036 | 0.006 | 0.016 | 0.035 | -0.066 | 0.109 | 0.035 | 0.039 | -0.096 | 0.130 | 0.002 | 0.024 | -0.183 | 0.103 | -0.024 | 0.028 | -0.044 | 0.093 | 0.003 | 0.011 | -0.005 | 0.124 |
| rs11847782 | A | 0.010 | 0.002 | -0.006 | 0.009 | -0.006 | 0.013 | -0.007 | 0.010 | -0.006 | 0.016 | -0.005 | 0.007 | 0.000 | 0.013 | -0.003 | 0.008 | 0.002 | 0.012 | 0.017 | 0.011 | 0.007 | 0.016 |
| rs12244388 | A | 0.015 | 0.002 | -0.036 | 0.009 | -0.030 | 0.013 | -0.030 | 0.010 | -0.027 | 0.016 | 0.013 | 0.007 | 0.031 | 0.013 | -0.020 | 0.008 | -0.005 | 0.012 | 0.004 | 0.011 | 0.021 | 0.016 |
| rs12655983 | G | 0.015 | 0.003 | 0.007 | 0.012 | 0.001 | 0.018 | 0.003 | 0.014 | -0.001 | 0.022 | -0.010 | 0.010 | 0.004 | 0.018 | 0.004 | 0.012 | 0.028 | 0.016 | 0.003 | 0.013 | 0.020 | 0.021 |
| rs12667600 | A | -0.011 | 0.002 | -0.008 | 0.010 | -0.010 | 0.014 | -0.002 | 0.011 | -0.008 | 0.017 | 0.009 | 0.007 | -0.006 | 0.014 | 0.010 | 0.008 | -0.010 | 0.012 | 0.004 | 0.009 | 0.036 | 0.017 |
| rs13156646 | C | 0.011 | 0.002 | -0.006 | 0.009 |  |  | -0.010 | 0.010 |  |  | 0.001 | 0.007 |  |  |  |  |  |  | -0.005 | 0.012 |  |  |
| rs1372626 | A | -0.011 | 0.002 | -0.001 | 0.009 | 0.021 | 0.013 | 0.001 | 0.010 | 0.027 | 0.016 | -0.004 | 0.007 | 0.023 | 0.013 | 0.008 | 0.008 | -0.004 | 0.012 | 0.001 | 0.010 | 0.023 | 0.016 |
| rs1375601 | G | -0.011 | 0.002 | -0.014 | 0.010 | -0.022 | 0.013 | -0.004 | 0.011 | -0.011 | 0.016 | -0.003 | 0.007 | 0.006 | 0.013 | -0.006 | 0.008 | 0.003 | 0.011 | 0.006 | 0.011 | 0.011 | 0.015 |
| rs1512224 | C | 0.014 | 0.002 | -0.006 | 0.010 | -0.006 | 0.013 |  |  | -0.005 | 0.016 |  |  | 0.000 | 0.013 |  |  | -0.002 | 0.012 | 0.000 | 0.013 | 0.024 | 0.016 |
| rs1560660 | A | -0.012 | 0.002 | 0.013 | 0.010 | 0.026 | 0.013 | -0.001 | 0.011 | 0.031 | 0.016 | -0.008 | 0.007 | 0.014 | 0.013 | -0.005 | 0.008 | 0.007 | 0.012 | -0.010 | 0.012 | -0.014 | 0.016 |
| rs162057 | G | 0.012 | 0.002 | 0.005 | 0.011 | 0.014 | 0.014 | 0.017 | 0.012 | 0.009 | 0.016 | -0.002 | 0.007 | -0.002 | 0.013 | -0.003 | 0.008 | -0.003 | 0.012 | 0.008 | 0.010 | 0.013 | 0.016 |
| rs16884402 | C | -0.012 | 0.002 | 0.011 | 0.010 | -0.008 | 0.015 | 0.011 | 0.011 | 0.009 | 0.018 | 0.012 | 0.007 | -0.006 | 0.015 | 0.003 | 0.009 | 0.005 | 0.013 | 0.011 | 0.010 | 0.017 | 0.018 |
| rs17690703 | T | -0.014 | 0.002 | 0.039 | 0.014 | 0.022 | 0.020 | 0.042 | 0.015 | -0.004 | 0.024 | 0.035 | 0.008 | 0.053 | 0.019 | 0.011 | 0.010 | -0.015 | 0.017 | -0.002 | 0.012 | -0.002 | 0.023 |
| rs1986692 | G | -0.011 | 0.002 | -0.002 | 0.010 | -0.008 | 0.013 | -0.001 | 0.011 | -0.018 | 0.016 | -0.012 | 0.007 | -0.019 | 0.013 | 0.001 | 0.008 | -0.019 | 0.012 | 0.021 | 0.036 | 0.019 | 0.016 |
| rs2035166 | G | 0.011 | 0.002 | 0.013 | 0.010 | 0.007 | 0.014 | 0.023 | 0.011 | 0.007 | 0.016 | -0.002 | 0.007 | 0.001 | 0.013 | -0.020 | 0.008 | -0.011 | 0.012 | 0.038 | 0.011 | -0.029 | 0.016 |
| rs2119453 | A | 0.011 | 0.002 | -0.014 | 0.010 | 0.003 | 0.014 | 0.000 | 0.011 | 0.009 | 0.017 | 0.016 | 0.007 | 0.014 | 0.013 |  |  | -0.002 | 0.012 | -0.003 | 0.012 | -0.006 | 0.016 |
| rs2186707 | A | 0.012 | 0.002 |  |  | -0.020 | 0.014 |  |  | -0.012 | 0.016 | 0.003 | 0.007 | -0.013 | 0.013 |  |  | -0.027 | 0.012 | 0.013 | 0.010 | -0.003 | 0.016 |
| rs2194027 | A | 0.014 | 0.002 |  |  |  |  |  |  |  |  |  |  |  |  |  |  |  |  |  |  |  |  |
| rs2279829 | T | -0.015 | 0.002 | 0.010 | 0.011 | 0.000 | 0.014 | 0.018 | 0.012 | 0.003 | 0.017 | -0.010 | 0.008 | 0.005 | 0.014 | 0.010 | 0.009 | -0.002 | 0.012 | -0.004 | 0.010 | -0.016 | 0.017 |
| rs2281824 | T | -0.011 | 0.002 | 0.001 | 0.010 | 0.005 | 0.015 | 0.001 | 0.011 | -0.002 | 0.018 | 0.012 | 0.007 | 0.018 | 0.014 | -0.002 | 0.009 | 0.024 | 0.013 | -0.009 | 0.010 | 0.020 | 0.017 |
| rs2341580 | C | -0.012 | 0.002 | 0.014 | 0.011 | 0.034 | 0.016 | 0.011 | 0.012 | 0.025 | 0.019 | -0.008 | 0.008 | 0.016 | 0.015 | 0.003 | 0.009 | 0.006 | 0.014 | 0.008 | 0.012 | 0.009 | 0.019 |
| rs2422136 | A | 0.011 | 0.002 | -0.004 | 0.010 | -0.007 | 0.013 | -0.007 | 0.011 | -0.004 | 0.016 | 0.014 | 0.007 | 0.014 | 0.012 | -0.001 | 0.008 | 0.014 | 0.011 | 0.010 | 0.015 | 0.004 | 0.015 |
| rs2726461 | T | -0.016 | 0.002 | -0.008 | 0.011 | 0.004 | 0.017 | -0.024 | 0.012 | 0.024 | 0.020 | -0.003 | 0.008 | 0.025 | 0.016 | 0.003 | 0.010 | 0.009 | 0.015 | 0.005 | 0.011 | -0.011 | 0.020 |
| rs273512 | T | -0.012 | 0.002 | 0.001 | 0.010 | -0.033 | 0.013 | -0.011 | 0.011 | -0.026 | 0.016 | 0.009 | 0.007 | 0.021 | 0.012 | 0.015 | 0.008 | -0.002 | 0.011 | -0.003 | 0.011 | -0.001 | 0.015 |
| rs28457046 | C | 0.011 | 0.002 |  |  |  |  |  |  |  |  |  |  |  |  |  |  |  |  |  |  |  |  |
| rs28548534 | T | 0.012 | 0.002 | 0.007 | 0.011 | 0.008 | 0.015 | 0.008 | 0.012 | 0.008 | 0.018 | -0.001 | 0.008 | -0.012 | 0.014 | -0.015 | 0.009 | -0.006 | 0.013 | -0.021 | 0.011 | -0.024 | 0.017 |
| rs2929289 | T | -0.013 | 0.002 | -0.005 | 0.011 | 0.005 | 0.018 | 0.009 | 0.012 | 0.019 | 0.022 | 0.011 | 0.008 | -0.003 | 0.017 | -0.001 | 0.009 | -0.016 | 0.016 | 0.006 | 0.010 | -0.002 | 0.021 |
| rs2936831 | C | 0.011 | 0.002 | -0.005 | 0.009 | -0.006 | 0.013 | -0.005 | 0.010 | 0.019 | 0.016 | -0.005 | 0.007 | 0.002 | 0.013 | -0.010 | 0.008 | -0.009 | 0.011 | -0.005 | 0.011 | -0.008 | 0.015 |
| rs34495106 | G | -0.014 | 0.002 | 0.003 | 0.010 | 0.014 | 0.014 | -0.001 | 0.011 | 0.023 | 0.017 | -0.018 | 0.007 | -0.018 | 0.014 | -0.024 | 0.008 | -0.020 | 0.012 | -0.022 | 0.014 | 0.002 | 0.017 |
| rs35488407 | G | -0.014 | 0.003 | 0.001 | 0.014 | 0.012 | 0.018 | 0.006 | 0.015 | 0.019 | 0.022 | 0.015 | 0.009 | 0.003 | 0.017 |  |  | -0.020 | 0.016 | -0.006 | 0.015 | 0.030 | 0.021 |
| rs36183315 | A | -0.013 | 0.002 | -0.004 | 0.015 | 0.008 | 0.024 | -0.016 | 0.016 | 0.038 | 0.028 | -0.004 | 0.009 | 0.010 | 0.022 | -0.006 | 0.013 | -0.053 | 0.020 | 0.010 | 0.012 | -0.014 | 0.027 |
| rs3740484 | T | -0.011 | 0.002 | -0.005 | 0.010 | -0.013 | 0.015 | -0.008 | 0.011 | -0.027 | 0.018 | 0.009 | 0.007 | 0.005 | 0.014 | -0.003 | 0.008 | 0.002 | 0.013 | -0.022 | 0.012 | 0.017 | 0.017 |
| rs4702 | A | -0.013 | 0.002 | 0.038 | 0.010 | 0.053 | 0.013 | 0.043 | 0.012 | 0.037 | 0.016 | 0.005 | 0.007 | 0.024 | 0.012 | 0.012 | 0.008 | 0.008 | 0.011 | 0.060 | 0.014 | 0.039 | 0.015 |
| rs4869737 | T | 0.012 | 0.002 | 0.012 | 0.010 | -0.014 | 0.016 | 0.015 | 0.011 | -0.015 | 0.019 | -0.031 | 0.007 | 0.001 | 0.015 | -0.014 | 0.009 | 0.023 | 0.014 | -0.021 | 0.012 | 0.008 | 0.018 |
| rs542391 | T | -0.019 | 0.003 | -0.020 | 0.018 | 0.025 | 0.025 | -0.009 | 0.020 | 0.020 | 0.030 | -0.011 | 0.012 | -0.013 | 0.024 | 0.000 | 0.014 | 0.021 | 0.022 | -0.004 | 0.010 | 0.013 | 0.029 |
| rs55731932 | T | 0.011 | 0.002 | -0.027 | 0.010 | -0.002 | 0.015 | -0.014 | 0.012 | -0.011 | 0.018 | 0.010 | 0.007 | -0.003 | 0.014 | -0.006 | 0.008 | -0.033 | 0.013 | -0.024 | 0.011 | 0.035 | 0.017 |
| rs55918828 | T | -0.013 | 0.002 | -0.006 | 0.010 | -0.005 | 0.014 | -0.011 | 0.011 | 0.000 | 0.017 | 0.012 | 0.007 | 0.002 | 0.014 | 0.001 | 0.009 | -0.024 | 0.012 | -0.030 | 0.011 | -0.009 | 0.017 |
| rs56016607 | G | -0.030 | 0.004 | -0.030 | 0.024 | 0.005 | 0.032 | -0.038 | 0.026 | 0.042 | 0.039 | -0.035 | 0.015 | -0.041 | 0.031 | -0.008 | 0.017 | 0.013 | 0.028 | 0.000 | 0.011 | -0.020 | 0.038 |
| rs58184964 | A | 0.012 | 0.002 | 0.020 | 0.009 | 0.007 | 0.013 | 0.019 | 0.010 | 0.009 | 0.016 | 0.016 | 0.007 | -0.005 | 0.013 | 0.021 | 0.008 | -0.012 | 0.011 | -0.006 | 0.011 | -0.007 | 0.015 |
| rs62084701 | T | -0.013 | 0.002 | -0.022 | 0.012 | 0.007 | 0.014 | -0.022 | 0.013 | 0.022 | 0.017 | 0.002 | 0.008 | 0.015 | 0.014 | -0.010 | 0.010 | -0.021 | 0.012 | -0.012 | 0.012 | -0.012 | 0.017 |
| rs62432515 | T | -0.024 | 0.004 | -0.007 | 0.023 | -0.046 | 0.035 | 0.000 | 0.025 | -0.032 | 0.042 | 0.001 | 0.015 | -0.057 | 0.033 | -0.013 | 0.018 | 0.012 | 0.030 | -0.001 | 0.011 | 0.025 | 0.040 |
| rs62521080 | A | -0.022 | 0.003 | 0.019 | 0.017 | 0.019 | 0.021 | 0.009 | 0.019 | 0.007 | 0.026 | 0.019 | 0.011 | -0.007 | 0.020 | 0.019 | 0.013 | 0.023 | 0.018 | 0.012 | 0.011 | 0.022 | 0.025 |
| rs631248 | A | -0.013 | 0.002 | -0.009 | 0.011 | -0.002 | 0.016 | -0.016 | 0.012 | -0.018 | 0.019 | -0.007 | 0.008 | 0.028 | 0.015 | 0.006 | 0.010 | 0.007 | 0.014 | -0.001 | 0.010 | -0.055 | 0.019 |
| rs6721925 | T | 0.012 | 0.002 | -0.002 | 0.011 | 0.000 | 0.014 | -0.012 | 0.012 | -0.002 | 0.017 | 0.009 | 0.008 | 0.015 | 0.014 |  |  | -0.006 | 0.012 | -0.008 | 0.011 | 0.016 | 0.016 |
| rs6723108 | T | 0.010 | 0.002 | 0.006 | 0.011 | -0.018 | 0.013 | 0.003 | 0.012 | -0.038 | 0.016 | 0.000 | 0.007 | -0.007 | 0.012 | 0.001 | 0.008 | -0.022 | 0.011 | -0.003 | 0.011 | 0.017 | 0.015 |
| rs6908726 | C | 0.017 | 0.003 |  |  | -0.017 | 0.030 |  |  | -0.033 | 0.036 | 0.012 | 0.012 | -0.035 | 0.028 |  |  | -0.035 | 0.026 | -0.011 | 0.010 | -0.087 | 0.035 |
| rs75120545 | T | -0.034 | 0.006 | -0.024 | 0.028 | 0.017 | 0.031 | -0.025 | 0.031 | 0.053 | 0.037 | 0.013 | 0.021 | 0.012 | 0.030 | -0.018 | 0.024 | 0.010 | 0.027 | 0.002 | 0.011 | -0.003 | 0.036 |
| rs75974927 | C | 0.021 | 0.004 | -0.014 | 0.022 | -0.025 | 0.026 | -0.014 | 0.023 | -0.035 | 0.031 | -0.005 | 0.013 | -0.019 | 0.025 | -0.003 | 0.015 | -0.007 | 0.023 | 0.023 | 0.011 | -0.074 | 0.030 |
| rs7783012 | A | 0.014 | 0.002 | 0.020 | 0.009 | 0.031 | 0.013 | 0.009 | 0.010 | 0.031 | 0.016 | 0.002 | 0.007 | 0.007 | 0.013 | -0.001 | 0.008 | 0.015 | 0.011 | -0.017 | 0.015 | 0.007 | 0.015 |
| rs8063842 | A | 0.022 | 0.003 | 0.014 | 0.017 | -0.020 | 0.018 | 0.036 | 0.019 | -0.035 | 0.021 | 0.005 | 0.011 | 0.021 | 0.017 | 0.005 | 0.013 | 0.014 | 0.015 | -0.007 | 0.010 | 0.019 | 0.020 |
| rs9641538 | A | -0.010 | 0.002 | -0.010 | 0.009 | -0.005 | 0.013 | -0.009 | 0.010 | -0.005 | 0.016 | 0.004 | 0.007 | -0.015 | 0.012 | 0.005 | 0.008 | -0.011 | 0.011 | 0.007 | 0.010 | -0.012 | 0.015 |
| rs966971 | T | 0.012 | 0.002 | 0.008 | 0.010 | -0.008 | 0.014 | 0.018 | 0.010 | -0.003 | 0.017 | 0.002 | 0.007 | -0.015 | 0.013 | -0.004 | 0.008 | 0.003 | 0.012 | -0.008 | 0.012 | 0.025 | 0.016 |
| rs9846870 | A | 0.015 | 0.003 | 0.018 | 0.013 | -0.006 | 0.015 | 0.025 | 0.014 | 0.010 | 0.018 | -0.002 | 0.009 | -0.011 | 0.015 | -0.001 | 0.010 | -0.004 | 0.013 | 0.021 | 0.011 | 0.010 | 0.018 |
| rs9922596 | T | -0.014 | 0.002 | -0.004 | 0.011 | -0.004 | 0.015 | -0.001 | 0.012 | -0.009 | 0.018 | 0.001 | 0.008 | -0.018 | 0.014 | -0.002 | 0.010 | -0.012 | 0.013 | -0.015 | 0.012 | 0.023 | 0.017 |

SNPs, Single-nucleotide polymorphiss.EA, effect allele.SE, standard error.

**Supplementary Table 6.** Evidence of association (p<5*10^-6^) of the SNPs used as genetic variants for Mendelian randomization analyses of age at first sexual intercourse with confounders or CVDs in the PhenoScanner.

| **SNP** | **CHR** | **Position** | **PhenoScanner traits linked to this gene** | **Excluded from complementary analysis** |
| --- | --- | --- | --- | --- |
| rs10104523 | 8 | 73889570 | NA | NO |
| rs10144067 | 14 | 93885198 | Body mass index(7.923e-16,UKBB) | YES |
| rs10469020 | 18 | 50811573 | NA | NO |
| rs10516875 | 4 | 91590266 | NA | NO |
| rs10746578 | 9 | 81512820 | NA | NO |
| rs10749233 | 10 | 118777998 | Body mass index(2.754e-11,UKBB) | YES |
| rs10853981 | 19 | 4965064 | Systolic blood pressure(3.614e-06,UKBB) | YES |
| rs10858054 | 1 | 115338190 | NA | NO |
| rs10871582 | 18 | 53276589 | Current tobacco smoking(4.63e-06,UKBB) | YES |
| rs10880086 | 12 | 41905017 | NA | NO |
| rs10922907 | 1 | 91193049 | NA | NO |
| rs10955084 | 8 | 97825208 | NA | NO |
| rs10978435 | 9 | 108949159 | NA | NO |
| rs10992812 | 9 | 96392182 | NA | NO |
| rs11240331 | 1 | 204968339 | NA | NO |
| rs112523595 | 3 | 85650341 | NA | NO |
| rs112633616 | 12 | 23238326 | NA | NO |
| rs112880127 | 10 | 120007757 | NA | NO |
| rs113247159 | 7 | 140182514 | NA | NO |
| rs113338260 | 5 | 46004640 | NA | NO |
| rs1156981 | 1 | 88829969 | NA | NO |
| rs11678980 | 2 | 162101261 | NA | NO |
| rs11688027 | 2 | 78018164 | NA | NO |
| rs11729080 | 4 | 112503872 | Past tobacco smoking(2.162e-06,UKBB) | YES |
| rs117831144 | 20 | 14731057 | NA | NO |
| rs11866420 | 16 | 90054704 | NA | NO |
| rs12049116 | 1 | 151050283 | Body mass index(1.015e-09,UKBB) | YES |
| rs12147463 | 14 | 41059928 | NA | NO |
| rs12203592 | 6 | 396321 | NA | NO |
| rs12204714 | 6 | 152235339 | NA | NO |
| rs12244388 | 10 | 104640052 | Body mass index(2.661e-06,28892062);Current tobacco smoking(1.375e-06,UKBB);Past tobacco smoking(3.191e-13,UKBB);Systolic blood pressure(4.276e-08,UKBB) | YES |
| rs1226414 | 2 | 157109930 | NA | NO |
| rs12292980 | 11 | 27734349 | Body mass index(2.074e-33,UKBB) | YES |
| rs12376530 | 9 | 14529836 | NA | NO |
| rs12448731 | 16 | 49622284 | NA | NO |
| rs12463727 | 2 | 26948413 | Body mass index(4.327e-07,28892062) | YES |
| rs12511982 | 4 | 60736871 | NA | NO |
| rs12523097 | 5 | 167411400 | NA | NO |
| rs12523398 | 5 | 45119647 | NA | NO |
| rs12554512 | 9 | 23352293 | NA | NO |
| rs12653396 | 5 | 87847273 | NA | NO |
| rs12714592 | 3 | 84387950 | Ever smoked(1.521e-10,UKBB) | YES |
| rs12714702 | 3 | 88249922 | Body mass index(6.621e-07,UKBB) | YES |
| rs12757779 | 1 | 232581436 | NA | NO |
| rs12878359 | 14 | 58816212 | NA | NO |
| rs12907546 | 15 | 47684280 | Current tobacco smoking(5.554e-08,UKBB) | YES |
| rs13175535 | 5 | 31078814 | NA | NO |
| rs13178956 | 5 | 154786457 | NA | NO |
| rs1320138 | 2 | 144158287 | NA | NO |
| rs1320330 | 2 | 622225 | Body mass index(1.572e-16,28892062);Past tobacco smoking(1.973e-06,UKBB);Systolic blood pressure(1.115e-06,UKBB) | YES |
| rs13280592 | 8 | 116686752 | Body mass index(4.657e-06,UKBB) | YES |
| rs13307225 | 7 | 105084671 | NA | NO |
| rs13394795 | 2 | 202867731 | NA | NO |
| rs1368546 | 2 | 104057364 | Past tobacco smoking(1.231e-12,UKBB) | YES |
| rs1392816 | 1 | 66481188 | Body mass index(2.738e-07,UKBB) | YES |
| rs141547796 | 6 | 50615935 | Body mass index(8.871e-11,UKBB) | YES |
| rs147633738 | 1 | 156320524 | Body mass index(1.001e-07,UKBB) | YES |
| rs147725178 | 2 | 185529344 | NA | NO |
| rs1547351 | 8 | 36842153 | NA | NO |
| rs1585634 | 8 | 54396376 | NA | NO |
| rs1609598 | 20 | 51510926 | NA | NO |
| rs1866710 | 11 | 12875312 | NA | NO |
| rs186723454 | 3 | 54156598 | NA | NO |
| rs191242857 | 9 | 127870796 | NA | NO |
| rs1925686 | 6 | 87858691 | NA | NO |
| rs1931263 | 1 | 96175101 | NA | NO |
| rs1962545 | 1 | 7522336 | NA | NO |
| rs1976423 | 5 | 104042643 | Body mass index(1.275e-06,UKBB) | YES |
| rs1991651 | 8 | 10706411 | Body mass index(9.042e-17,UKBB);Diastolic blood pressure(1.384e-13,UKBB);Systolic blood pressure(1.005e-12,UKBB) | YES |
| rs1995181 | 12 | 24195048 | NA | NO |
| rs2084572 | 3 | 17315758 | NA | NO |
| rs2091377 | 2 | 6145158 | Body mass index(3.39e-09,UKBB) | YES |
| rs2093623 | 10 | 10922977 | NA | NO |
| rs2130894 | 10 | 134039242 | NA | NO |
| rs215639 | 7 | 32373639 | Body mass index(2.959e-08,UKBB) | YES |
| rs2174752 | 13 | 69332015 | NA | NO |
| rs2176337 | 9 | 108959330 | NA | NO |
| rs2188151 | 3 | 50201924 | Body mass index(1.203e-33,UKBB) | YES |
| rs222440 | 6 | 52946320 | NA | NO |
| rs2274568 | 1 | 110612925 | NA | NO |
| rs2279574 | 12 | 89745477 | Body mass index(1.212e-10,UKBB) | YES |
| rs2406374 | 5 | 106936435 | NA | NO |
| rs2612030 | 3 | 53773437 | NA | NO |
| rs2650705 | 10 | 63239803 | NA | NO |
| rs28406364 | 17 | 47454507 | Diastolic blood pressure(1.791e-11,UKBB);Coronary artery disease(9.544e-13,29212778) | YES |
| rs28929474 | 14 | 94844947 | Diastolic blood pressure(2.173e-07,UKBB);Systolic blood pressure(1.455e-06,UKBB);Coronary artery disease(9.349e-09,29212778) | YES |
| rs2974311 | 8 | 42455166 | NA | NO |
| rs3007104 | 14 | 47367434 | Body mass index(1.471e-09,UKBB) | YES |
| rs341521 | 13 | 60399045 | NA | NO |
| rs34155040 | 18 | 44797697 | NA | NO |
| rs3447 | 9 | 86327243 | NA | NO |
| rs34517439 | 1 | 78450517 | Body mass index(6.401e-21,UKBB) | YES |
| rs34606772 | 3 | 24908376 | NA | NO |
| rs34804222 | 11 | 43771084 | Body mass index(8.621e-08,UKBB) | YES |
| rs34811474 | 4 | 25408838 | Body mass index(4.4e-20,29273807) | YES |
| rs35077383 | 1 | 151754112 | NA | NO |
| rs35851551 | 7 | 31330785 | NA | NO |
| rs359271 | 2 | 60463149 | NA | NO |
| rs3866460 | 6 | 152139943 | NA | NO |
| rs3896224 | 10 | 106467853 | Current tobacco smoking(4.669e-06,UKBB) | YES |
| rs410520 | 17 | 4938924 | NA | NO |
| rs4246175 | 9 | 134930808 | Body mass index(2.864e-07,UKBB) | YES |
| rs435538 | 5 | 24921398 | NA | NO |
| rs4439537 | 11 | 79887549 | NA | NO |
| rs4557006 | 2 | 22443840 | NA | NO |
| rs4569188 | 14 | 93915929 | Body mass index(2.407e-11,UKBB) | YES |
| rs4602427 | 3 | 117474457 | NA | NO |
| rs4702 | 15 | 91426560 | Diastolic blood pressure(2.358e-08,UKBB);Systolic blood pressure(1.023e-14,UKBB);Coronary artery disease(2.069e-14,29212778) | YES |
| rs4709807 | 6 | 164432799 | NA | NO |
| rs4728298 | 7 | 133436768 | NA | NO |
| rs4800204 | 18 | 22647270 | NA | NO |
| rs4809230 | 20 | 62439274 | NA | NO |
| rs4868800 | 5 | 166985224 | Past tobacco smoking(1.119e-06,UKBB);Smoking status: current(5.107e-11,UKBB) | YES |
| rs4952343 | 2 | 32858637 | NA | NO |
| rs4961705 | 9 | 16347927 | NA | NO |
| rs56306056 | 2 | 184416610 | NA | NO |
| rs56392241 | 3 | 131968209 | NA | NO |
| rs58938116 | 7 | 121946082 | NA | NO |
| rs590414 | 11 | 105746052 | NA | NO |
| rs592278 | 18 | 40231833 | NA | NO |
| rs6058613 | 20 | 30864279 | NA | NO |
| rs60775983 | 5 | 124204071 | Body mass index(5.481e-07,28892062) | YES |
| rs61856978 | 10 | 97941022 | NA | NO |
| rs62134195 | 2 | 45062249 | NA | NO |
| rs62177795 | 2 | 63475640 | NA | NO |
| rs62439690 | 7 | 21417556 | NA | NO |
| rs6504551 | 17 | 65903326 | Body mass index(7.974e-09,28892062) | YES |
| rs6517512 | 21 | 40512129 | NA | NO |
| rs6564268 | 16 | 75606878 | NA | NO |
| rs6586405 | 1 | 234739101 | NA | NO |
| rs6719762 | 2 | 60166832 | NA | NO |
| rs6744794 | 2 | 44842145 | NA | NO |
| rs6747099 | 2 | 60777498 | NA | NO |
| rs6748341 | 2 | 225377574 | NA | NO |
| rs67723420 | 3 | 35775115 | Body mass index(6.939e-09,UKBB) | YES |
| rs6776937 | 3 | 60870307 | NA | NO |
| rs6966769 | 7 | 1299334 | NA | NO |
| rs6978112 | 7 | 1966841 | Systolic blood pressure(4.119e-06,UKBB) | YES |
| rs7008955 | 8 | 26334103 | NA | NO |
| rs702 | 4 | 28710551 | NA | NO |
| rs705240 | 3 | 118457615 | NA | NO |
| rs7079070 | 10 | 134182921 | NA | NO |
| rs7091634 | 10 | 9973015 | NA | NO |
| rs7110863 | 11 | 112843138 | Body mass index in females(4.594e-07,25673413);Current tobacco smoking(3.745e-09,UKBB) | YES |
| rs714393 | 2 | 212698718 | NA | NO |
| rs7151954 | 14 | 103323448 | Body mass index(9.942e-11,UKBB) | YES |
| rs7152323 | 14 | 98553482 | Systolic blood pressure(2.584e-06,UKBB) | YES |
| rs7167444 | 15 | 97495941 | Ever smoked(3.034e-06,UKBB);Past tobacco smoking(3.929e-06,UKBB) | YES |
| rs7188873 | 16 | 24727064 | Body mass index(1.429e-07,UKBB);Systolic blood pressure(1.467e-07,UKBB) | YES |
| rs7201310 | 16 | 12513797 | NA | NO |
| rs7236339 | 18 | 77579773 | NA | NO |
| rs72674824 | 8 | 95489281 | Body mass index(2.874e-08,UKBB) | YES |
| rs72887338 | 6 | 67536056 | Ever smoked(4.786e-07,UKBB);Past tobacco smoking(6.955e-07,UKBB) | YES |
| rs7381195 | 5 | 60030791 | NA | NO |
| rs7452074 | 6 | 125050733 | NA | NO |
| rs74737734 | 14 | 30726670 | NA | NO |
| rs7476 | 11 | 46342834 | NA | NO |
| rs7503604 | 17 | 79095629 | NA | NO |
| rs7525548 | 1 | 75001474 | Past tobacco smoking(1.155e-07,UKBB) | YES |
| rs7566527 | 2 | 100811970 | Body mass index(1.51e-12,UKBB) | YES |
| rs7575189 | 2 | 174015168 | NA | NO |
| rs7618715 | 3 | 70873278 | NA | NO |
| rs763053 | 16 | 735921 | Ever smoked(1.597e-07,UKBB);Past tobacco smoking(1.887e-06,UKBB) | YES |
| rs76513770 | 16 | 72505534 | NA | NO |
| rs766406 | 6 | 26319588 | NA | NO |
| rs7671317 | 4 | 62972597 | NA | NO |
| rs767943 | 6 | 23446691 | NA | NO |
| rs7704530 | 5 | 30845465 | Diastolic blood pressure(1.1e-07,20838585) | YES |
| rs7783012 | 7 | 114116881 | Current tobacco smoking(2.067e-06,UKBB) | YES |
| rs7785195 | 7 | 3424686 | Ever smoked(4.13e-07,UKBB);Past tobacco smoking(9.502e-08,UKBB) | YES |
| rs7804551 | 7 | 99119110 | Body mass index(1.719e-12,UKBB);Past tobacco smoking(8.227e-07,UKBB) | YES |
| rs7815125 | 8 | 87680112 | Body mass index(4.854e-07,UKBB) | YES |
| rs7824756 | 8 | 51118559 | NA | NO |
| rs783544 | 15 | 83240293 | NA | NO |
| rs7909331 | 10 | 11205224 | NA | NO |
| rs79269403 | 3 | 108036819 | NA | NO |
| rs7927195 | 11 | 127478073 | Past tobacco smoking(2.117e-06,UKBB) | YES |
| rs7942078 | 11 | 28656064 | Body mass index(1.054e-08,28892062) | YES |
| rs794375 | 7 | 75147801 | Body mass index(1.43e-22,UKBB);Diastolic blood pressure(2.328e-07,UKBB);Systolic blood pressure(9.071e-07,UKBB); | YES |
| rs7955865 | 12 | 56468706 | NA | NO |
| rs7972441 | 12 | 84043146 | NA | NO |
| rs803679 | 1 | 44349405 | NA | NO |
| rs807478 | 19 | 36252494 | NA | NO |
| rs809955 | 4 | 140874760 | Body mass index(1.193e-09,UKBB) | YES |
| rs8180995 | 8 | 143326237 | NA | NO |
| rs838042 | 2 | 140320186 | NA | NO |
| rs9403187 | 6 | 100324813 | NA | NO |
| rs9514600 | 13 | 107644422 | NA | NO |
| rs9536994 | 13 | 55757388 | NA | NO |
| rs9538248 | 13 | 59492828 | NA | NO |
| rs9581878 | 13 | 28104552 | NA | NO |
| rs9643087 | 8 | 115382484 | NA | NO |
| rs9809849 | 3 | 3726156 | NA | NO |
| rs9835772 | 3 | 85766025 | Past tobacco smoking(1.742e-08,UKBB) | YES |
| rs9923553 | 16 | 5825579 | NA | NO |
| rs993700 | 4 | 67825894 | NA | NO |

SNPs, Single-nucleotide polymorphiss. CHR, chromosome.

**Supplementary Table 7.** Evidence of association (p<5*10^-6^) of the SNPs used as genetic variants for Mendelian randomization analyses of lifetime number of sexual partners with confounders or CVDs in the PhenoScanner.

| **SNP** | **CHR** | **Position** | **PhenoScanner traits linked to this gene** | **Excluded from complementary analysis** |
| --- | --- | --- | --- | --- |
| rs10763152 | 10 | 56646290 | Body mass index(3.372e-10,23754948) | YES |
| rs10774020 | 12 | 348876 | NA | NO |
| rs10782302 | 6 | 100993125 | NA | NO |
| rs10782490 | 14 | 98549383 | NA | NO |
| rs10788954 | 1 | 53743677 | NA | NO |
| rs11111212 | 12 | 102610368 | NA | NO |
| rs11165696 | 1 | 97136327 | NA | NO |
| rs11627454 | 14 | 48559491 | NA | NO |
| rs11642841 | 16 | 53845487 | Ever smoked(1.075e-07,UKBB);Past tobacco smoking(8.555e-08,UKBB) | YES |
| rs117236391 | 18 | 40505274 | NA | NO |
| rs11847782 | 14 | 41052541 | NA | NO |
| rs12244388 | 10 | 104640052 | Type 2 diabetes(5.36e-10,23209189);Diastolic blood pressure(9.204e-07,UKBB);Systolic blood pressure(4.682e-07,UKBB) | YES |
| rs12655983 | 5 | 45307926 | NA | NO |
| rs12667600 | 7 | 1895755 | Ever smoked(2.756e-14,UKBB) | YES |
| rs13156646 | 5 | 164619355 | NA | NO |
| rs1372626 | 18 | 49997807 | NA | NO |
| rs1375601 | 1 | 204965657 | NA | NO |
| rs1512224 | 2 | 60504939 | NA | NO |
| rs1560660 | 5 | 166951962 | NA | NO |
| rs162057 | 5 | 103965358 | Ever smoked(2.016e-06,UKBB) | YES |
| rs16884402 | 4 | 31141947 | NA | NO |
| rs17690703 | 17 | 43925297 | NA | NO |
| rs1986692 | 7 | 133743393 | NA | NO |
| rs2035166 | 8 | 76749722 | NA | NO |
| rs2119453 | 7 | 127286435 | NA | NO |
| rs2186707 | 11 | 112850643 | NA | NO |
| rs2194027 | 5 | 87822672 | NA | NO |
| rs2279829 | 3 | 147106319 | NA | NO |
| rs2281824 | 14 | 103800071 | Diastolic blood pressure(2.653e-10,UKBB);Ever smoked(1.696e-06,UKBB) | YES |
| rs2341580 | 7 | 3588408 | NA | NO |
| rs2422136 | 1 | 72618544 | NA | NO |
| rs2726461 | 4 | 106185456 | NA | NO |
| rs273512 | 19 | 18224729 | Current tobacco smoking(2.067e-06,UKBB) | YES |
| rs28457046 | 3 | 127166397 | NA | NO |
| rs28548534 | 3 | 174225252 | NA | NO |
| rs2929289 | 8 | 9071845 | Current tobacco smoking(2.396e-08,UKBB) | YES |
| rs2936831 | 12 | 120164264 | NA | NO |
| rs34495106 | 3 | 85651136 | Body mass index(2.777e-06,UKBB) | YES |
| rs35488407 | 1 | 243619130 | NA | NO |
| rs36183315 | 16 | 17610970 | NA | NO |
| rs3740484 | 10 | 102747363 | Body mass index(2.661e-06,28892062);Current tobacco smoking(1.375e-06,UKBB) | YES |
| rs4702 | 15 | 91426560 | NA | NO |
| rs4869737 | 6 | 151892135 | NA | NO |
| rs542391 | 4 | 130045700 | NA | NO |
| rs55731932 | 10 | 134139482 | Body mass index in females(8.883e-07,25673413);Current tobacco smoking(4.525e-09,UKBB);Diastolic blood pressure(4.229e-06,UKBB) | YES |
| rs55918828 | 5 | 152187782 | NA | NO |
| rs56016607 | 18 | 53164762 | NA | NO |
| rs58184964 | 2 | 22558876 | NA | NO |
| rs62084701 | 17 | 66042238 | NA | NO |
| rs62432515 | 6 | 100126102 | NA | NO |
| rs62521080 | 8 | 65529350 | NA | NO |
| rs631248 | 1 | 44071221 | NA | NO |
| rs6721925 | 2 | 77333989 | Diastolic blood pressure(2.358e-08,UKBB);Systolic blood pressure(1.023e-14,UKBB);Coronary artery disease(2.069e-14,29212778) | YES |
| rs6723108 | 2 | 135479980 | NA | NO |
| rs6908726 | 6 | 28671343 | NA | NO |
| rs75120545 | 2 | 44271496 | Body mass index(6.312e-07,UKBB) | YES |
| rs75974927 | 2 | 76486071 | Body mass index(7.687e-32,28892062);Type II diabetes(1.5e-16,26551672) | YES |
| rs7783012 | 7 | 114116881 | Body mass index(2.197e-08,UKBB) | YES |
| rs8063842 | 16 | 49561498 | Systolic blood pressure(4.188e-08,UKBB) | YES |
| rs9641538 | 7 | 115031575 | NA | NO |
| rs966971 | 2 | 60138262 | NA | NO |
| rs9846870 | 3 | 117347142 | Ever smoked(1.716e-08,UKBB) | YES |
| rs9922596 | 16 | 72006370 | Body mass index(4.498e-14,UKBB);Diastolic blood pressure(4.603e-07,UKBB) | YES |

SNPs, Single-nucleotide polymorphiss. CHR, chromosome.

# **Supplementary** Table 8. Heterogeneity for the Mendelian randomization analysis.

| **Exposures** | **Outcomes** | **SNP selection*** | **Cochran's Q** | **Degrees of Freedom** | **P-value** |
| --- | --- | --- | --- | --- | --- |
| Age at first sexual intercourse | Coronary artery disease | All | 263.8938618 | 154 | 8.59E-08 |
|  |  | Removed | 129.9893247 | 106 | 0.056767079 |
|  | Myocardial infarction | All | 231.9919631 | 154 | 4.90E-05 |
|  |  | Removed | 117.2172142 | 106 | 0.214703585 |
|  | Atrial fibrillation | All | 275.5972372 | 155 | 8.56E-09 |
|  |  | Removed | 176.2142503 | 106 | 2.20E-05 |
|  | Heart failure | All | 245.9954945 | 157 | 7.24E-06 |
|  |  | Removed | 133.2285972 | 107 | 0.043715141 |
|  | Ischemic stroke | All | 164.2331549 | 157 | 0.330121882 |
|  |  | Removed | 101.6858013 | 107 | 0.626910498 |
| Lifetime number of sexual partners | Coronary artery disease | All | 96.08039719 | 58 | 0.001237058 |
|  |  | Removed | 43.23152835 | 42 | 0.41850286 |
|  | Myocardial infarction | All | 83.0543215 | 57 | 0.013745614 |
|  |  | Removed | 38.65656478 | 42 | 0.618537402 |
|  | Atrial fibrillation | All | 128.4348352 | 59 | 4.59E-07 |
|  |  | Removed | 67.03282622 | 42 | 0.008356547 |
|  | Heart failure | All | 95.33571733 | 53 | 0.000323924 |
|  |  | Removed | 51.25980119 | 38 | 0.073845 |
|  | Ischemic stroke | All | 74.59416752 | 60 | 0.097291906 |
|  |  | Removed | 53.79255304 | 42 | 0.104911177 |

SNP, Single-nucleotide polymorphis.

* ‘All’ represents analyses with all selected SNPs, ‘Removed’ represents analyses after removing SNPs associated with relevant confounders or CVDs.

**Supplementary Table 9.** Sample size and priori power calculations in Mendelian randomization study of sexual factors and risk of cardiovascular disease.

| Outcomes | cases | controls | Sample size | R² | OR=0.90 | OR=0.85 | OR=0.80 | OR=0.75 | OR=0.70 | OR=0.65 |
| --- | --- | --- | --- | --- | --- | --- | --- | --- | --- | --- |
| Age at first sexual intercourse | | | | | | | | | | |
| Coronary artery disease | 60801 | 123504 | 184305 | 0.022317076 | 0.88 | 1 | - | - | - | - |
| Myocardial infarction | 43676 | 128197 | 171873 | 0.022317076 | 0.79 | 0.99 | 1 | - | - | - |
| Atrial fibrillation | 60620 | 970216 | 1030836 | 0.022317076 | 0.95 | 1 | - | - | - | - |
| Heart failure | 47309 | 930014 | 977323 | 0.022317076 | 0.89 | 1 | - | - | - | - |
| Ischemic stroke | 34217 | 406111 | 440328 | 0.022317076 | 0.76 | 0.98 | 1 | - | - | - |
| Lifetime number of sexual partners | | | | | | | | | | |
| Coronary artery disease | 60801 | 123504 | 184305 | 0.006365871 | 0.42 | 0.72 | 0.93 | 0.99 | 1 | - |
| Myocardial infarction | 43676 | 128197 | 171873 | 0.006365871 | 0.32 | 0.61 | 0.86 | 0.97 | 1 | - |
| Atrial fibrillation | 60620 | 970216 | 1030836 | 0.006365871 | 0.48 | 0.82 | 0.97 | 1 | - | - |
| Heart failure | 47309 | 930014 | 977323 | 0.006365871 | 0.4 | 0.73 | 0.93 | 0.99 | 1 | - |
| Ischemic stroke | 34217 | 406111 | 440328 | 0.006365871 | 0.3 | 0.58 | 0.82 | 0.95 | 0.99 | 1 |

.

R², explained variation by SNPs. OR, odds ratio.

**Supplementary Table 10.** Association of CVDs with age at first sexual intercourse and lifetime number of sexual partners using Mendelian randomisation

| Exposures | Outcomes | No. of  SNPs | IVW | | Weighted median | | Simple median | | MR-Egger | | MR-PRESSO^†^  (outlier-corrected) | |
| --- | --- | --- | --- | --- | --- | --- | --- | --- | --- | --- | --- | --- |
|  |  |  | OR  (95% CI) | p-value | OR  (95% CI) | p-value | OR  (95% CI) | p-value | OR  (95% CI) | p-value | OR  (95% CI) | p-value |
| CAD | Age at first sexual intercourse | 38 | 0.999(0.986-1.011) | 0.825 | 0.997(0.984-1.01) | 0.657 | 0.994(0.98-1.009) | 0.449 | 1.008(0.98-1.038) | 0.569 | 0.993(0.984-1.003) | 0.185 |
| MI |  | 23 | 0.998(0.984-1.012) | 0.768 | 0.997(0.982-1.012) | 0.715 | 0.995(0.978-1.013) | 0.592 | 1.022(0.991-1.054) | 0.173 | 0.993(0.983-1.003) | 0.196 |
| AF |  | 99 | 0.996(0.985-1.008) | 0.547 | 0.998(0.986-1.011) | 0.811 | 1.002(0.99-1.014) | 0.729 | 1.003(0.981-1.026) | 0.784 | 0.998(0.989-1.008) | 0.750 |
| HF |  | 9 | 1.032(1.005-1.06) | 0.022 | 1.028(0.993-1.065) | 0.114 | 1.023(0.987-1.061) | 0.210 | 1.012(0.936-1.094) | 0.767 | NA | NA |
| IS |  | 8 | 0.983(0.96-1.006) | 0.148 | 0.984(0.952-1.016) | 0.325 | 0.996(0.965-1.028) | 0.813 | 0.851(0.693-1.045) | 0.124 | NA | NA |
| CAD | Lifetime number of sexual partners | 39 | 1.001(0.988-1.015) | 0.840 | 1.004(0.99-1.018) | 0.585 | 1.006(0.992-1.02) | 0.406 | 0.982(0.952-1.012) | 0.240 | 1.004(0.992-1.016) | 0.504 |
| MI |  | 25 | 1.004(0.991-1.017) | 0.525 | 1.006(0.992-1.02) | 0.412 | 1.01(0.995-1.024) | 0.191 | 0.982(0.953-1.011) | 0.223 | 1.006(0.996-1.016) | 0.245 |
| AF |  | 111 | 0.999(0.991-1.006) | 0.713 | 0.999(0.988-1.009) | 0.787 | 0.997(0.988-1.006) | 0.548 | 0.998(0.984-1.012) | 0.742 | 1(0.993-1.006) | 0.925 |
| HF |  | 9 | 0.969(0.92-1.021) | 0.233 | 0.966(0.932-1.001) | 0.058 | 0.97(0.932-1.008) | 0.122 | 1.038(0.893-1.207) | 0.627 | 0.977(0.946-1.009) | 0.204 |
| IS |  | 7 | 1.007(0.986-1.029) | 0.530 | 1.007(0.979-1.035) | 0.633 | 1(0.971-1.03) | 0.994 | 1.066(0.909-1.25) | 0.433 | NA | NA |

CAD, coronary artery disease. MI, myocardial infarction. AF, atrial fibrillation. HF, heart failure. IS, ischemic stroke. OR, odds ratio. CI,confidence intervals. IVW, inverse-variance-weighted method. MR-PRESSO, MR pleiotropy residual sum and outlier method. SNPs, single-nucleotide polymorphisms.

†Details of outliers were displayed in Supplementary Table 14.

# **Supplementary** Table 11. MR-Egger pleiotropy test for all SNPS.

| **Outcomes** | **SNPs** | **MR-Egger** | | **MR-Egger intercept** | |  |
| --- | --- | --- | --- | --- | --- | --- |
|  |  | **OR(95%CI)** | **P-valve** | **OR(95%CI)** | **P-valve** | **I^2^_GX_** |
| Age at first sexual intercoursee | | | | | | |
| Coronary artery disease | 155 | 1.011(0.537-1.904) | 0.972013297 | 0.994(0.984-1.004) | 0.249279848 | 0.977826655 |
| Myocardial infarction | 155 | 0.996(0.517-1.919) | 0.98994196 | 0.995(0.985-1.005) | 0.334168165 | 0.977827676 |
| Atrial fibrillation | 156 | 1.311(0.833-2.065) | 0.241956638 | 0.992(0.985-0.999) | 0.027737088 | 0.97779627 |
| Heart failure | 158 | 0.632(0.382-1.046) | 0.074041909 | 1.001(0.993-1.009) | 0.746177729 | 0.977797187 |
| Ischemic stroke | 158 | 1.059(0.633-1.774) | 0.82603483 | 0.994(0.986-1.002) | 0.155913578 | 0.977791599 |
| Lifetime number of sexual partners | | | | | | |
| Coronary artery disease | 59 | 1.575(0.433-5.725) | 0.490253681 | 0.994(0.977-1.011) | 0.46644945 | 0.97344357 |
| Myocardial infarction | 58 | 1.761(0.46-6.737) | 0.40830339 | 0.994(0.977-1.012) | 0.518949609 | 0.972994724 |
| Atrial fibrillation | 60 | 1.393(0.508-3.821) | 0.51919991 | 0.995(0.982-1.009) | 0.499786373 | 0.973432013 |
| Heart failure | 54 | 1.936(0.668-5.609) | 0.223305924 | 0.989(0.975-1.003) | 0.128849629 | 0.972824869 |
| Ischemic stroke | 61 | 1.01(0.317-3.215) | 0.986183026 | 1.001(0.986-1.017) | 0.851021905 | 0.97383761 |

SNPs, Single-nucleotide polymorphiss. OR, odds ratio.CI,confidence intervals.

# **Supplementary** Table 12. MR-Egger pleiotropy test after removing SNPs associated with confounders or CVDs.

| **Outcomes** | **SNPs** | **MR-Egger** | | **MR-Egger intercept** | |  |
| --- | --- | --- | --- | --- | --- | --- |
|  |  | **OR(95%CI)** | **P-valve** | **OR(95%CI)** | **P-valve** | **I^2^_GX_** |
| Age at first sexual intercourse | | | | | | |
| Coronary artery disease | 107 | 1.485(0.777-2.838) | 0.231890948 | 0.989(0.98-0.999) | 0.032734501 | 0.976184555 |
| Myocardial infarction | 107 | 1.268(0.639-2.515) | 0.496453554 | 0.992(0.982-1.003) | 0.138400243 | 0.976188153 |
| Atrial fibrillation | 107 | 1.525(0.881-2.64) | 0.13138735 | 0.991(0.983-0.999) | 0.035041654 | 0.976223009 |
| Heart failure | 108 | 0.733(0.42-1.279) | 0.274089183 | 1(0.992-1.009) | 0.955705125 | 0.976281692 |
| Ischemic stroke | 108 | 0.957(0.513-1.784) | 0.88913874 | 0.996(0.987-1.005) | 0.398361648 | 0.976258385 |
| Lifetime number of sexual partners | | | | | | |
| Coronary artery disease | 43 | 2.062(0.713-5.961) | 0.181580495 | 0.992(0.978-1.006) | 0.266201658 | 0.973134557 |
| Myocardial infarction | 43 | 2.208(0.693-7.035) | 0.180330624 | 0.994(0.979-1.01) | 0.46812525 | 0.973153981 |
| Atrial fibrillation | 43 | 1.143(0.456-2.866) | 0.776082142 | 0.999(0.987-1.011) | 0.860682406 | 0.973183462 |
| Heart failure | 39 | 1.532(0.575-4.081) | 0.393482117 | 0.994(0.981-1.007) | 0.343131459 | 0.973217218 |
| Ischemic stroke | 43 | 0.738(0.214-2.541) | 0.630407382 | 1.006(0.99-1.023) | 0.466588949 | 0.973235733 |

SNPs, Single-nucleotide polymorphiss.OR, odds ratio.CI,confidence intervals.

**Supplementary Table 13.** SNPs excluded from the outlier corrected MR-PRESSO analyses between age at first sexual intercourse and lifetime number of sexual partners with CVD.

| **Exposures** | **Outcome** | **SNP selection*** | **OutlierSNP** | **RSSobs** | **P-value** |
| --- | --- | --- | --- | --- | --- |
| Age at first sexual intercourse | Coronary artery disease | All | rs12204714 | 0.001706806 | <0.155 |
|  |  |  | rs4728298 | 0.001934735 | <0.155 |
|  |  | Removed | NA | NA | NA |
|  | Myocardial infarction | All | rs12244388 | 0.001175585 | <0.155 |
|  |  |  | rs4809230 | 0.00236331 | <0.155 |
|  |  | Removed | NA | NA | NA |
|  | Atrial fibrillation | All | rs1320330 | 0.001190986 | <0.156 |
|  |  |  | rs1925686 | 0.001579047 | <0.156 |
|  |  | Removed | rs1925686 | 0.001662832 | <0.107 |
|  | Heart failure | All | rs34804222 | 0.000575527 | <0.158 |
|  |  | Removed | NA | NA | NA |
|  | Ischemic stroke | All | NA | NA | NA |
|  |  | Removed | NA | NA | NA |
| Lifetime number of sexual partners | Coronary artery disease | All | rs12244388 | 0.001368418 | <0.059 |
|  |  |  | rs4702 | 0.001443779 | <0.059 |
|  |  | Removed | NA | NA | NA |
|  | Myocardial infarction | All | rs4702 | 0.002045353 | <0.058 |
|  |  | Removed | NA | NA | NA |
|  | Atrial fibrillation | All | rs11642841 | 0.000890194 | <0.06 |
|  |  |  | rs17690703 | 0.001291999 | <0.06 |
|  |  |  | rs4869737 | 0.000959079 | <0.06 |
|  |  | Removed | rs3740484 | 0.001016388 | <0.043 |
|  | Heart failure | All | rs11642841 | 0.001373612 | <0.054 |
|  |  | Removed | NA | NA | NA |
|  | Ischemic stroke | All | NA | NA | NA |
|  |  | Removed | NA | NA | NA |

* ‘All’ represents analyses with all selected SNPs, ‘Removed’ represents analyses after removing SNPs associated with relevant confounders or CVDs.

**Supplementary Table 14.** SNPs excluded from the outlier corrected MR-PRESSO analyses between CVD with age at first sexual intercourse and lifetime number of sexual partners.

| Exposure | Outcome | OutlierSNP | RSSobs | P-value |
| --- | --- | --- | --- | --- |
| CAD | Age at first sexual intercourse | rs663129 | 0.000130175 | <0.038 |
| MI | Age at first sexual intercourse | rs72934535 | 0.000240474 | <0.023 |
| AF | Age at first sexual intercourse | rs12245149 | 8.69E-05 | <0.099 |
|  |  | rs2031522 | 0.000141523 | <0.099 |
|  |  | rs2759301 | 7.04E-05 | <0.099 |
|  |  | rs56326533 | 0.00010962 | <0.099 |
|  |  | rs6994744 | 5.59E-05 | <0.099 |
| HF | Age at first sexual intercourse | NA | NA | NA |
| IS | Age at first sexual intercourse | NA | NA | NA |
| CAD | Lifetime number of sexual partners | rs11191416 | 0.000150389 | <0.039 |
|  |  | rs115654617 | 0.000121706 | <0.039 |
|  |  | rs2487928 | 4.23E-05 | <0.039 |
| MI | Lifetime number of sexual partners | rs72934535 | 0.000172009 | <0.025 |
| AF | Lifetime number of sexual partners | rs1563304 | 0.000158481 | <0.111 |
| HF | Lifetime number of sexual partners | rs56094641 | 8.80E-05 | <0.009 |
| IS | Lifetime number of sexual partners | NA | NA | NA |

CAD, coronary artery disease. MI, myocardial infarction. AF, atrial fibrillation. HF, heart failure. IS, ischemic stroke.

**Supplementary Figure 1.** The diagram of bi-directional MR analysis.


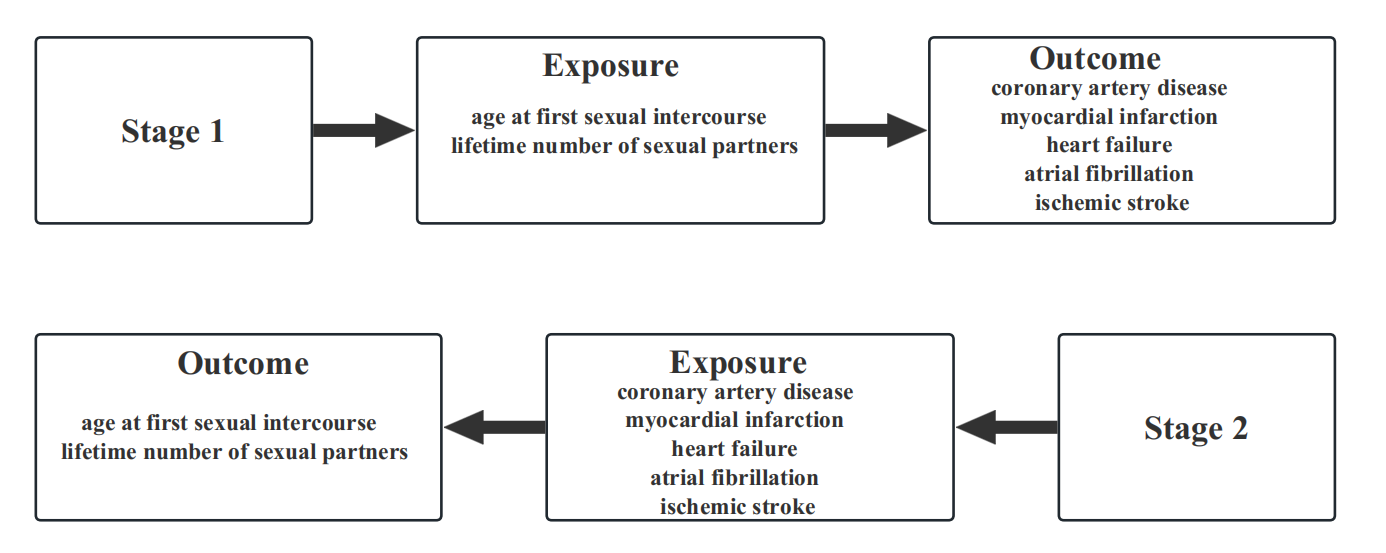


In stage 1 analysis, causal association of genetically predicted sexual factors (age at first sexual intercourse and lifetime number of sexual partners) on risk of cardiovascular disease was estimated. In stage 2 analysis, causal association of genetically predicted cardiovascular disease on risk of sexual factors was estimated.

**Supplementary Figure 2.** Scatter plots for the causal association of age at first sexual intercourse with outcomes.


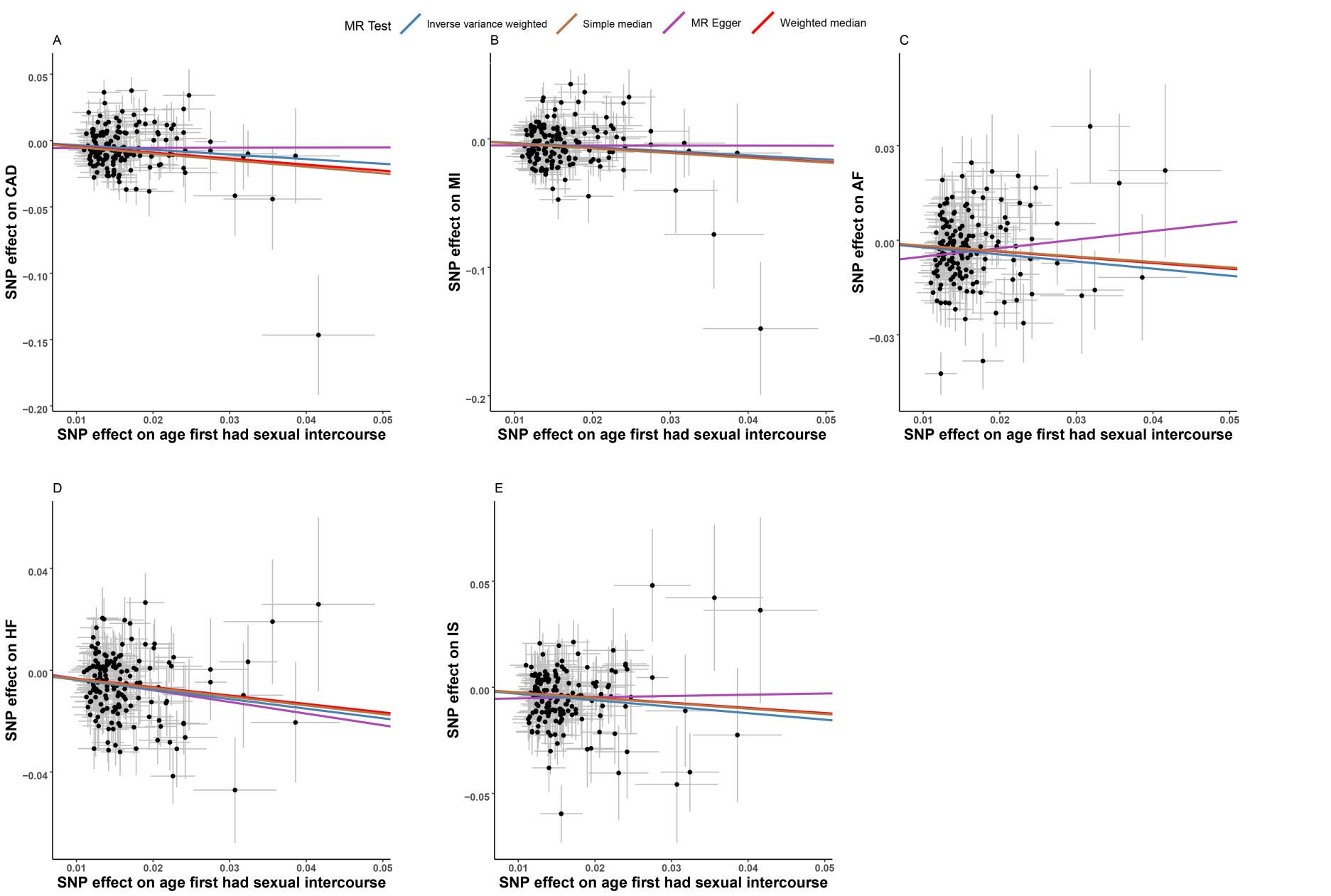
The slope of each line corresponding to estimated Mendelian Randomization (MR) effect per method. Circles indicate marginal genetic associations of each variant with age at first sexual intercourse and the risk of outcomes. Error bars indicate 95% CIs. CAD, coronary artery disease; MI, myocardial infarction; AF, atrial fibrillation; HF, heart failure; IS, ischemic stroke.

**Supplementary Figure 3.** Scatter plots for the causal association of lifetime number of sexual partners with outcomes.


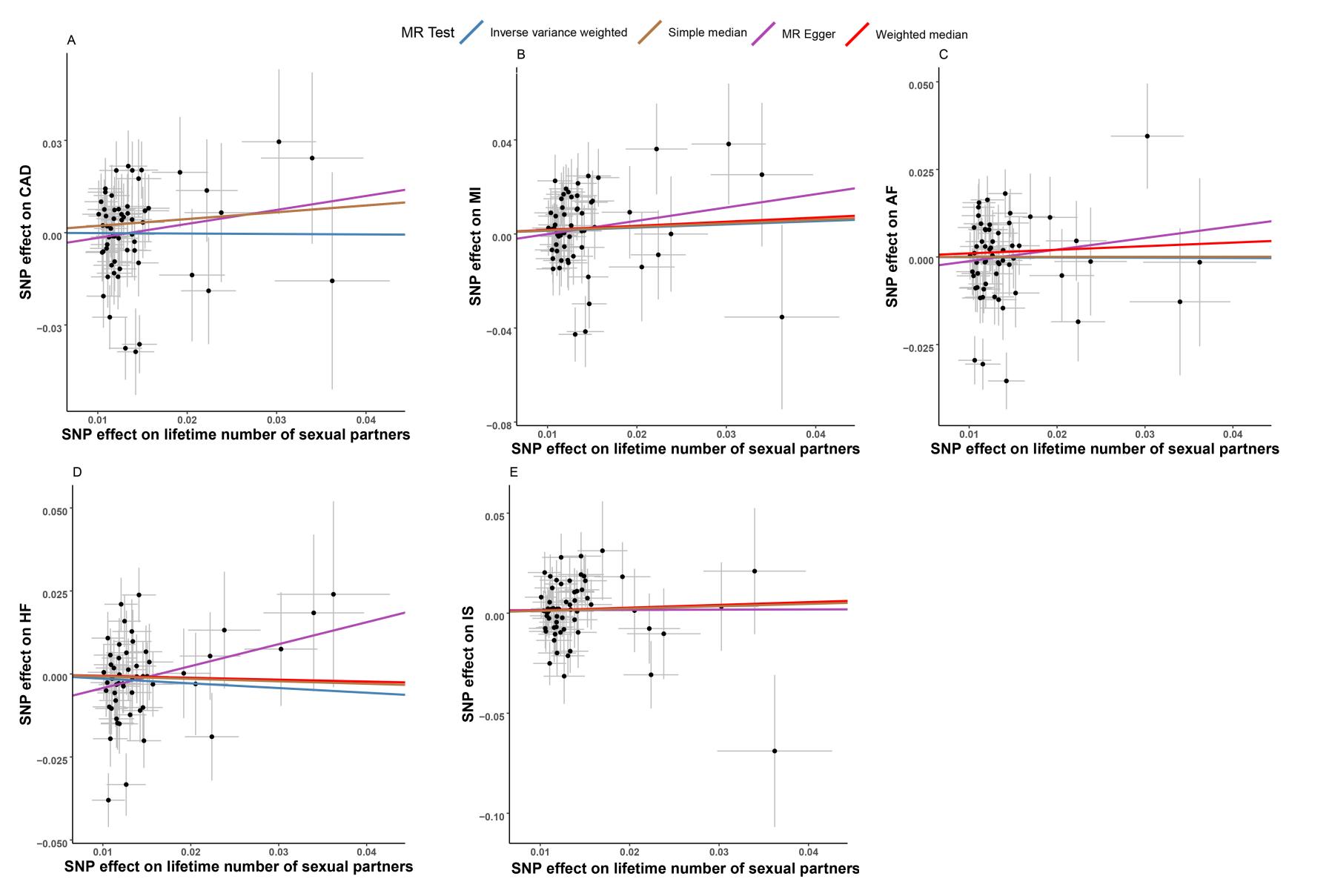


The slope of each line corresponding to estimated Mendelian Randomization (MR) effect per method. Circles indicate marginal genetic associations of each variant with lifetime number of sexual partners and the risk of outcomes. Error bars indicate 95% CIs. CAD, coronary artery disease; MI, myocardial infarction; AF, atrial fibrillation; HF, heart failure; IS, ischemic stroke.

# Supplementary Figure 4. Scatter plots for the causal association of age at first sexual intercourse with outcomes after removing the SNPs associated with confounders or CVDs.

#
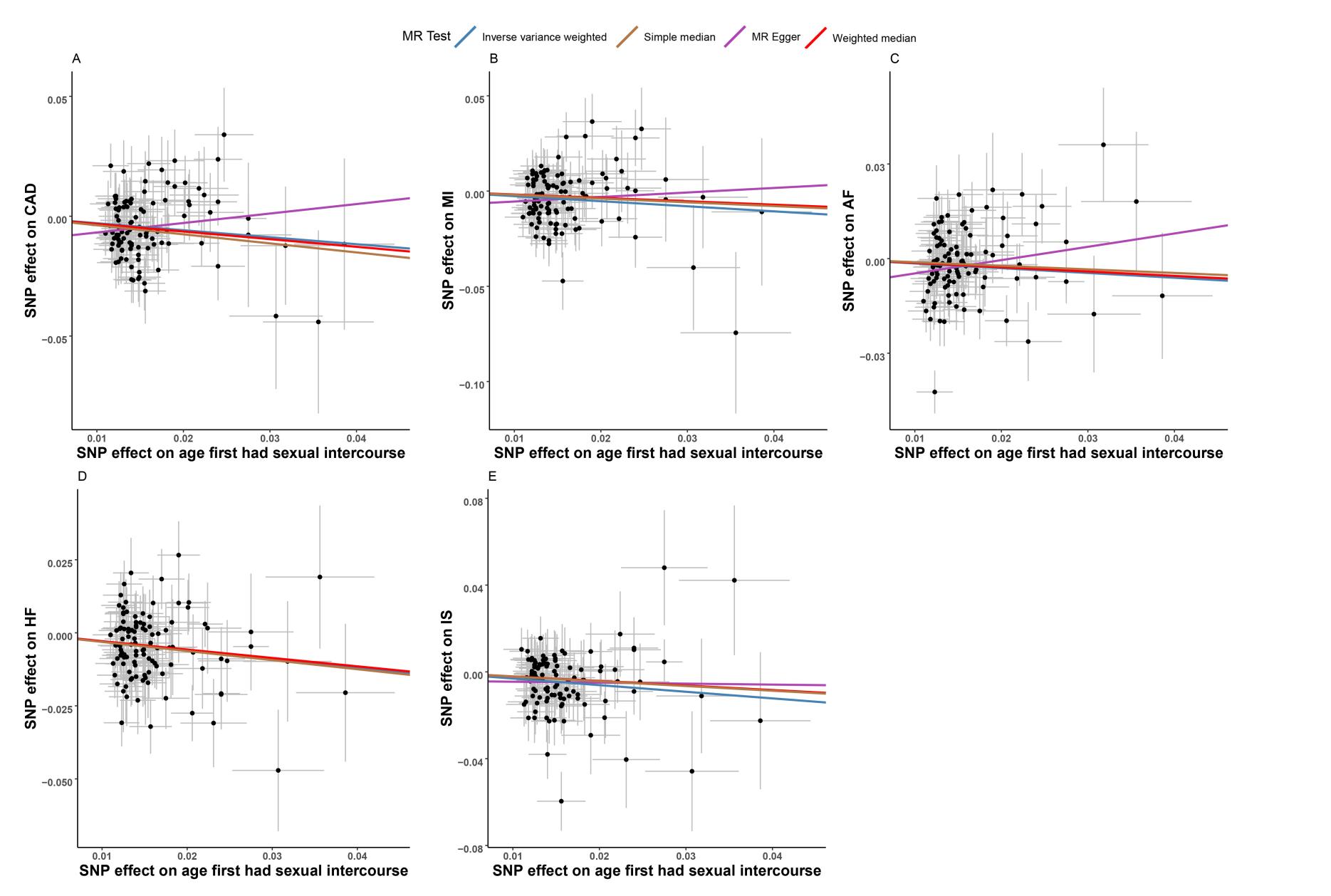


The slope of each line corresponding to estimated Mendelian Randomization (MR) effect per method. Circles indicate marginal genetic associations of each variant with age at first sexual intercourse and the risk of outcomes. Error bars indicate 95% CIs. CAD, coronary artery disease; MI, myocardial infarction; AF, atrial fibrillation; HF, heart failure; IS, ischemic stroke.

# Supplementary Figure 5. Scatter plots for the causal association of lifetime number of sexual partners with outcomes after removing the SNPs associated with confounders or CVDs.


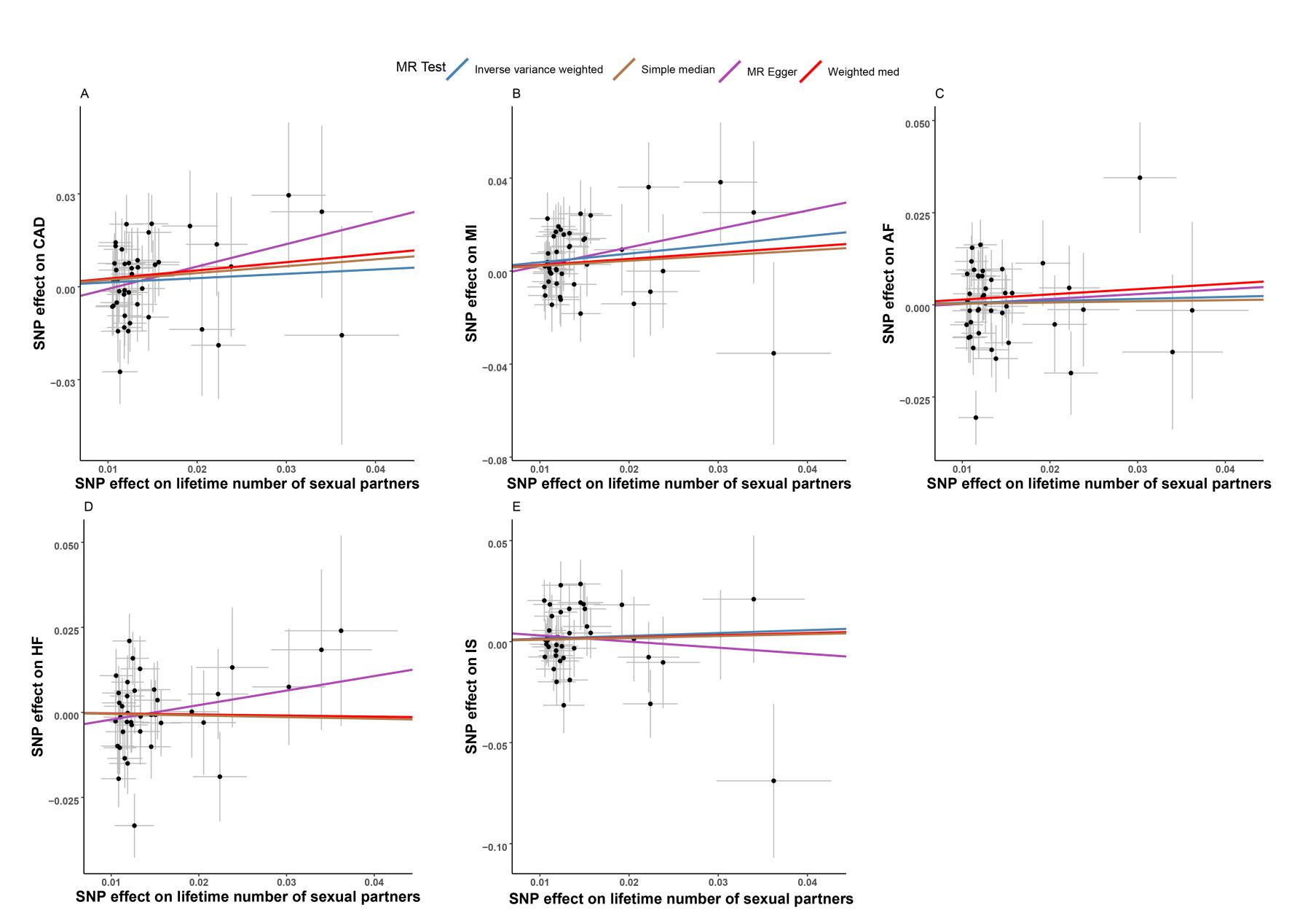


The slope of each line corresponding to estimated Mendelian Randomization (MR) effect per method. Circles indicate marginal genetic associations of each variant with lifetime number of sexual partners and the risk of outcomes. Error bars indicate 95% CIs. CAD, coronary artery disease; MI, myocardial infarction; AF, atrial fibrillation; HF, heart failure; IS, ischemic stroke.

**Supplementary Figure 6.** Causal association between age at first sexual intercourse with cardiovascular diseases after removing the SNPs associated with confounders or CVDs.


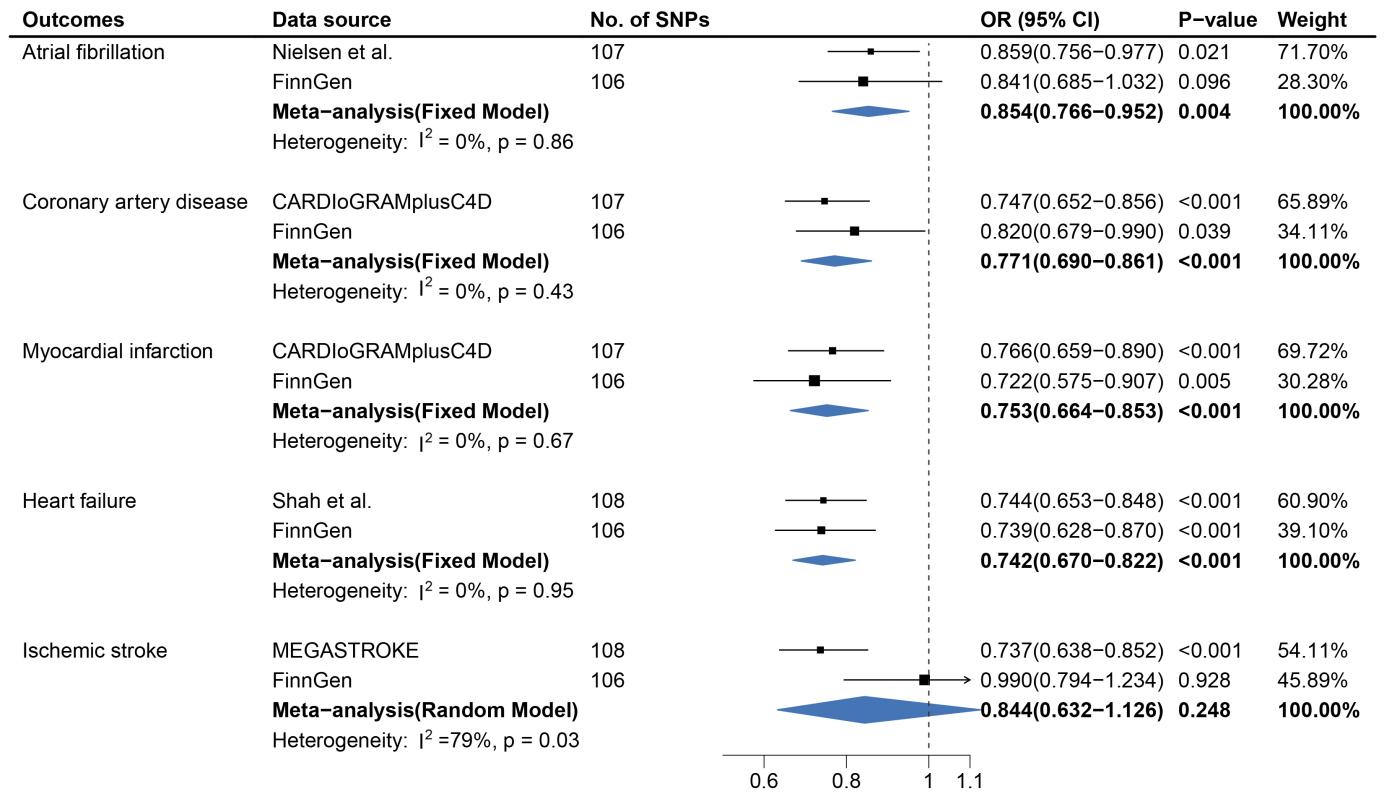


Estimated odds ratios of cardiovascular disease per 1-unit increase in age at first sexual intercourse were determined from the primary IVW analysis. These estimations were done for each outcome data source separately, and then combined across the two data sources using a meta-analysis.

**Supplementary Figure 7.** Causal association between lifetime number of sexual partners with cardiovascular diseases after removing the SNPs associated with confounders or CVDs.


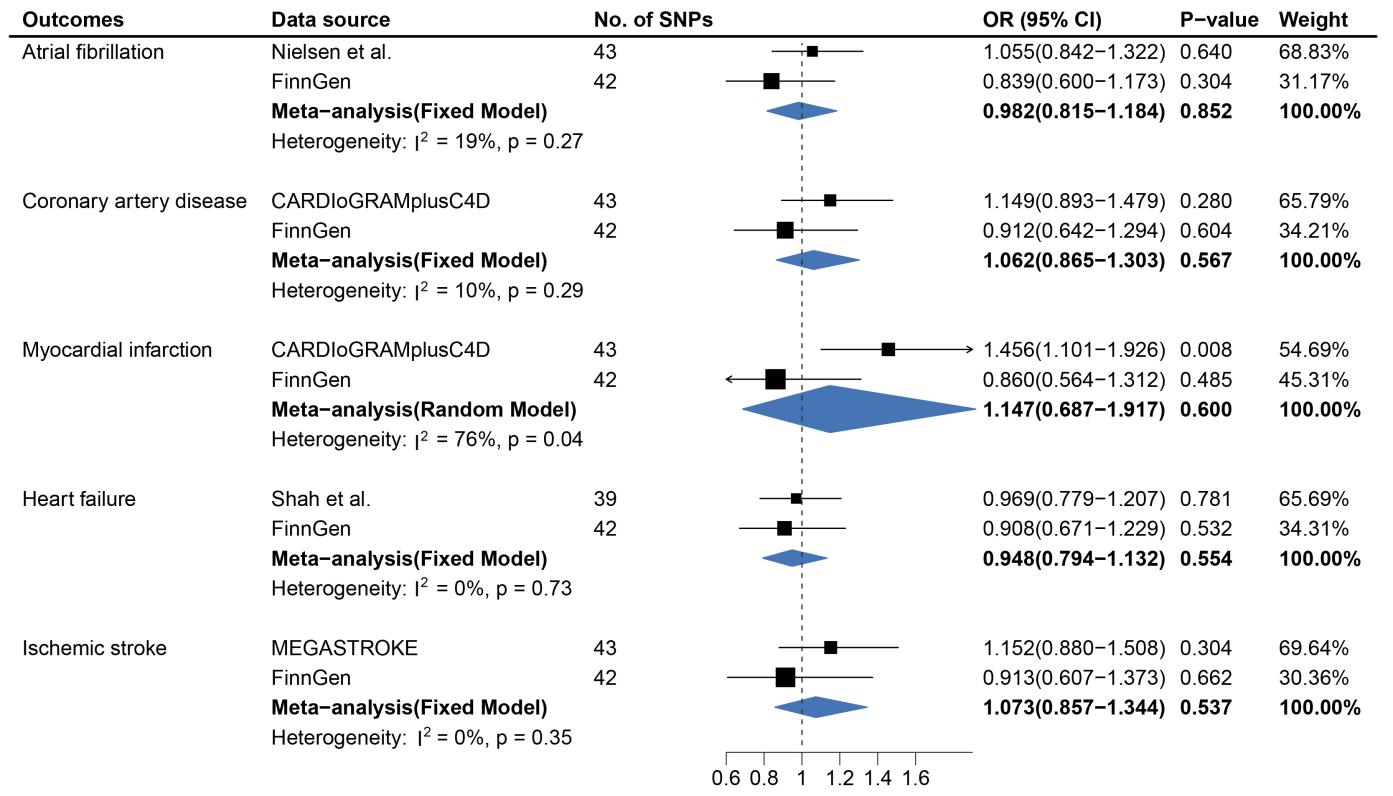


Estimated odds ratios of cardiovascular disease per 1-unit increase in lifetime number of sexual were determined from the primary IVW analysis. These estimations were done for each outcome data source separately, and then combined across the two data sources using a meta-analysis.

**Supplementary Figure 8.** Complementary sensitivity analyses of the association between sexual factors and cardiovascular diseases after removing the SNPs associated with confounders or CVDs.


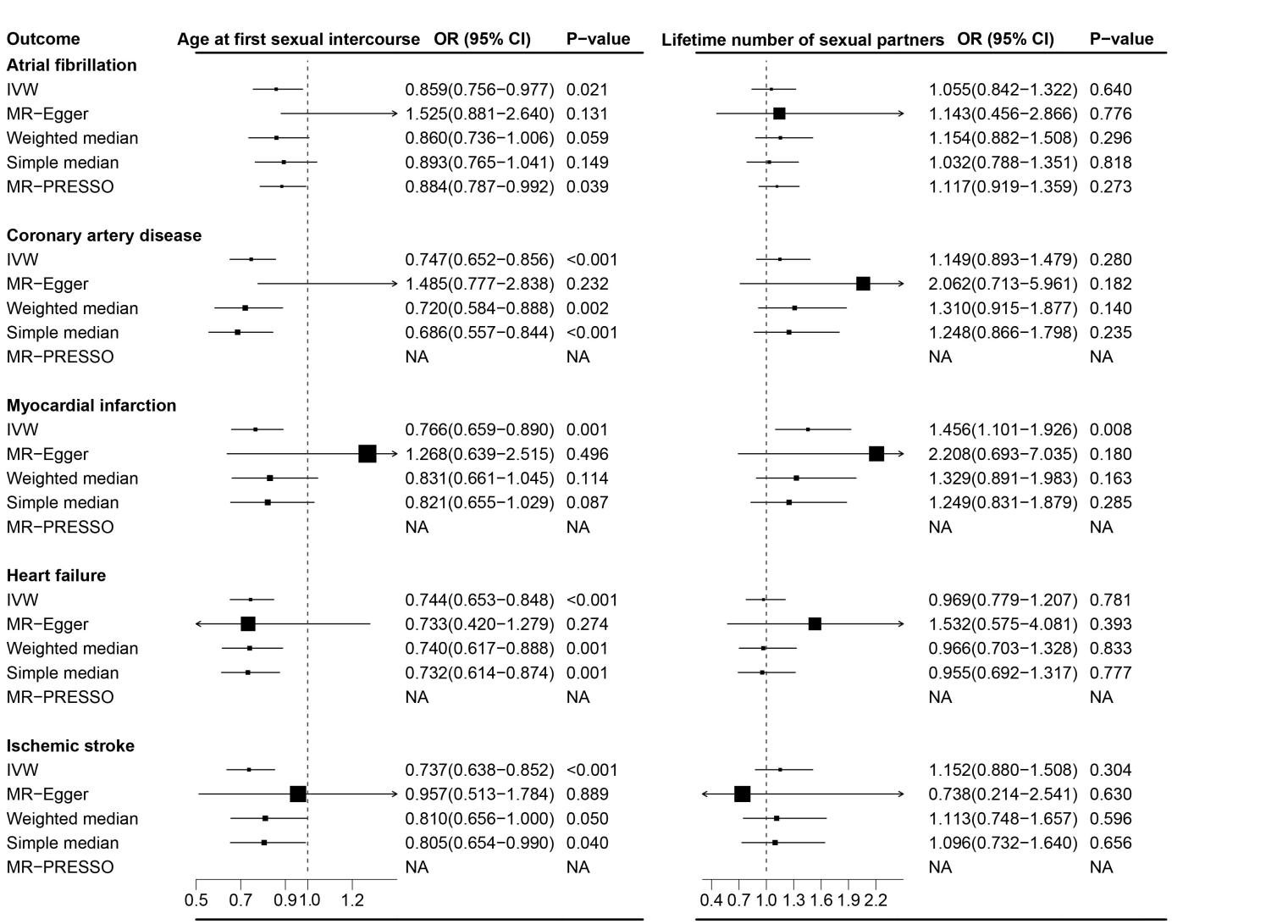


IVW, inverse-variance weighted; MR, Mendelian randomization; MR-PRESSO, MR Pleiotropy RESidual Sum and Outlier.

#
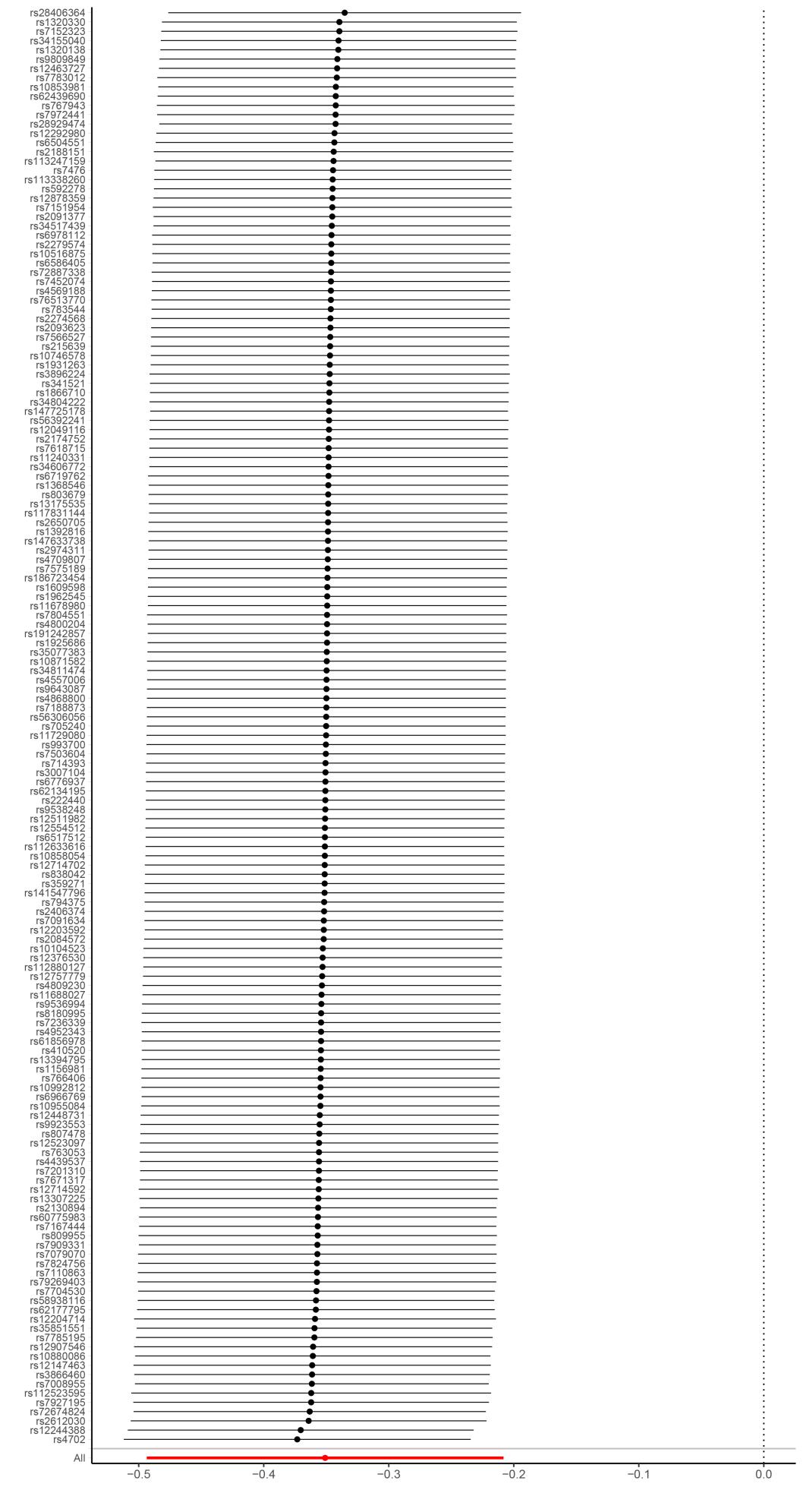
Supplementary Figure 9. MR Leave one out analyses for age at first sexual intercourse on coronary artery disease.

#
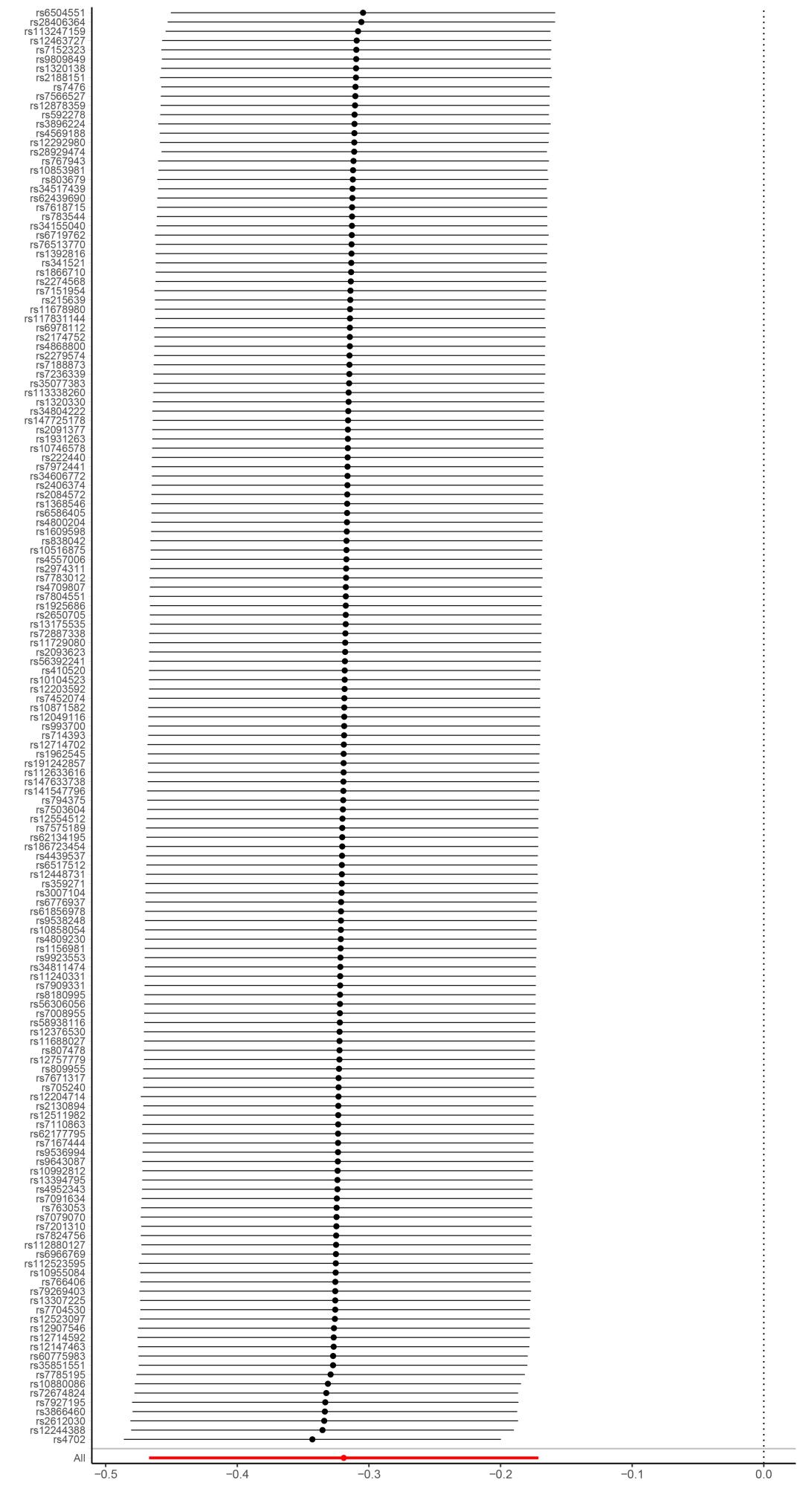
Supplementary Figure 10. MR Leave one out analyses for age at first sexual intercourse on myocardial infarction.

**Supplementary Figure 11.** MR Leave one out analyses for age at first sexual intercourse on atrial fibrillation.


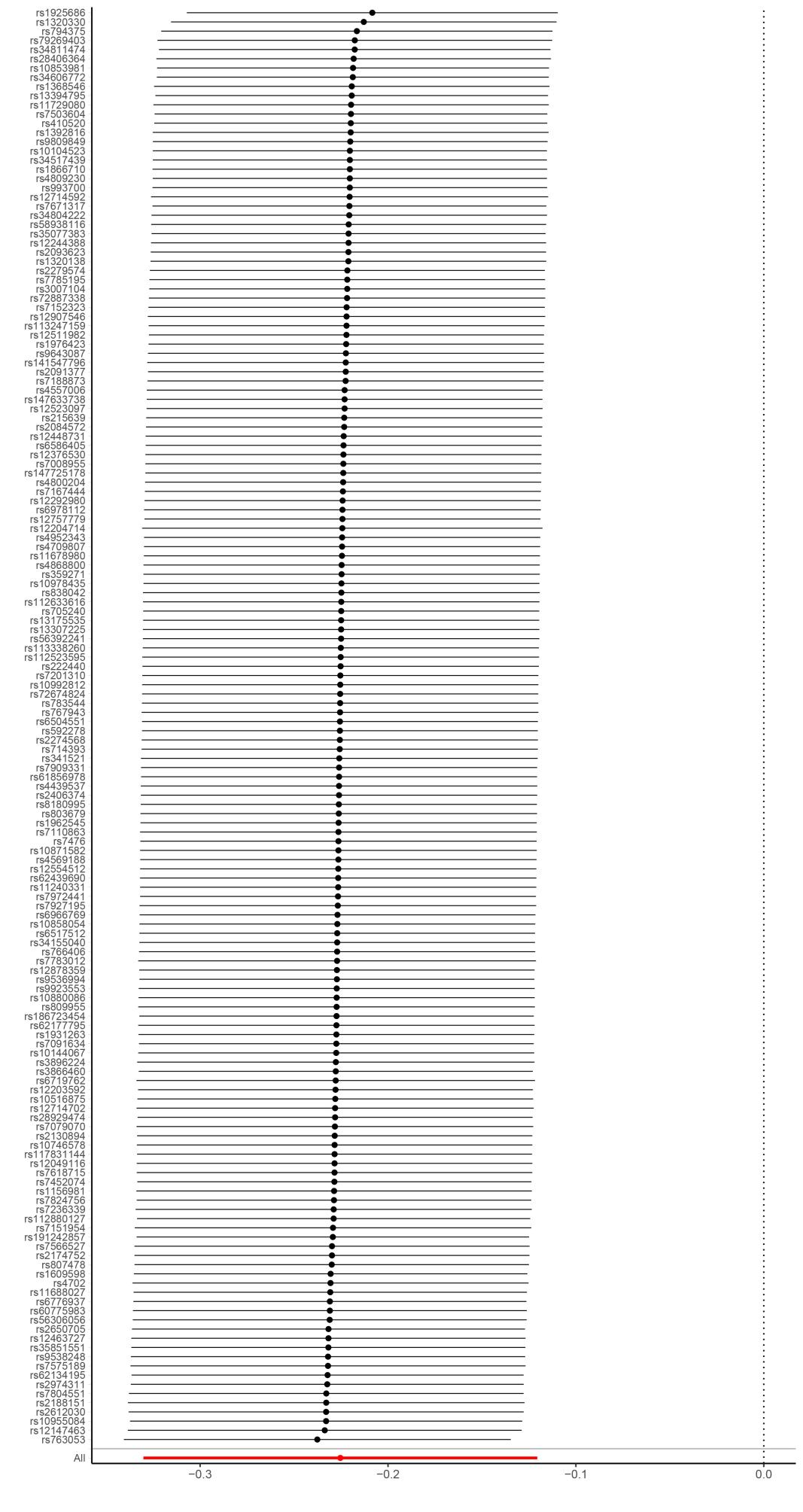


**Supplementary Figure 12.**
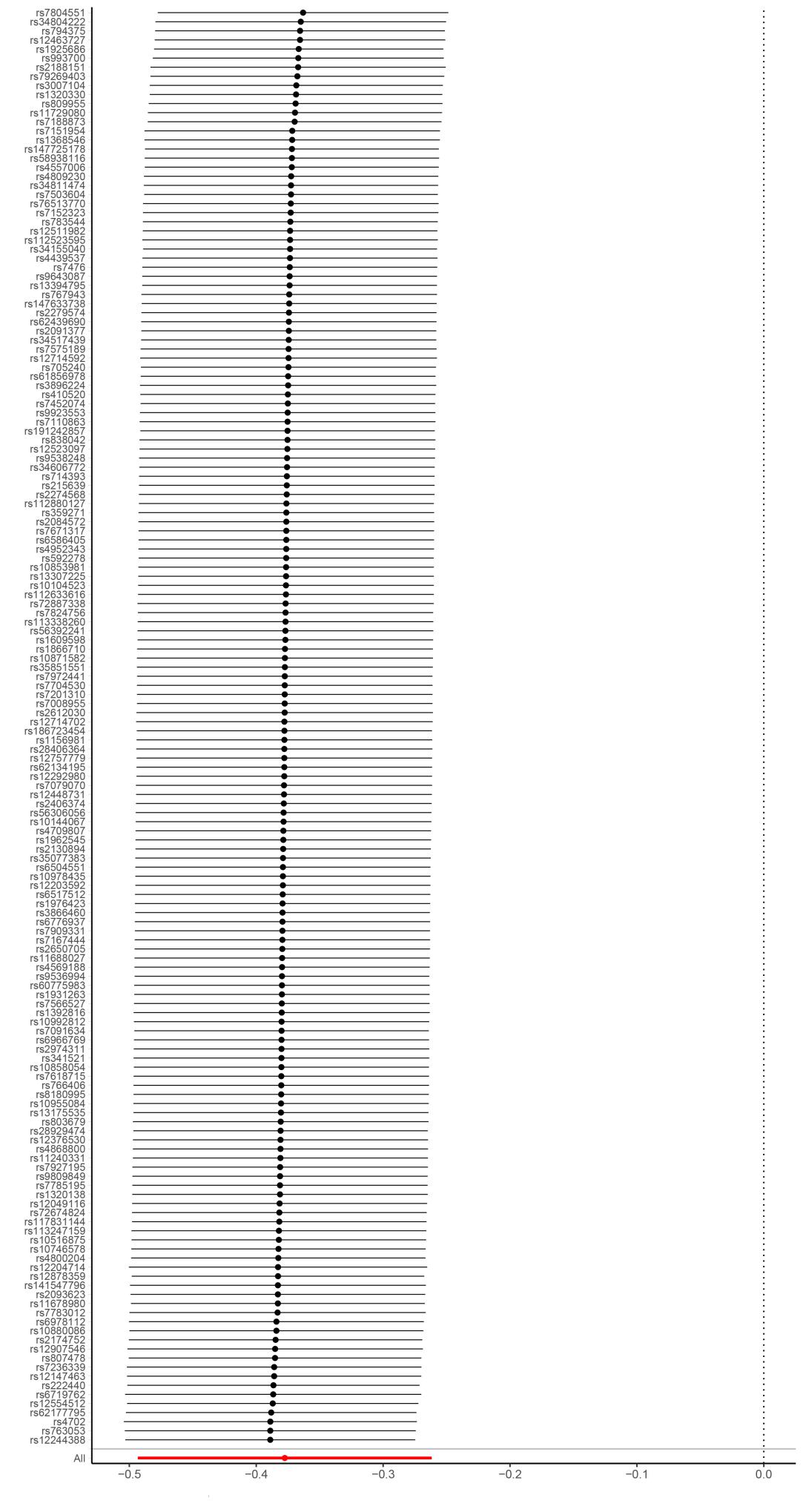
 MR Leave one out analyses for age at first sexual intercourse on heart failure.


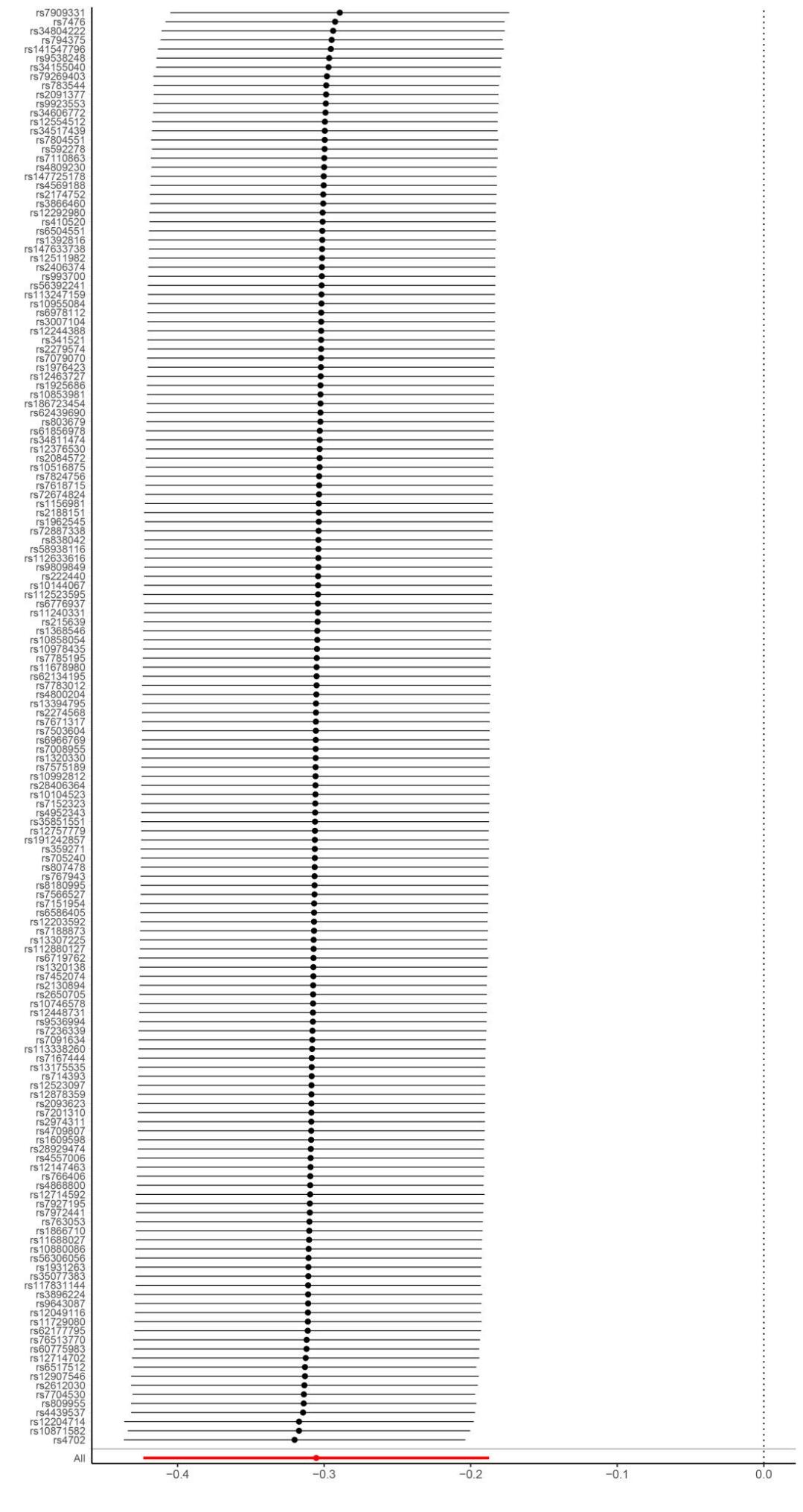
**Supplementary Figure 13.** MR Leave one out analyses for age at first sexual intercourse on ischemic stroke.

#
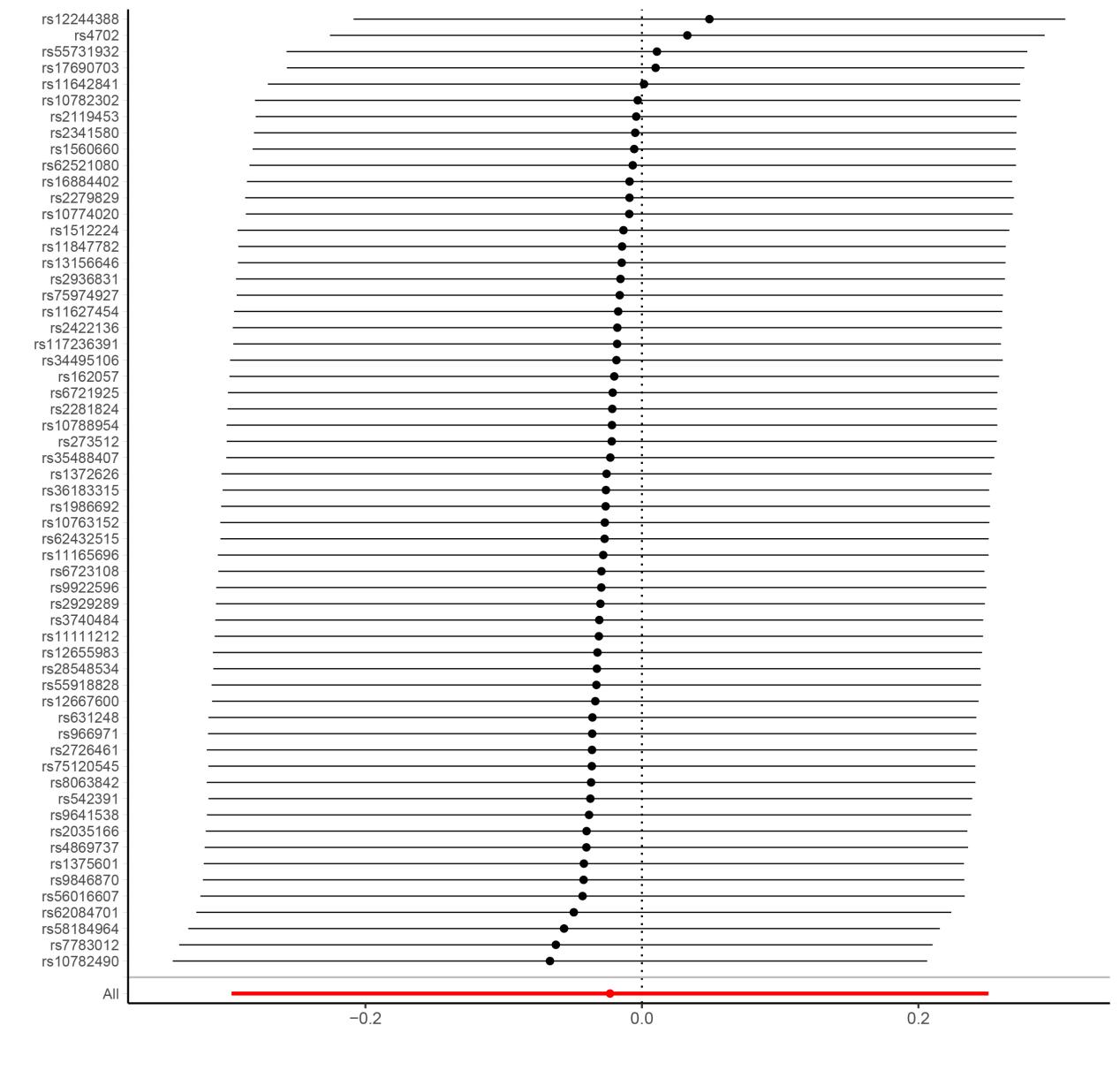
Supplementary Figure 14. MR Leave one out analyses for lifetime number of sexual partners on coronary artery disease.

# Supplementary Figure 15. MR Leave one out analyses for lifetime number of sexual partners on myocardial infarction.


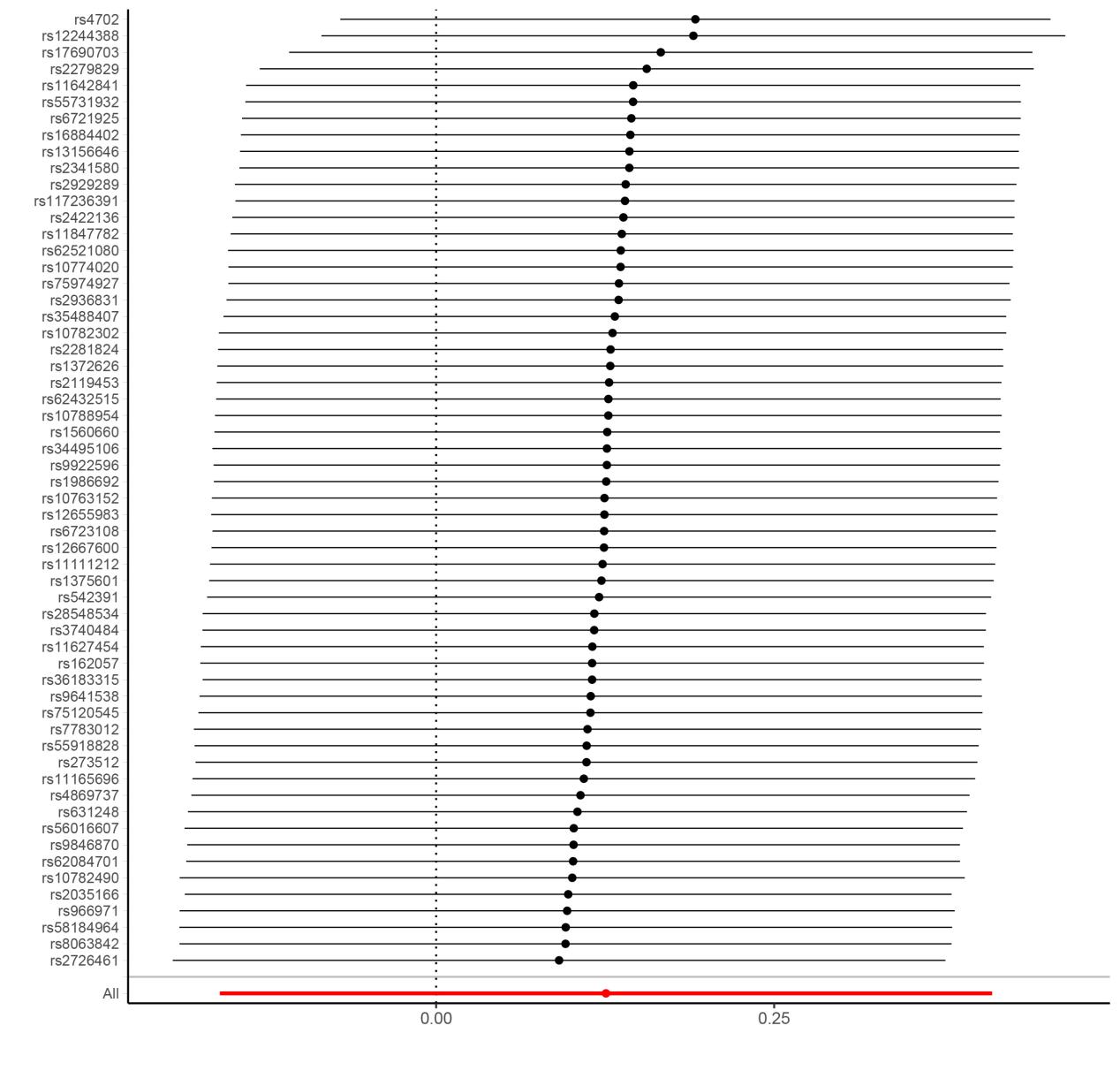


# Supplementary Figure 16. MR Leave one out analyses for lifetime number of sexual partners on atrial fibrillation.


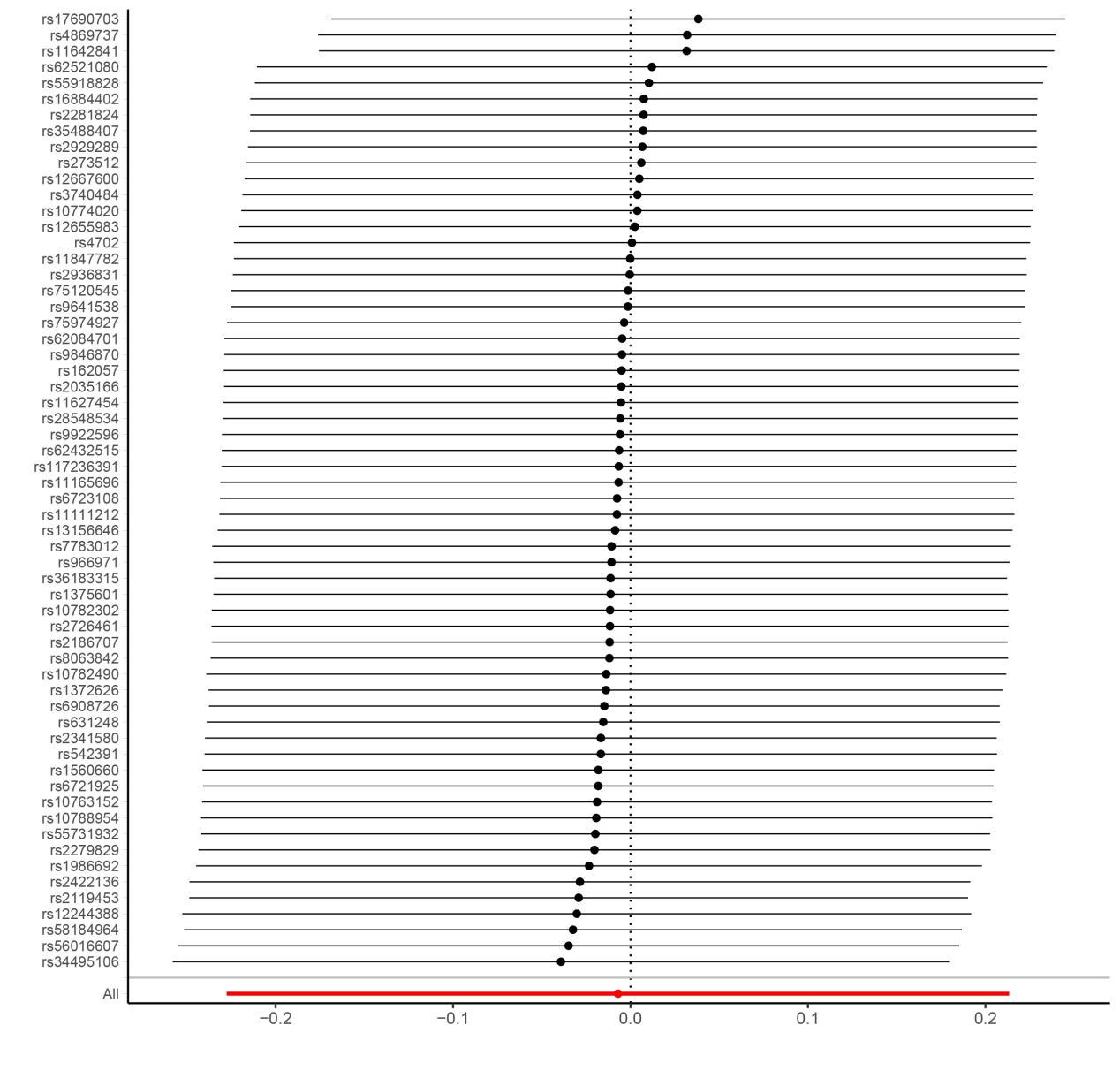


# Supplementary Figure 17. MR Leave one out analyses for lifetime number of sexual partners on heart failure.


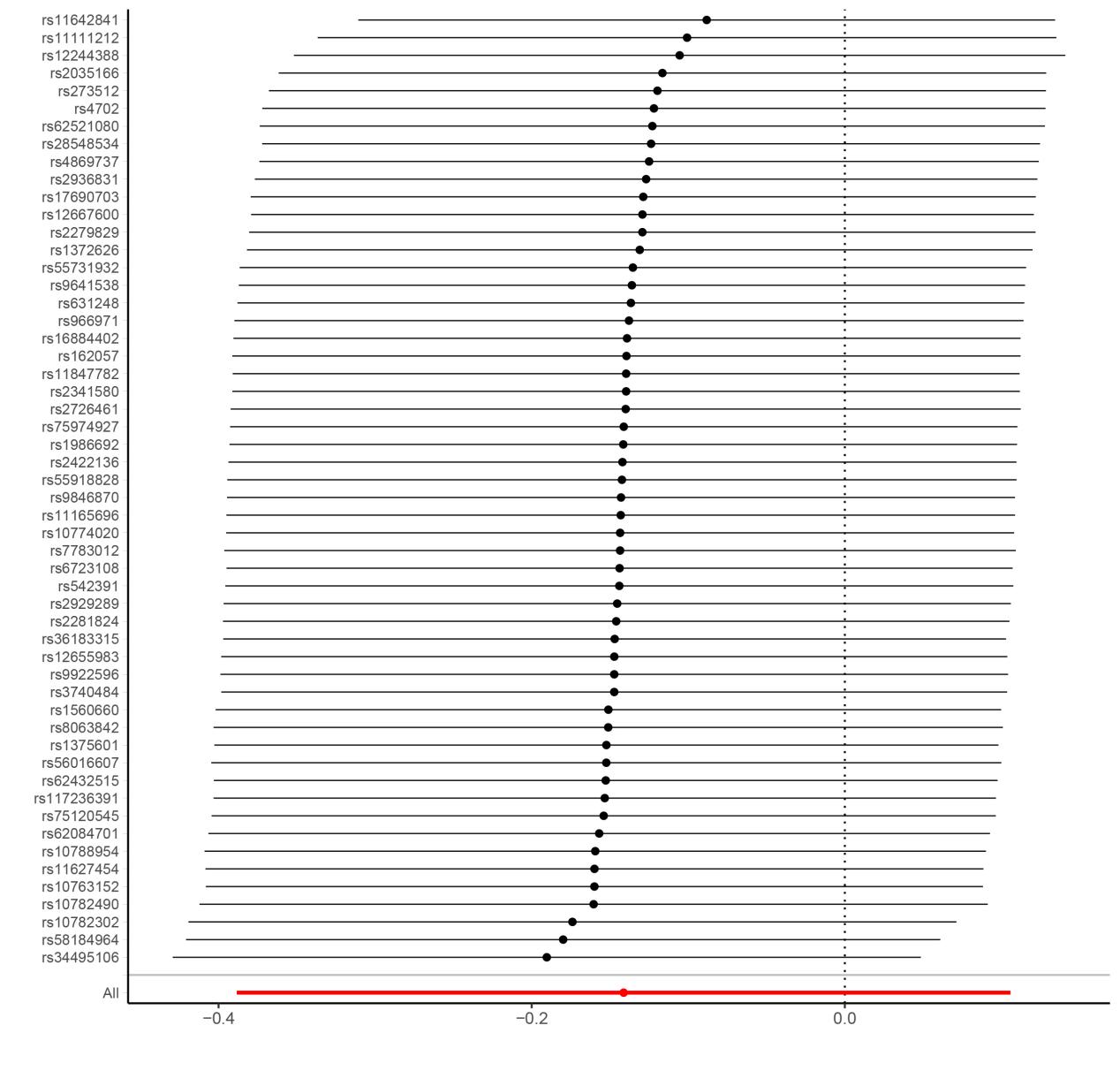


# Supplementary Figure 18. MR Leave one out analyses for lifetime number of sexual partners on ischemic stroke.


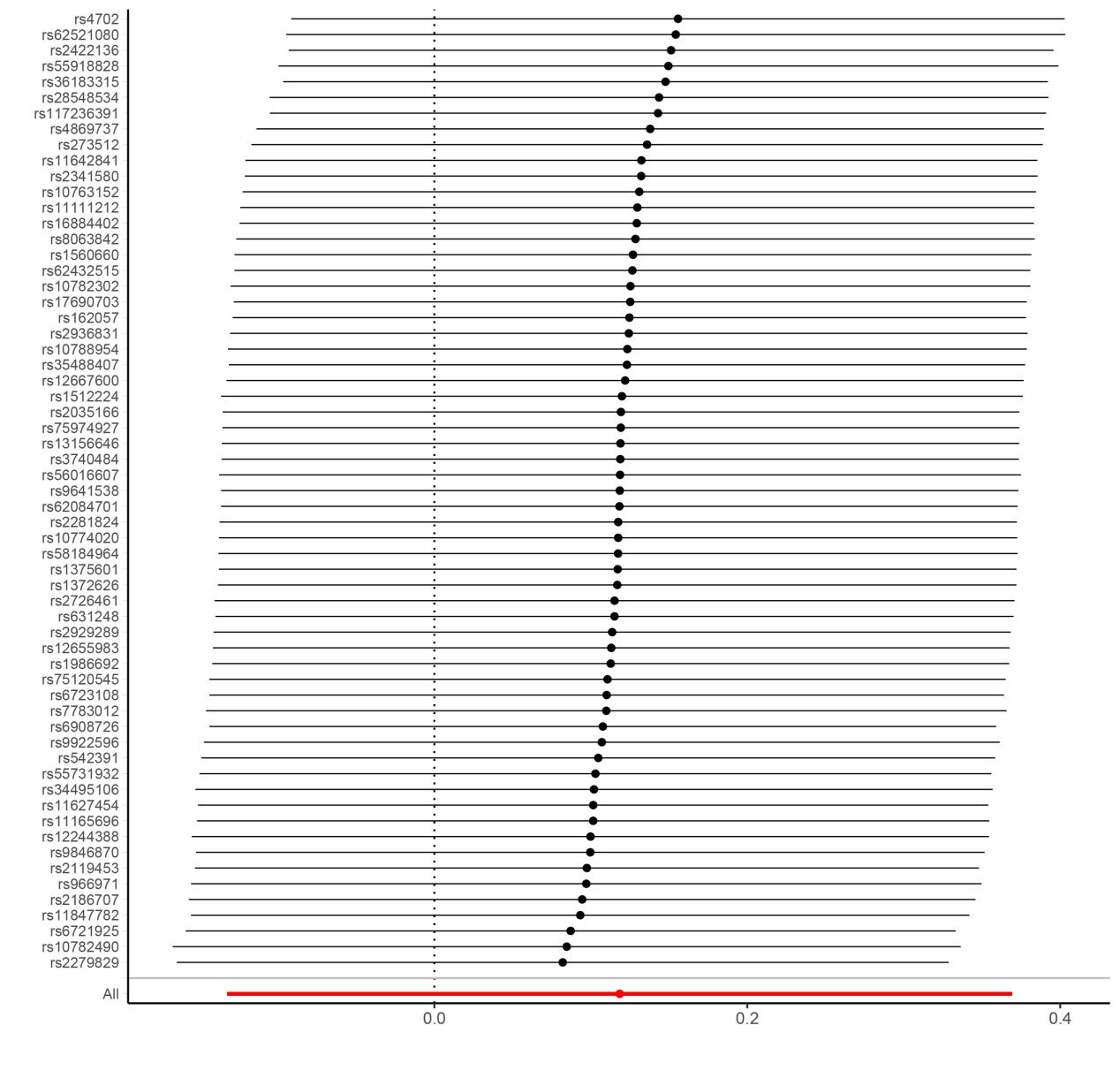


#
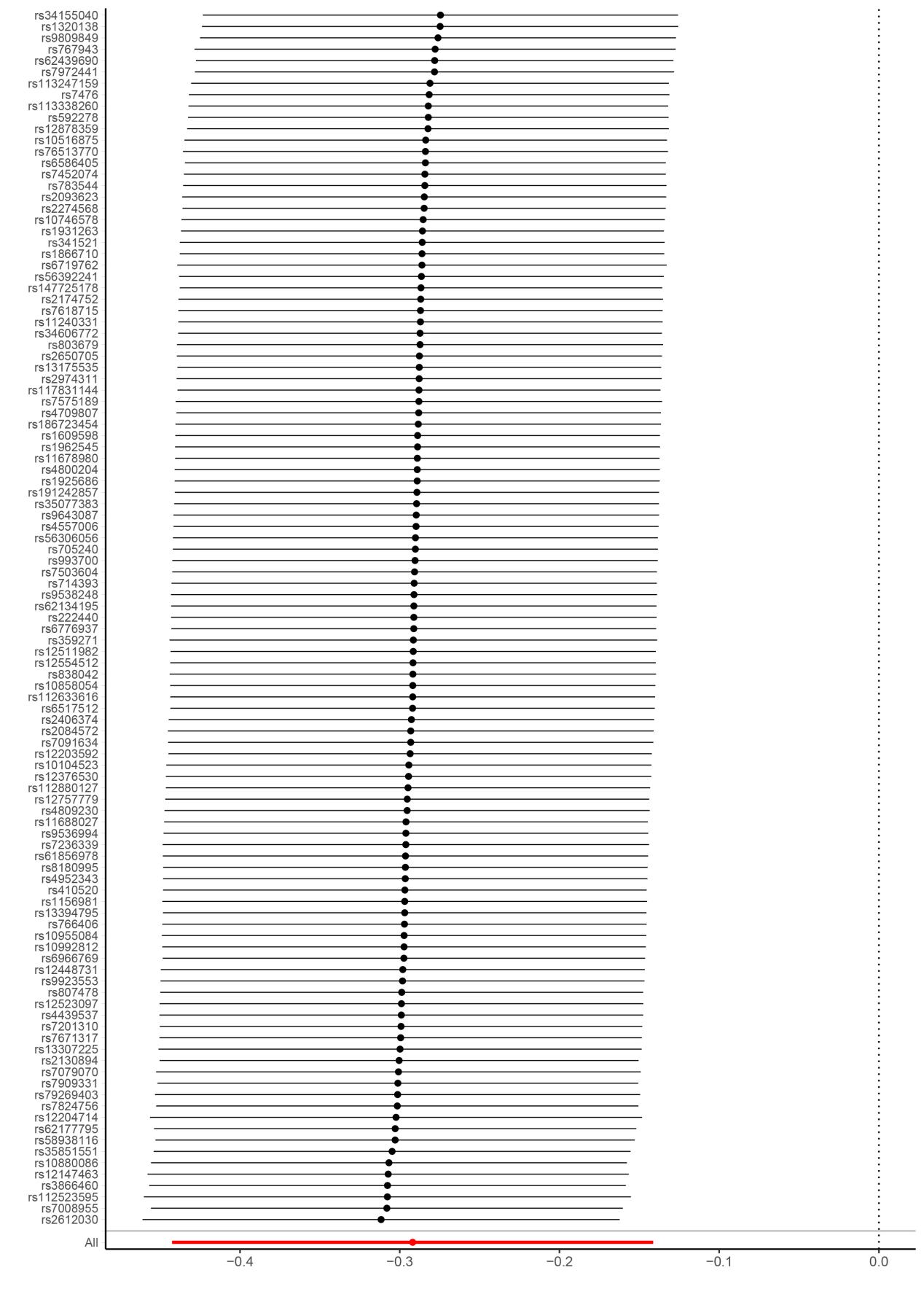
**Supplementary Figure 19.** MR Leave one out analyses for age at first sexual intercourse on coronary artery disease after removing SNPs associated with confounders or CVDs.

#
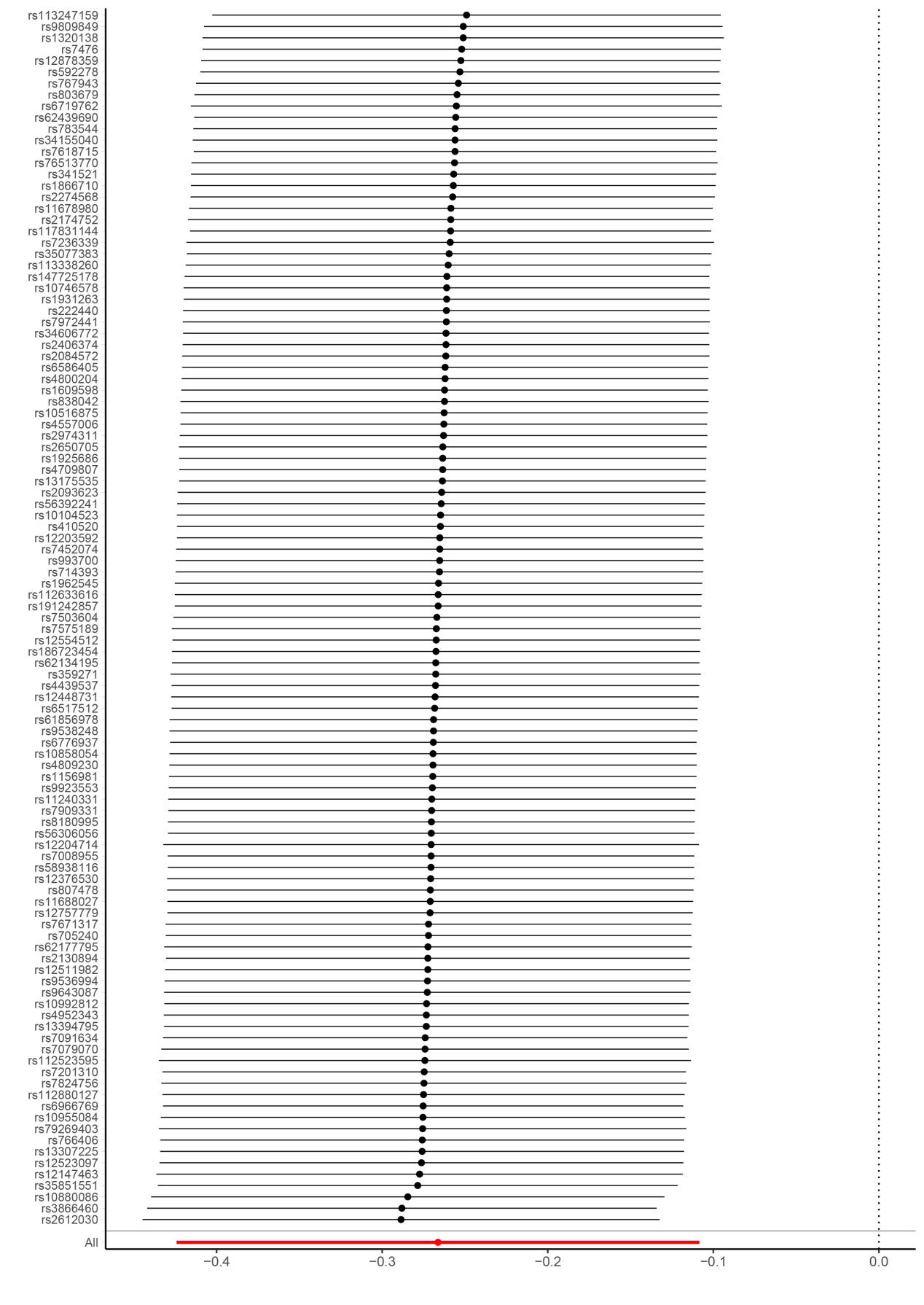
**Supplementary Figure 20.** MR Leave one out analyses for age at first sexual intercourse on myocardial infarction after removing SNPs associated with confounders or CVDs.

#
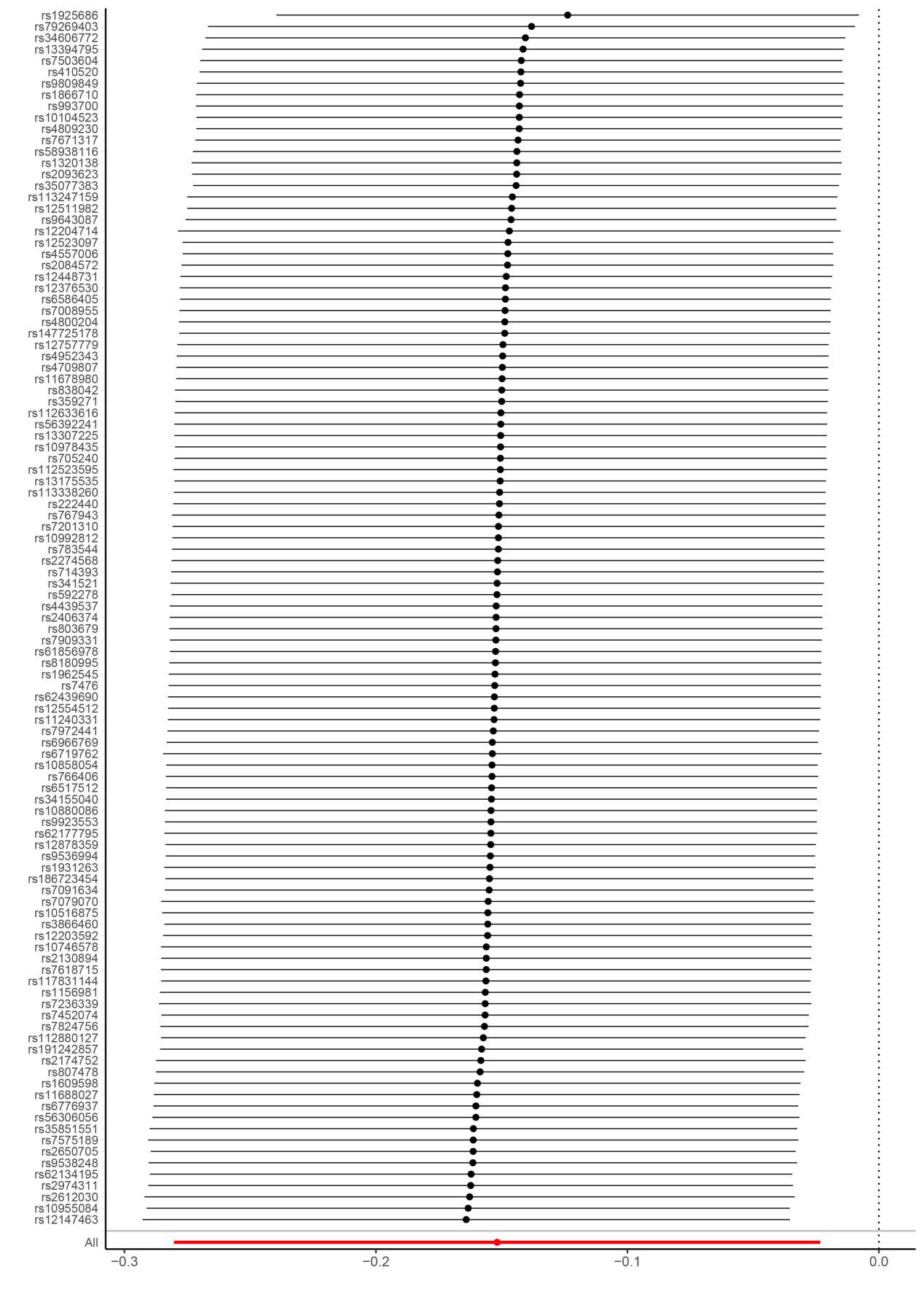
**Supplementary Figure 21.** MR Leave one out analyses for age at first sexual intercourse on atrial fibrillation after removing SNPs associated with confounders or CVDs.

# **Supplementary Figure 22.** MR Leave one out analyses for age at first sexual intercourse on heart failure after removing SNPs associated with confounders or CVDs.


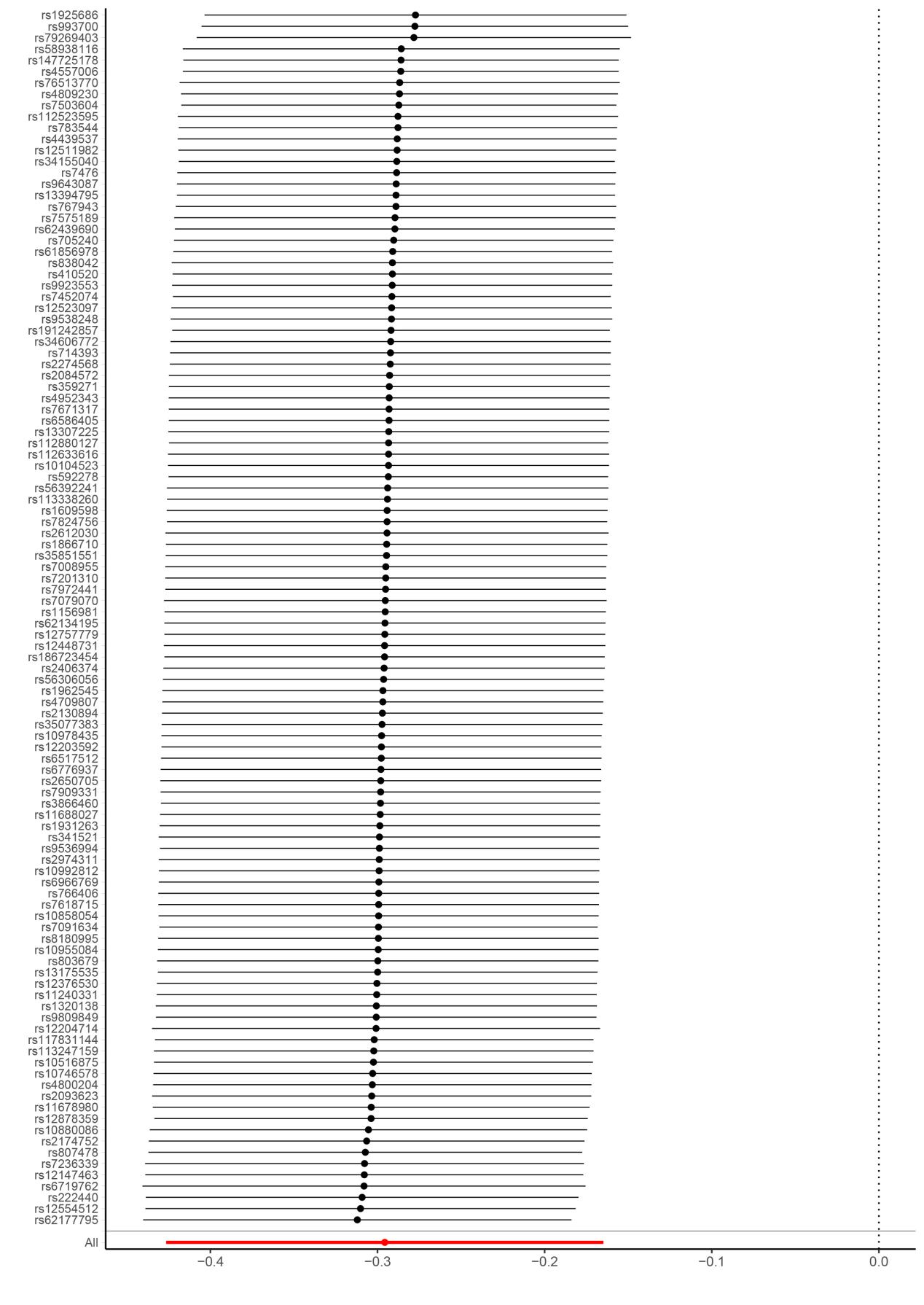


# **Supplementary Figure 23.** MR Leave one out analyses for age at first sexual intercourse on ischemic stroke after removing SNPs associated with confounders or CVDs.


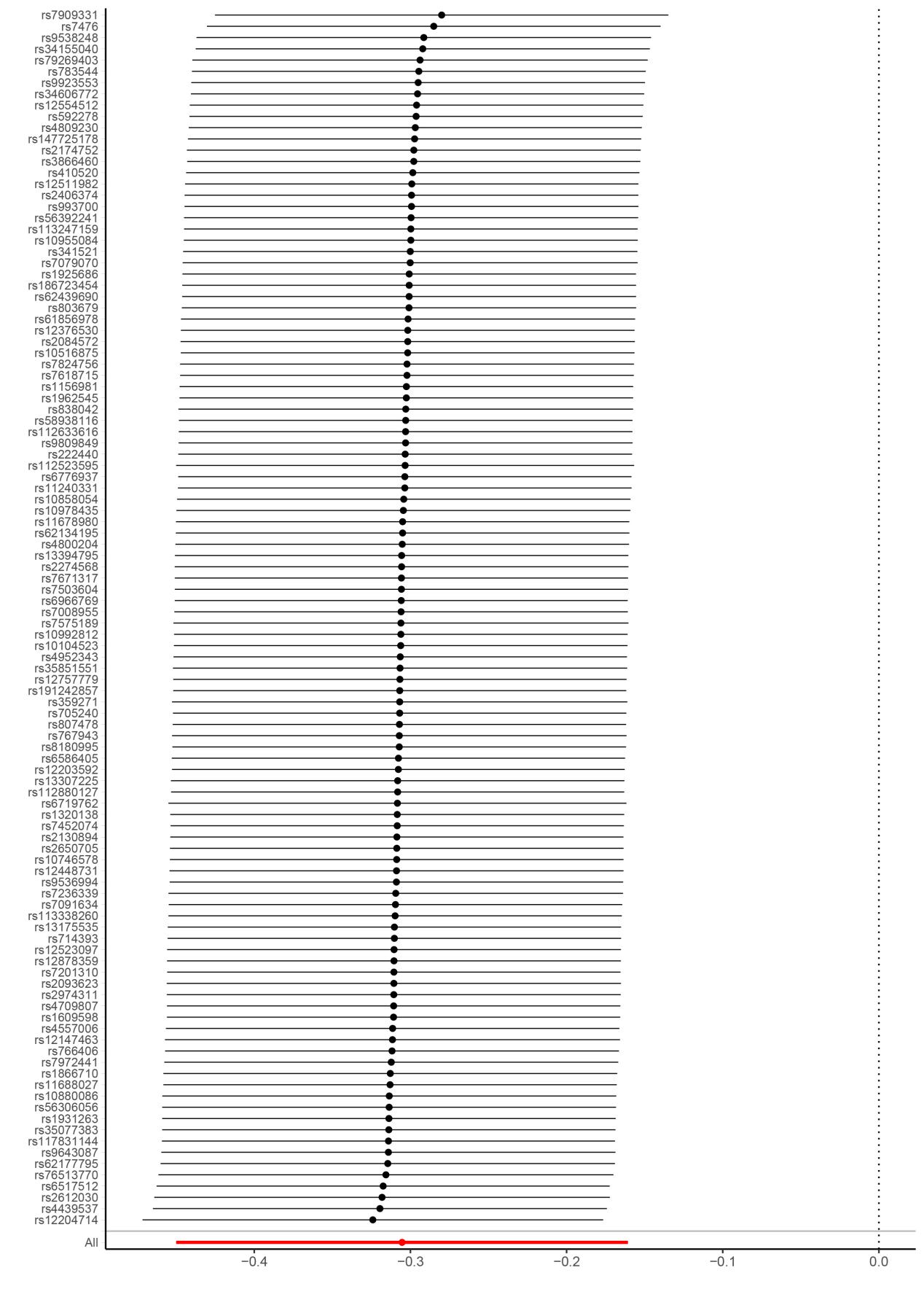


# **Supplementary Figure 24.** MR Leave one out analyses for lifetime number of sexual partners on coronary artery disease after removing SNPs associated with confounders or CVDs.

#
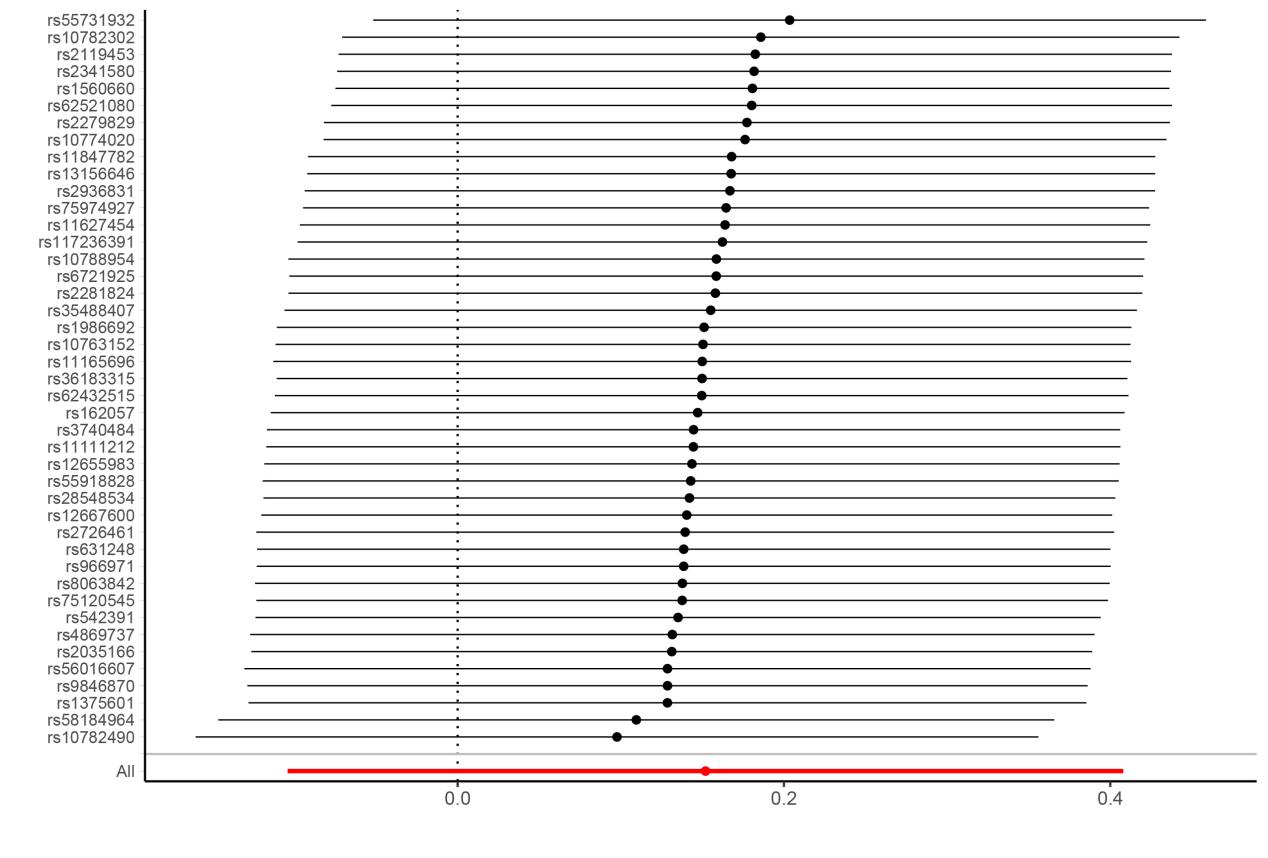


# **Supplementary Figure 25.** MR Leave one out analyses for lifetime number of sexual partners on myocardial infarction after removing SNPs associated with confounders or CVDs.

#
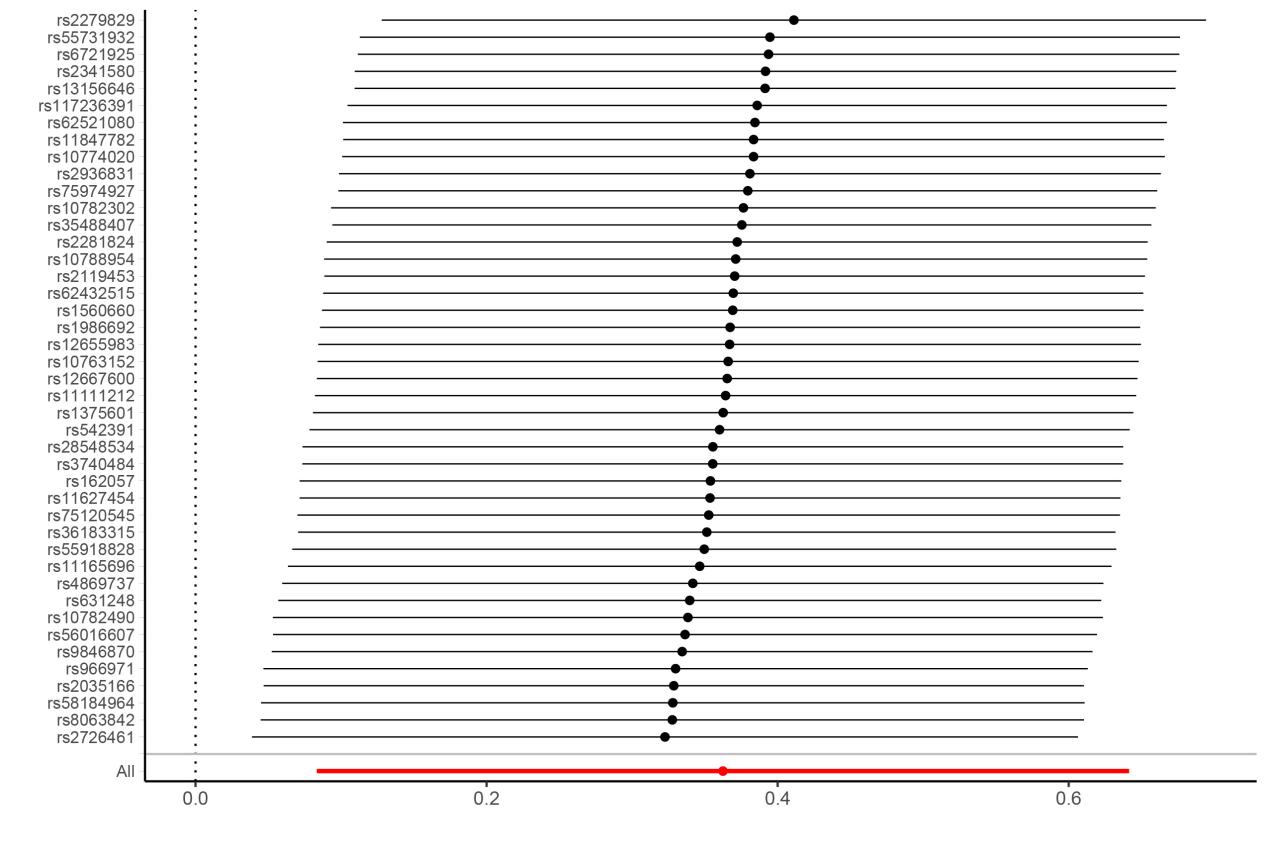


# **Supplementary Figure 26.** MR Leave one out analyses for lifetime number of sexual partners on atrial fibrillation after removing SNPs associated with confounders or CVDs.


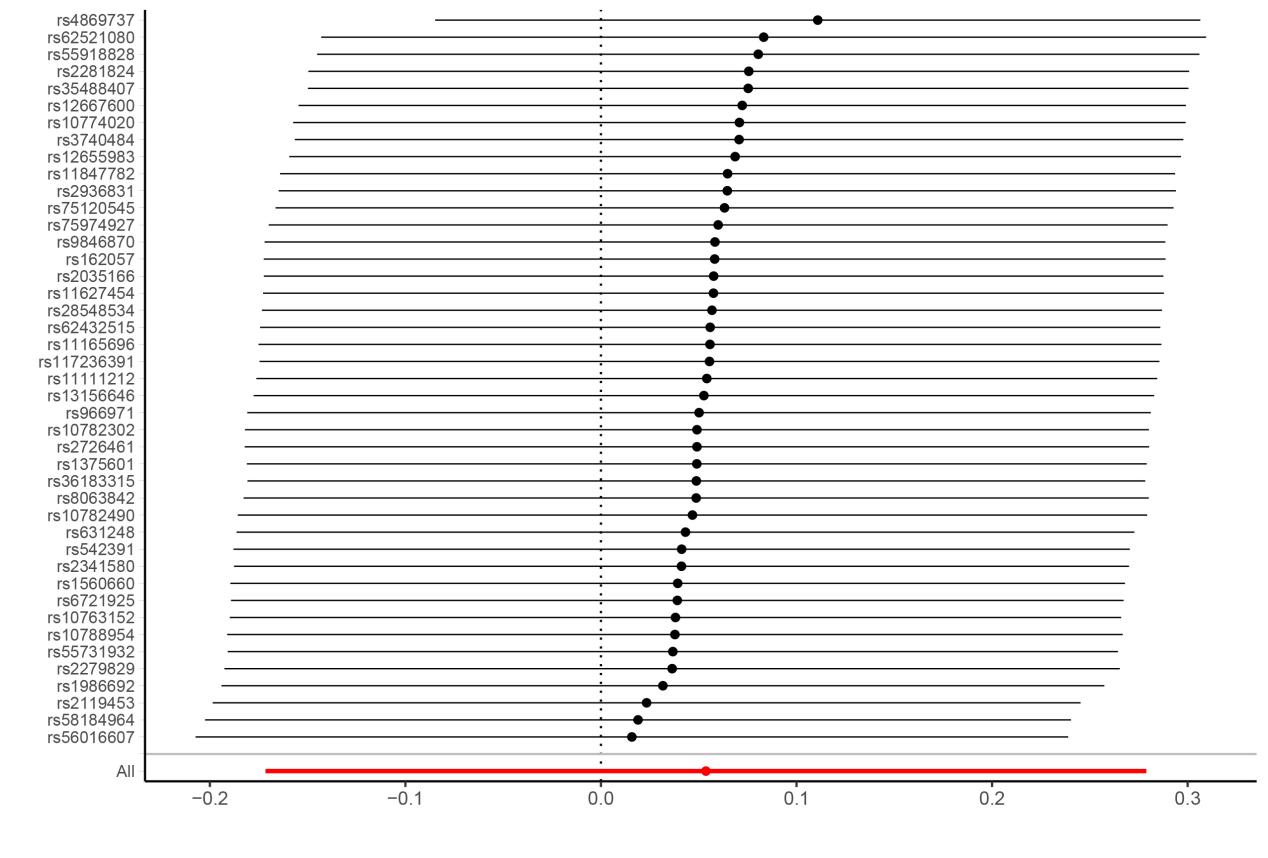


# **Supplementary Figure 27.** MR Leave one out analyses for lifetime number of sexual partners on heart failure after removing SNPs associated with confounders or CVDs.


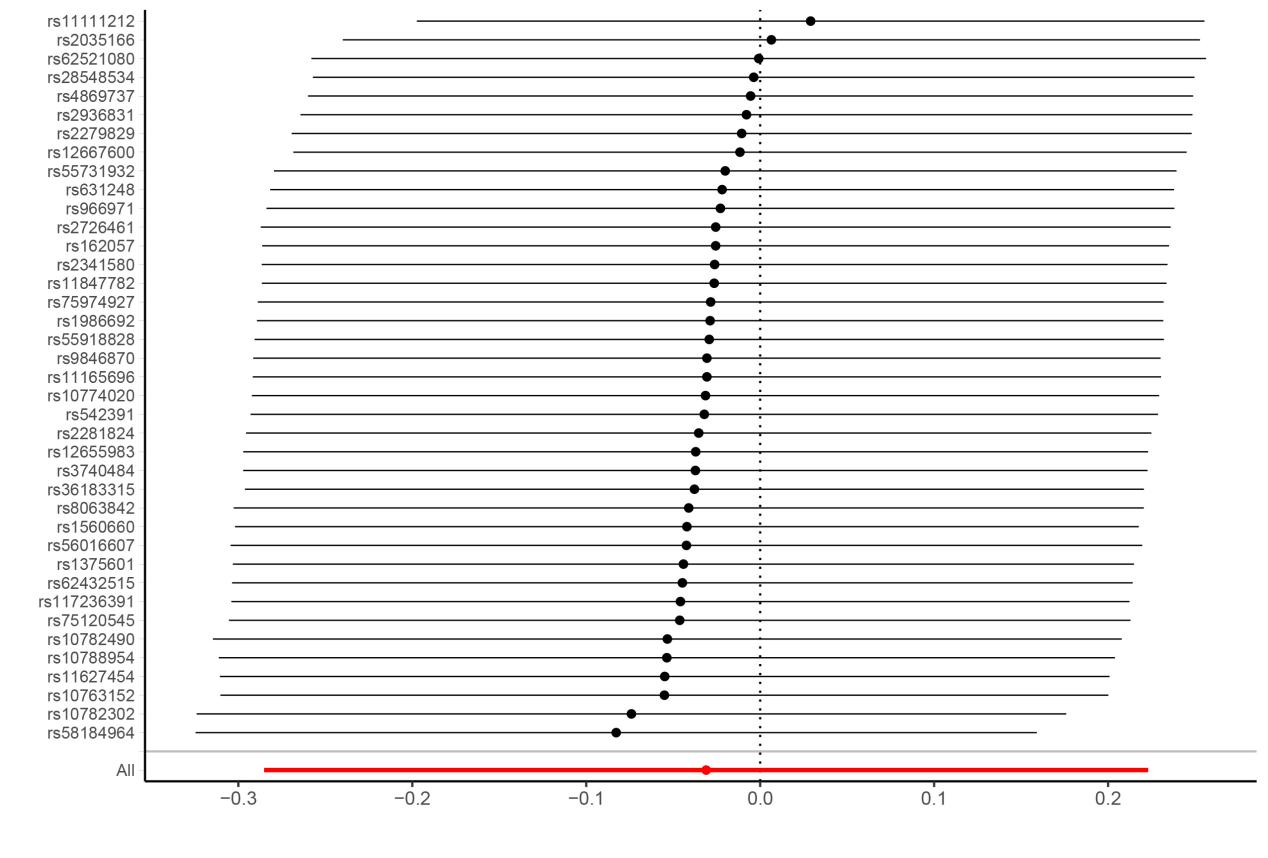


# **Supplementary Figure 28.** MR Leave one out analyses for lifetime number of sexual partners on ischemic stroke after removing SNPs associated with confounders or CVDs.


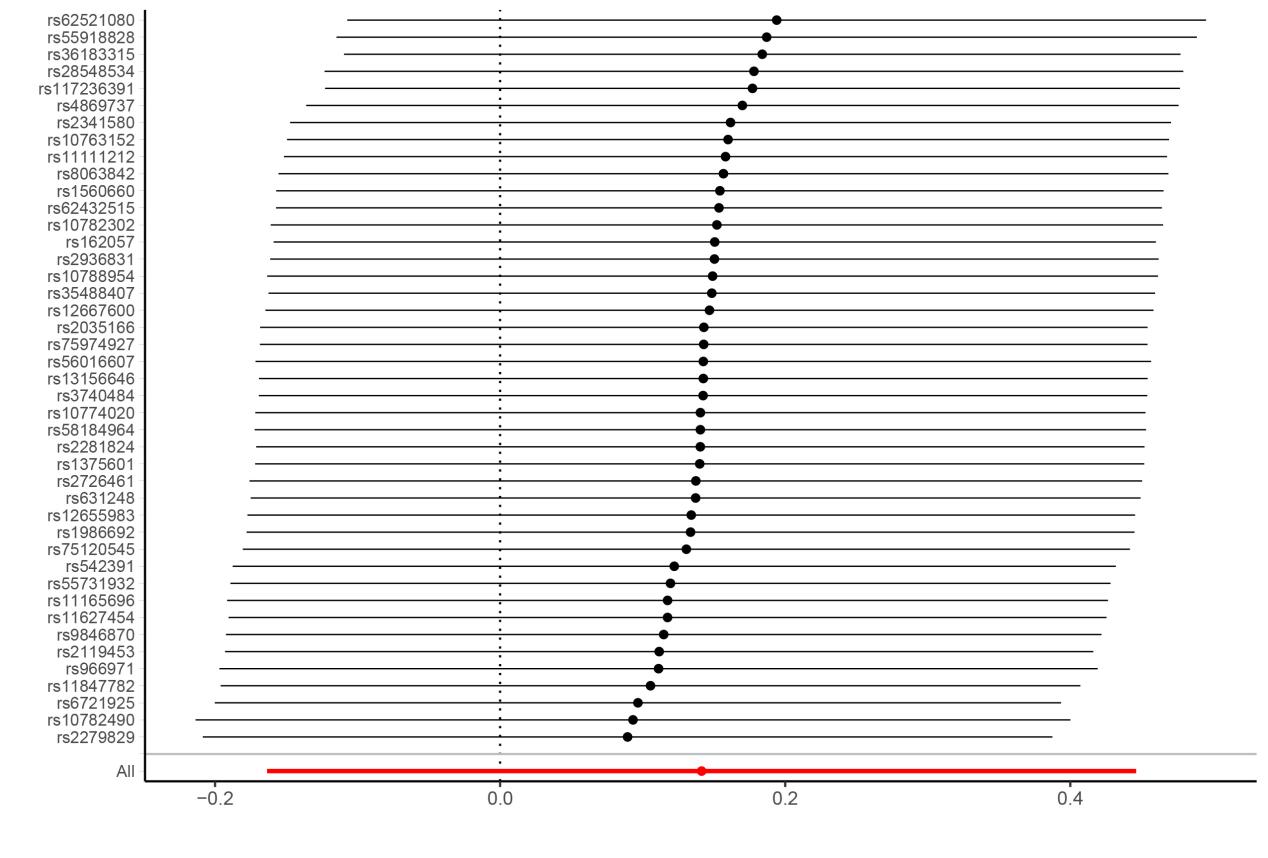

Supplement: Supplementary file 1 [file Table1.docx]
